# Expanding Automated Multiconformer Ligand Modeling to Macrocycles and Fragments

**DOI:** 10.1101/2024.09.20.613996

**Published:** 2024-09-23

**Authors:** Jessica Flowers, Nathaniel Echols, Galen Correy, Priya Jaishankar, Takaya Togo, Adam R. Renslo, Henry van den Bedem, James S. Fraser, Stephanie A. Wankowicz

**Affiliations:** 1 Department of Bioengineering and Therapeutic Sciences, University of California San Francisco, San Francisco, CA; 2 Department of Pharmaceutical Chemistry, University of California San Francisco, San Francisco, CA; 3 Atomwise Inc, San Francisco, CA; 4 Current Address: Department of Molecular Physiology and Biophysics, Vanderbilt University, Nashville, TN

## Abstract

Small molecule ligands exhibit a diverse range of conformations in solution. Upon binding to a target protein, this conformational diversity is generally reduced. However, ligands can retain some degree of conformational flexibility even when bound to a receptor. In the Protein Data Bank (PDB), a small number of ligands have been modeled with distinct alternative conformations that are supported by X-ray crystallography density maps. However, the vast majority of structural models are fit to a single ligand conformation, potentially ignoring the underlying conformational heterogeneity present in the sample. We previously developed qFit-ligand to sample diverse ligand conformations and to select a parsimonious ensemble consistent with the density. While this approach indicated that many ligands populate alternative conformations, limitations in our sampling procedures often resulted in non-physical conformations and could not model complex ligands like macrocycles. Here, we introduce several improvements to qFit-ligand, including the use of routines within RDKit for stochastic conformational sampling. This new sampling method greatly enriches low energy conformations of small molecules and macrocycles. We further extended qFit-ligand to identify alternative conformations in PanDDA-modified density maps from high throughput X-ray fragment screening experiments. The new version of qFit-ligand improves fit to electron density and reduces torsional strain relative to deposited single conformer models and our previous version of qFit-ligand. These advances enhance the analysis of residual conformational heterogeneity present in ligand-bound structures, which can provide important insights for the rational design of therapeutic agents.

## INTRODUCTION

Many biological processes rely on interactions between small molecule ligands and proteins. Small molecule metabolites can be key for regulating natural protein function and small molecule drugs can be used for treating disease by inhibiting or activating proteins. Prior to binding, both the ligand and protein receptor can sample a wide number of conformations. Upon binding, it is typically assumed that both ligand and protein will lose access to nearly all of their conformational states [1]. This assumption leads to the common practice in X-ray crystallography and cryo-EM of modeling the ligand as adopting a single, fixed conformation within the binding site, with little to no consideration of potential heterogeneity other than refined B-factors.

However, a small number of ligands are modeled as multiple conformers in structures deposited in the PDB [2,3]. These structures likely represent just a small fraction of ligands with experimental evidence that could support modeling multiple conformations, as has been shown in proteins [4–6]. Both X-ray crystallography and cryo-EM generate averaged datasets by compiling scattering information from >1000s of system copies, including macromolecules, solvents, ions, and small molecules. Ligand modeling, even as a single conformer, is challenging due to compositional heterogeneity, interference from water molecules, and system-wide conformational heterogeneity, all of which lead to ambiguity in electron density map interpretation [7]. Nonetheless, when handled correctly, modeling ligands in multiple conformations can reveal critical information about biological function [8] and guide small molecule design [9,10].

Conformational and compositional heterogeneity poses significant challenges in both modeling and data processing [11]. Conformational heterogeneity includes subtle, sub-angstrom changes that are difficult to model by eye, yet these shifts are crucial for accurate biological interpretation [4]. This challenge in manual modeling is a major reason why conformational heterogeneity is often underrepresented in structural models. To help assist in modeling conformational heterogeneity, we have developed qFit, which can automatically build multiconformer models in PDB format [6,12]. The underlying concept of qFit is to enumerate a large number of conformations according to a sampling procedure and then to use mixed integer quadratic programming (MIQP) to optimize the selection of a parsimonious set of conformers, along with their corresponding occupancies[12]. This approach improves the fit to experimental data and agreement with geometric priors for proteins [6,13,14], and improves the fit of ligands to experimental data [2].

The previous version of qFit-ligand used iterative sampling over each torsional degree of freedom [2]. This approach overlooked correlated motions and over-explored conformations that were energetically unfavorable. Here we leverage tools that can improve our ability to sample relevant conformations and discard highly strained conformations. We present a redeveloped sampling algorithm powered by the RDKit implementation of the Experimental-Torsion Knowledge Distance Geometry (ETKDG) conformer generator, which is a stochastic search method that combines distance geometry and knowledge derived from experimental structures [15,16]. We demonstrate that qFit-ligand can automatically model multiple conformations of ligands where supported by electron density. The majority of qFit models improved real space correlation coefficients (RSCC) and ligand strain. We also extend qFit-ligand to accommodate two emerging strategies in structure-based drug design, macrocycles and fragments. First, the cyclic nature of macrocycles makes modeling the flexibility by our old approach incredibly troublesome. With improved sampling, we can now model this exciting class of small molecules, which may be capable of targeting ‘undruggable’ protein surfaces [17]. Second, X-ray based fragment screening has exploded in popularity since our first release; however, these approaches rely on density map manipulations accounting for compositional heterogeneity [18], which were poorly handled by our previous version of qFit-ligand. With improved map handling, we can now model into these “event” maps, identifying multiple conformations even for low molecular weight compounds. Together, these advancements and the enhanced code base will enable more accurate identification and modeling of ligand conformational heterogeneity across a variety of ligands, including fragments and macrocycles, leading to a better interpretation of protein-ligand interactions.

## RESULTS

### Overview of the qFit-ligand Algorithm

The qFit-ligand software takes as input a crystal or cryo-EM structure in PDBx/mmCIF format, a density map (encoded by a ccp4 formatted map or an MTZ), and a SMILES string for the ligand. The SMILES string is used for bond order assignment internally. The program produces a multiconformer model of the ligand, embedded into the context of the rest of the unaltered structural model. This version of qFit-ligand leverages advances to the code base that have improved the stability of the code for protein modeling applications [6] and now uses the Chem.rdDistGeom module of RDKit, which implements ETKDG, for conformational sampling [15,16]. As with qFit-protein, following conformer generation, we use quadratic programming (QP) and MIQP optimization algorithms to determine the best fit of the coordinate and occupancy of conformers to the electron density. In qFit-ligand, we set the cardinality constraint to allow selecting up to three conformations for the final ligand model. qFit-ligand is distributed as part of qFit which can be downloaded from Github at https://github.com/ExcitedStates/qfit-3.0 under version 2024.3 and is packaged as part of SBGrid [19].

### Conformer Generation

For an input molecule, the RDKit Chem.rdDistGeom.EmbedMultipleConfs function generates a distance bounds matrix containing the minimum and maximum allowable distances between every pair of atoms for an input molecule (Supplementary Figure 1) [20]. Bounds are set for 1–2 (bonded atoms), 1–3 (bond-angle related atoms), 1–4 (torsion angle related atoms), and 1–5 interactions, based on empirical knowledge of ideal bond lengths and angles from chemical structures. ETKDG is an enhancement of traditional Distance Geometry (DG), implemented within RDKit’s EmbedMultipleConfs function [16]. A key feature of ETKDG is the use of SMARTS patterns to identify torsional substructures in the molecule [21]. Once these torsions are identified, they are stored in a list for later use in the refinement step.

Next, a random set of distances is generated within the allowable bounds, enabling the algorithm to explore conformational space in a stochastic manner. For example, within a torsion angle formed by four atoms, the minimum distance between atoms 1 and 4 corresponds to the syn conformation, and the maximum distance corresponds to the anti conformation. The Chem.rdDistGeom module of RDKit implements ETKDG to uniformly sample distances from the bounds matrix, where for each pair of atoms, a distance is randomly selected within the established bounds. This process generates various conformations of torsional angles, ensuring exploration of the molecule’s conformational space within realistic and chemically meaningful limits.

The sampled distances are converted into three-dimensional coordinates through an embedding procedure. Next, the torsional angles identified via SMARTS pattern matching are refined using torsional potentials which were fit to experimental distributions in the Cambridge Structural Database (CSD) [22,23]. These torsional potentials apply flexible energy functions to guide torsions toward experimentally observed dihedral angle ranges while allowing for some deviation. This step ensures that the generated conformers align with known crystal structure data.

Following torsional minimization, we apply the optionally available force field minimization step, using the MMFF94 force field [24] through the ForceField.rdForceField module, to eliminate steric clashes and reduce molecular strain [15,16]. This procedure ensures that only conformers with low torsional strain are subjected to selection steps to determine the best fitting and most parsimonious ensemble consistent with the electron density map.

### Biasing Conformer Generation

**Figure 1.**
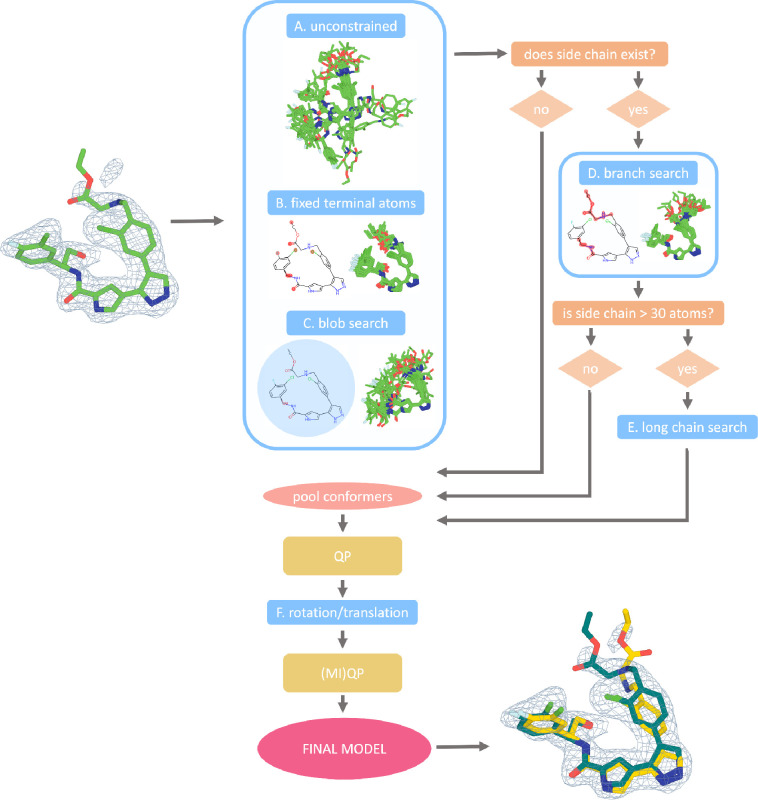
qFit-ligand algorithm workflow. All ligands undergo three preliminary searches: unconstrained, fixed terminal atoms, and blob search, allowing varying degrees of freedom (A-C). If the ligand has short or long side chains, the algorithm progresses to more specialized searches: branch search for ligands with side chains of at least four atoms (D), and long chain search for those exceeding 30 atoms (E). The algorithm then determines the best fit of generated conformers to electron density through quadratic programming, followed by additional sampling with rotations and translations (F). The remaining conformers then undergo quadratic and mixed-integer quadratic programming to ensure that only the most well-supported conformers are included in the final model.

As we only want to generate ligands that physically fit within the protein binding pocket, we bias conformation generation towards structures more likely to fit well in the receptor’s binding site. First, for all ligands, we perform an *unconstrained search function* allowing the generated conformers to only be constrained from the bounds matrix (Figure 1A). This is particularly advantageous for small ligands that benefit from less restriction to fully explore their conformational space. We then perform a *fixed terminal atoms search function* (Figure 1B). This places hard constraints on the distance between the terminal atoms, allowing the atoms in between to randomly sample distances within their respective upper and lower bounds. This preserves the overall shape of the ligand while still allowing for internal movement. Finally, we perform a *blob search function*, which confines generated conformers within a spherical volume, determined by the maximum Euclidean distance from the geometric center of the input ligand to its outermost atoms (Figure 1C).

For ligands with side chains of at least four atoms, we also implement a *branching search function* (Figure 1D). Here, the core atoms, atoms not included in the side chain, are fixed to the coordinates of the input ligand model. This method allows the sampling of side chain conformations while maintaining the relative positioning of the core atoms. When these chains exceed 30 atoms, we apply a *long chain search function*. This approach does the opposite of the *branching search function* by fixing the atoms in the long side chains in place while allowing the core atoms to explore various conformations (Figure 1E). This ensures the generation of relevant conformations of the core atoms without excessive variability in the side chains, which is crucial for ligands with a high degree of freedom.

Additionally, an optional flag turns on the *180-degree flip sampling function*. This function takes the input modeled ligand conformer and rotates it 180 degrees around the three principal axes (x, y, and z). For each axis, the entire ligand is flipped upside down, effectively generating new conformations that are mirror images of the original structure. After each 180-degree flip, the function applies additional rotations within a range of ± 10 degrees in 2-degree increments. This option is only recommended for supervised cases where a user suspects the ligand in their crystal may adopt this specific type of conformational disorder and is turned off by default.

For each run of qFit-ligand, 10,000 conformers are generated, split evenly among each search strategy. As the RDKit Chem.rdDistGeom.EmbedMultipleConfs function generates molecules in a random unit cell, we must align each conformer with the initial input conformer in three-dimensional space. To select the best set of conformers that explain the observed density, qFit-ligand employs a QP optimization algorithm. Each conformer gets a weight (occupancy) that collectively optimizes the real space residual of the observed density versus the weighted sum of all the calculated densities. The algorithm has two constraints, first that all weights are non-negative and that the sum of all weights lies between 0–1. QP usually outputs 1–80 conformations (Methods). We then further sample these remaining conformers by applying rotational and translational perturbations (Figure 1F). New conformations are created by rotating by 15 degrees in 5-degree increments and translating by 0.3 Ångstroms along the x, y, and z axes. Conformers from this stage that fit well with the electron density are then selected through an additional round of QP. The final conformations are then selected using MIQP. With MIQP, the optimization problem is the same (optimizing real space residuals of observed versus weighted sum of all calculated densities), but with additional linear constraints to limit the final multiconformer model to a maximum of three conformers. The output is then one to three ligand conformations with relative occupancies that best explain the observed density (Methods).

### Refinement of qFit-ligand models

qFit-ligand builds a parsimonious multiconformer ligand model and outputs both an independent ligand structure and the protein-ligand complex embedded in the rest of the system (containing solvent, other heteroatoms, etc). After running qFit-ligand, we refine this complex using phenix.refine [25] and delete any conformers with refined occupancies below 10%. The resulting final, refined, model is used for all subsequent comparisons throughout the rest of the paper.

### qFit-ligand runtime

qFit-ligand operates on a CPU, demonstrating efficient performance on a standard laptop with typical runtimes for most ligands (65.91%) being less than 20 minutes (mean: 19.77 minutes, range: 1.88–62.71 minutes). Analysis across a large dataset of structures reveals a strong correlation between the size of the input ligand and the runtime (Pearson correlation coefficient of 0.66), with larger ligands resulting in longer processing times (Supplementary Figure 2).

### Detection of experimental true positive multiconformer ligands

To develop the improved qFit-ligand algorithm, we collected a set of true positive multiconformer ligand models from the Protein Data Bank (PDB). We identified 2,456 PDB files containing ligands with multiple conformations, more than 10 heavy atoms, and resolutions better than 2.0 Ångstroms. We removed 604 common crystallographic additives and carbohydrate ligands, as well as 758 cases where the ligand model’s deposited alternate conformations (altlocs) were not bound in the same chain and residue number. This further pruned our collection to 1,094 structures. We randomly sampled 150 structures and, after manual inspection of the fit of alternative conformations, chose 135 crystal structures as a development set for improving qFit-ligand.

To simulate a realistic scenario where the multiple conformations of a ligand are initially unknown, we retained only the ‘A’ conformations (all structures had 2 conformations), setting its occupancy to 1.00. Occupancy of the ‘A’ conformer was higher than the ‘B’ conformer in 111 (82.2%) of structures. These single conformer ligand structures were refined using phenix.refine [25] (Methods). We refer to these altered structures as our ‘modified true positives.’ We used these modified true positives as input to qFit-ligand, and subsequent refinement (Methods).

To evaluate the impact of qFit-ligand algorithmic improvements, we compared the modified true positive dataset to the output of qFit-ligand (qFit-ligand dataset), evaluating two primary metrics: Real Space Correlation Coefficient (RSCC) and ligand torsion strain (Methods). RSCC evaluates how well the model fits into the electron density, with values exceeding 0.80 indicating a satisfactory agreement between the model and experimental data [26,27]. Torsion strain measures the physical viability of predicted conformations, where lower strain values suggest more stable and naturally occurring conformations. To carry out these strain calculations, we use the software *TLDR: Strain*, [28] which calculates ligand strain by comparing the torsional angle populations of a ligand to those in the Cambridge Structural Database (CSD), quickly assessing strain energy without detailed quantum or molecular mechanical calculations. This is a different strain calculation than what is used internally in RDKit, ensuring that this is an independent metric.

qFit-ligand modeled an alternative conformation in 72.59% (n=98) of structures, with 85.93% (n=116) of structures having a better RSCC in the qFit-ligand models compared to the modified true positive models, representing an improved fit to experimental data in vast majority of structures. Only 7 qFit-ligand structures showed a significant decrease in RSCC (< 0.1). In addition, 77.04% (n=104) of the qFit-ligand models achieved an RSCC of at least 0.80, whereas only 74.81% (n=101) of the modified true positives met this standard.

**Figure 2.**
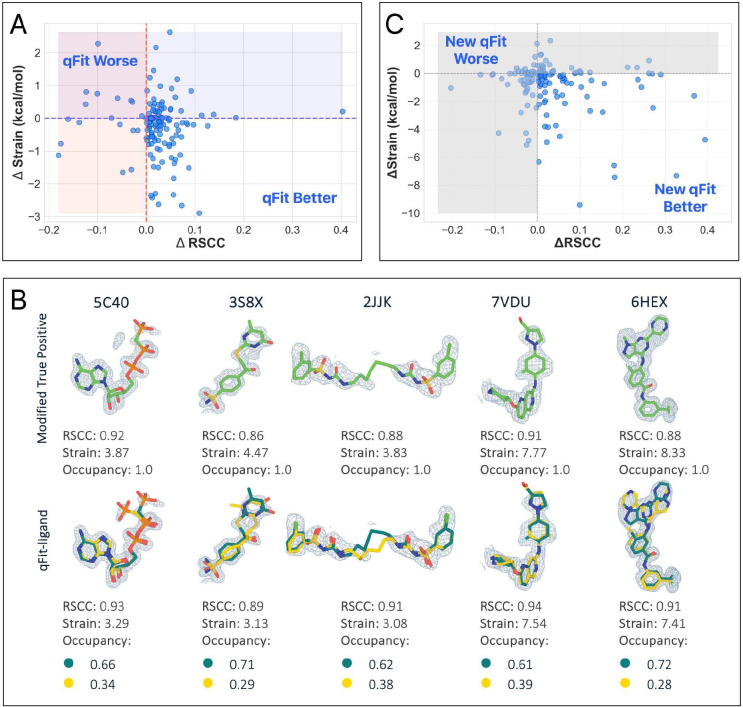
Analysis of ligand conformations generated by qFit-ligand. (A) Differences in RSCC (x-axis) and torsion strain (y-axis) between qFit-ligand predicted structures and modified true positives. The lower right quadrant shows structures for which we improve both RSCC and strain. (B) Gallery of examples for which the updated qFit-ligand models have improved RSCC and strain compared to the modified true positives. The composite omit density map is contoured at 1σ for every structure. (C) Differences in RSCC and torsion strain between the updated qFit-ligand and the original qFit-ligand. The lower right quadrant shows structures for which we improve both RSCC and strain.

Additionally, the majority of structures (60.74%, n=82) exhibited reduced torsional strain, with a mean difference of −0.25 kcal/mol and a median difference of −0.12 kcal/mol (Figure 2A, B, Supplementary Figure 4). This suggests that over half of the qFit-ligand models were more energetically favorable compared to the modified true positive models. When using a single average conformation to describe density from multiple conformations, the true low-energy states may be ignored, resulting in strain. By focusing on fitting conformations that are already low in energy through RDKit, we can have more confidence that the generated models are both realistic and energetically favorable.

Overall, 54.07% (n=73) of ligands had both improved RSCC and reduced torsional strain, demonstrating that we frequently improved the fit between experimental data, while also improving the strain. This indicates that if an alternate conformer exists in the crystal structure, failing to model it could greatly reduce the agreement between the ligand model and the electron density. Additionally, it may introduce significant strain by attempting to fit the electron density with a single conformer when multiple conformers should be modeled.

To identify places for algorithmic improvement, we examined the five outlier structures where qFit-ligand model strain exceeded the deposited model strain by more than 1 kcal/mol. In all cases, the unrefined qFit-ligand model mostly displayed strain levels that matched or improved the modified true positive, but strain was often increased after refinement (Supplementary Figure 5). While refinement improves the correlation between the model and the electron density map, it may inadvertently increase strain without careful calibration of geometry weights and restraint files. This can potentially undermine the energetically favorable conformations initially produced by qFit-ligand, and careful attention should be paid to how refinement impacts strain.

To determine if these changes improved upon the prior version qFit-ligand, we examined the modified true positive dataset. Feeding the same input into the old version of qFit-ligand, we found that the new approach achieved higher RSCC values in 63.70% (n=86) of the structures (Supplementary Figure 6A), and lower strain in 68.15% (n=92) (Figure 2C) (Supplementary Figure 6B, Figure 6C). We closely examined outlier cases, where there were significant differences in results between previous and new qFit-ligand models. Ligand conformational heterogeneity can generally be classified into two categories: localized and non-localized disorder. Localized disorder includes ring flips, where a ring system rotates 180°, and terminal end rotations, where terminal atoms shift positions. Non-localized disorder includes branching ligands, where a side chain adopts an alternate conformation, and displaced disorder, characterized by shifts in all atomic coordinates [2]. In our analysis of outlier cases, we found that most of the deposited true positive models exhibited branching disorder. Here, the new qFit-ligand massively outperforms its predecessor in terms of both RSCC and strain, decreasing strain up to 9.41 kcal/mol and increasing RSCC up to 0.40, highlighting an improvement in our modeling of non-localized conformational disorder. Most notably, PDB 2JJK exemplifies this advancement in modeling branching disorder (Supplementary Figure 7). Compared to the old qFit-ligand model, the new model increased in RSCC by 0.18 and decreased in strain by 7.42 kcal/mol.

Interestingly, among the structures where the old algorithm produced a model with a higher RSCC (n=48), 70.83% (n=34/48) were found to be higher in strain compared to the new models. This suggests that while the old algorithm sometimes provided a better fit to the density, they often did so by compromising on structural or geometric integrity of the ligand. Moreover, of the structures where the old qFit-ligand produced a model with a better RSCC (n=48), only 20.83% (n=10/48) had a new model RSCC lower than 0.80, indicating that the new qFit-ligand models were still generally well correlated to the experimental data. This demonstrates that the new qFit-ligand algorithm strikes a better balance between agreement with the density data and low strain conformations. This directly addresses a major limitation in the old version of qFit-ligand, which often produced conformers that fit the density but were physically or chemically unrealistic, as evidenced by their higher strain.

### Determine the Operational Bounds of qFit-ligand Using Synthetic Data

To determine the lowest ligand occupancy qFit-ligand can accurately recognize and model across resolution ranges, we constructed a synthetic dataset representing four main ligand types (3SC, 3P3, 9BM, AR9). These include a ligand with a ring flip, a long linear ligand with terminal end rotation, one with localized disorder from a simple torsional shift, and a macrocycle. For each ligand type, we designed an alternate conformation in COOT [29] and created synthetic density data across a range of conformer occupancy ratios (0.50/0.50, 0.40/0.60, 0.30/0.70, 0.20/0.80, and 0.10/0.90) and map resolutions (0.8 to 2.5 Å, in 0.1 Å increments) (Methods). This resulted in 360 unique pairs of electron density maps and models, representing various combinations of conformer occupancy and resolution, which we refer to as the “true” structures (Supplementary Figure 8). We then inserted only the “A” conformers into qFit-ligand to evaluate its ability to predict and approximate the “B” conformer for each ligand type.

**Figure 3.**
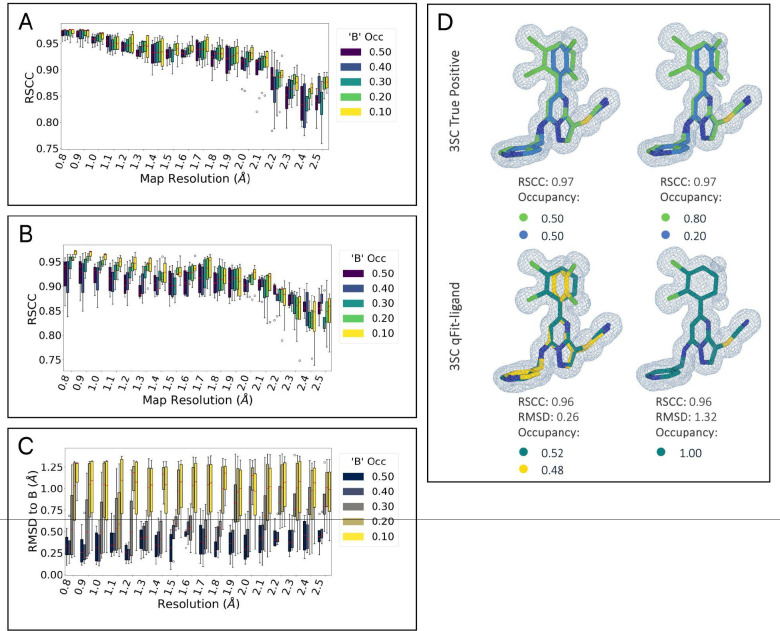
(A) RSCC of the synthetic true benchmark structures plotted against map resolution (in Ångstroms) for different conformer occupancy ratios, showing a decrease in RSCC with deteriorating map resolution. (B) RSCC of qFit-ligand generated multiconformer models, plotted against map resolution and grouped by conformer occupancy split. (C) RMSD between the closest qFit-ligand conformer and the true ‘B’ conformer. (D, left) True structure and qFit-ligand predicted structure of 3SC multiconformer ligand with a map resolution of 0.8 Å and conformer occupancy split of 0.50/0.50. (D, right) True structure and qFit-ligand predicted structure of 3SC multiconformer ligand with a map resolution of 0.8 Å and conformer occupancy split of 0.80/0.20.

We directly compare the RSCC of the output qFit-ligand models with the true structures containing both conformers (Figure 3A/B). We observe a decrease in RSCC as resolution gets worse for all occupancy ratios. As map resolution approaches 2.0 Ångstroms (Å), regardless of the occupancy split, there is a notable decline in qFit-ligand model RSCC. This suggests that qFit-ligand performs most effectively and consistently with map resolutions better than 2.0 Å.

While RSCC quantifies the quality of the overall map to model fit, our ultimate objective is the accurate recovery of alternate conformers, a property that is not directly assessed through RSCC. Therefore, we further utilized RMSD calculations to examine the ability of qFit-ligand to determine the ‘B’ conformer found in the true model. By calculating RMSD between the true ‘B’ conformer and each qFit-ligand generated conformer, we evaluate our ability to register a correct alternate conformation. Our ability to identify a conformer close to the true ‘B’ conformer was correlated with the alternative conformer occupancy (Figure 3C). qFit-ligand models originating from a true model with an occupancy ratio of 0.50/0.50 and 0.60/0.40 exhibit comparable accuracy. Models with a 0.70/0.30 split begin to display marginally higher RMSD values, as well as an increase in inconsistency across map resolutions, though still remaining within acceptable limits. However, models at 0.80/0.20 exhibit greater variability across resolutions, with those at 0.90/0.10 showing even more pronounced inconsistencies. We show an example of the true versus qFit-ligand generated models for the 3SC ligand at a map resolution of 0.8 Ångstroms, with a true model conformer occupancy split of 0.50/0.50 and 0.20/0.80 (Figure 3D). These results suggest that the qFit-ligand occupancy detection limit is around 30%.

### qFit-ligand applied to unbiased dataset of experimental true positives

To determine how qFit-ligand performed on an independent dataset, we curated a new dataset from the initial true positive collection of 1,094 structures, excluding those used in the development set. Recognizing the impracticality of manually inspecting every structure and the detection limit we identified in the synthetic dataset, we applied additional filtering metrics to ensure data quality. Structures were required to have two deposited conformers with a root mean squared deviation (RMSD) of at least 0.2, a ligand B-factor of less than 80, and conformer occupancies of at least 0.3. This process yielded a final set of 318 structures for analysis.

**Figure 4.**
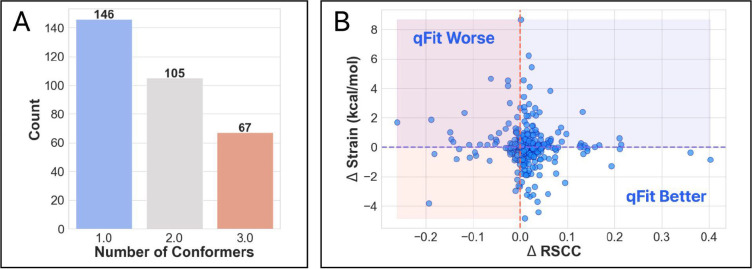
Analysis of ligand conformations generated by qFit-ligand on the un-biased modified true positive dataset. (A) Distribution of the number of conformers output by qFit-ligand. (B) Differences in RSCC and torsion strain between the qFit-ligand and the modified true positives. The lower right quadrant shows structures for which we improve both RSCC and strain.

For all structures, we generated a modified true positive. We then followed the same outline as above, including pre-qFit refinement, qFit-ligand, and post-qFit refinement. The qFit-ligand models yielded 45.91% (n=146) with a single conformer, 33.02% (n=106) with two conformers, and 21.07% (n=67) with three conformers (Figure 4A). Comparing qFit-ligand models to the modified true positives, 75.47% (n=240) showed an enhanced RSCC, reflecting a superior fit to the density map, with only 12 models recording a significant drop (reduction of 0.1). qFit-ligand models had a reduced torsional strain in 50.63% (n=161) of structures, though the overall strain difference was minimal (Figure 4B).

qFit-ligand shows particular strength in scenarios with strong evidence of unmodeled alternate conformations, often improving the fit to density, while sometimes improving the torsional strain. However, qFit-ligand application in cases lacking such strong electron density evidence may potentially lead to a decrease in model-to-map fit quality. Given these findings, it is advisable to employ qFit-ligand selectively, focusing on cases where there are clear indications of unmodeled alternate conformations.

### qFit-ligand can automatically detect and model multiple conformations of macrocycles

While small molecules are great for inhibiting proteins with deep pockets, many proteins with pharmaceutical interests are classified as “undruggable”, due to their flat surfaces or involvement in protein-protein interactions. Macrocycles, cyclic molecules consisting of 12 or more atoms, have a great ability to interact with flat surfaces or shallow grooves, often due to their high degrees of freedom [30–35]. Due to their high degrees of freedom, Macrocycles are highly likely to adopt an ensemble of conformations when bound [36].

With our improved sampling strategy, we wanted to evaluate if we could accurately model multiple conformations of macrocycles. We utilized a dataset of 150 cyclic ligands assembled during the development of XGen, an ensemble-based method for modeling macrocycles [37]. XGen generates ensemble models through restrained force field energy calculations, effectively reducing the strain in these macrocycle coordinate files. XGen captures conformational heterogeneity through an ensemble model approach which encodes multiple complete copies of the entire system that collectively explain the experimental data. In contrast, qFit-ligand represents conformational heterogeneity through a multiconformer approach, labeling discrete parsimonious conformations with alternative location indicators (altlocs). We wanted to determine if we could detect and explain the same conformational heterogeneity as XGen using qFit-ligand and multiconformer models.

All the originally deposited models contain only single conformer ligands. As done above, we pre-refined the deposited models before running qFit-ligand. Of these, 19.33% (n=29) could not be refined against the deposited structure factors and were removed from the analysis. We then ran qFit-ligand as described in the methods section, and re-refined output structures. Refinement is notoriously difficult for macrocycles due to difficulty creating correct restraint files, leading to altered chemical connectivity, effectively changing ligand’s composition. Given that our refinement protocol here was identical to that for non-cyclic small molecules, we conducted post-refinement ligand geometry validation checks to ensure that the chemical connectivity of the ligand remained unchanged, even if the conformation varied (Methods). We identified 18 cases of compromised ligand geometry post-refinement (8 from pre-qFit and 10 from post-qFit refinement), which were subsequently excluded from this analysis. Additionally, strain calculation failed in 20% of cases (n=30/150), producing N/A values, leaving 73 structures available for final analysis. Of note, the strain algorithm used was not developed for macrocycles, so this was not completely unexpected.

**Figure 5.**
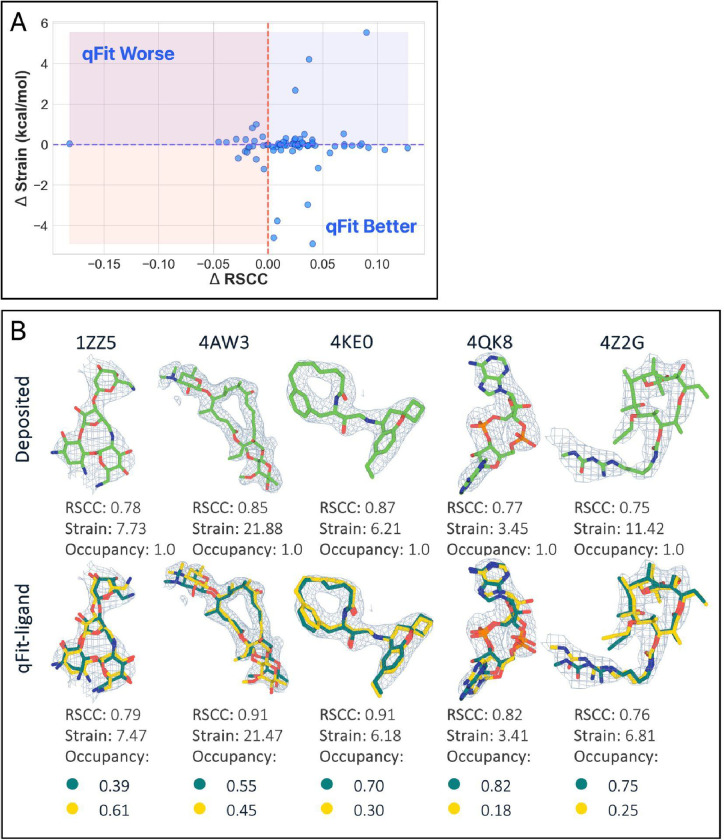
Evaluation of qFit-ligand predicted macrocycle conformations. (A) Differences in RSCC and torsion strain between qFit-ligand predicted structures and refined deposited single conformer macrocycles. The lower right quadrant shows structures for which we improve both RSCC and strain. (B) Gallery of examples for which the qFit-ligand models have improved RSCC and strain compared to the deposited single conformer macrocycle ligand. The composite omit density map is contoured at 1σ for every structure.

Analysis of qFit-ligand outputs for these 73 macrocycles shows the following distribution of conformers per model: 39.73% having one conformation (n=29), 34.25% having two conformations (n=25), and 26.03% having three conformations (n=19) (Supplementary Figure 9A). Compared to the single-conformer deposited models, qFit-ligand improved the RSCC in 75.34% (n=55) of structures, with only one structure having significant decrease (<0.1). We observed a correlation between the number of conformers generated by qFit-ligand and the RSCC of the input model (Supplementary Figure 9B), where a lower input RSCC increases the likelihood of identifying more alternate conformers. Torsion strain analysis showed that 56.16% (n=41) of structures had a lower qFit-ligand model strain, with a mean strain difference of −0.08 kcal/mol. This indicates that, on average, our models maintain a similar level of energetic favorability as the deposited structures, while significantly improving the fit to density (Figure 5).

A few outlier cases have significantly reduced strain in the qFit-ligand models, particularly PDB 4Z2G, which shows a decrease of 4.61 kcal/mol (Supplementary Figure 9C). In this case, qFit-ligand generated two conformers: one identical to the deposited model and a second, distinct conformer. Using COOT’s ligand distortion tool, we compared the strain between the deposited and this distinct qFit-ligand ‘B’ conformer by analyzing each bond and angle [38]. This tool evaluates deviations from ideal geometries based on COD (Crystallography Open Database) data, with restraint dictionaries generated through the AceDRG program [39,40]. A penalty score is calculated using Hooke’s Law, where target values and sigma values from the restraint files are used. The non-bonded interactions are penalized using the Lennard-Jones potential, with atom radii taken from the CCP4 geometry tables. Larger deviations from ideal geometries result in higher penalties, and the overall penalty score is calculated as ${\left(\frac{deviation}{\sigma }\right)}^{2}$, where σ represents the standard deviation of the target value, functioning as the spring constant in Hooke’s Law.

The deposited conformer is highly strained, with the highest bond penalty scores of 71.97 (C_1_-O_2_) and 69.32 (C_14_-O_3_), and the highest angle penalty scores of 29.97 (C_2_-C_1_-O_2_) and 25.5 (O_3_-C_14_-N_1_). The qFit-ligand ‘B’ conformer is significantly less distorted at these locations. For the same bonds and angles, it produces a penalty score of 2.00 (C_1_-O_2_), 0.23 (C_14_-O_3_), 1.15 (C_2_-C_1_-O_2_), and 1.92 (O_3_-C_14_-N_1_) (Supplementary Figure 9C). In the qFit model, the overall strain is lower because alternative conformer ‘A’ is now at partial occupancy. Overall, while qFit-ligand primarily improves RSCC across most models, in a subset of cases, it also significantly reduces strain, demonstrating its ability to enhance both the fit and the energetic favorability of macrocycle conformations.

### Fragment-Soaked Event Maps

X-ray crystallography-based fragment screens have taken off in academic and industry settings [41–44]. Accurately modeling fragments is essential for effective building and merging strategies to create more drug-like molecules. However, as fragments are often bound at low occupancy, modeling into traditional 2F_o_-F_c_ maps is incredibly difficult. To overcome this, ‘event maps’ are often created to detect low occupancy ligands by averaging electron density across many apo datasets and comparing these to the density of a potential ligand bound structure [18]. This produces a ligand binding “event map” and an estimate of the ligand occupancy. Once event maps are created, a modeler must manually fit the single or multiple conformations of the ligand into it. Correctly modeling conformational heterogeneity can drive how fragments are merged or built on. Therefore, we wanted to determine if qFit-ligand could identify multiple conformations in event maps.

To assess qFit-ligand’s ability to detect multiple conformations in event maps, we took advantage of ongoing fragment based drug discovery efforts through the UCSF QCRG Antiviral Drug Discovery (AViDD) program. This project aims to identify potential inhibitors against the severe acute respiratory syndrome-coronavirus-2 (SARS-CoV-2) NSP3 macrodomain [42,45,46]. Through this effort, we identified previously published and new fragments manually modeled with multiple conformations (n=20). Information on chemical synthesis and X-ray crystallography can be found in the Methods. We used these as a true positive dataset to determine if we could identify multiple fragment conformations in event maps.

**Figure 6.**
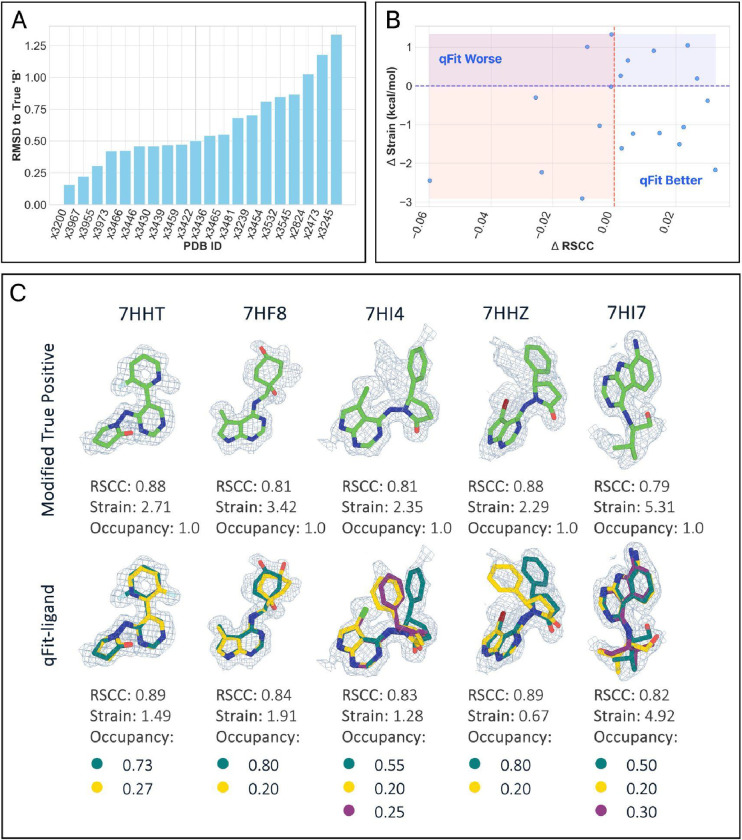
(A) RMSD between the deposited ‘B’ conformer and the closest qFit-ligand conformer. Lower values correlate with a closer recapitulation of the deposited heterogeneity. (B) RSCC and torsion strain differences in the deposited models and the qFit-ligand predicted models. The lower right quadrant shows structures for which we improve both RSCC and strain. (C) Gallery of examples for which qFit-ligand successfully recovers well-fitting alternate conformers. The composite omit density map is contoured at 1σ for every fragment.

We created a modified true positive dataset (n=20) by removing all ‘B’ conformers and setting the ‘A’ conformer occupancy to 1.0. qFit-ligand is then run as described above, but with an event map, rather than a composite omit map (Methods). To determine how precisely we captured the second conformation, we calculated the RMSD between the manually modeled ‘B’ conformer and the closest qFit-ligand conformer for each structure (Figure 6A). 45.00% (n=9) of the structures exhibit an RMSD of less than 0.5 Å, indicating that for approximately half of the cases, our algorithm struggles to recapitulate the second deposited conformer. Of the 55.00% (n=11) of fragments with poor RMSD, about a third (n=4/11) adopted a completely different binding pose, which our current algorithm often fails to capture accurately due to reliance on the input model. This highlights a limitation of our sampling strategy and suggests a potential direction for future development (Supplementary Figure 10, **right**).

To compare the RSCC of the qFit-ligand models to the modified true positive dataset, we used the event maps, rather than scaling to 2Fo-Fc density maps. We found that the RSCC was higher for the qFit-ligand model compared to the modified true positive models in 60.00% (n=12) of structures, with no qFit-ligand models exhibiting significant decreases (<0.1), demonstrating that our approach generally maintains or improves the quality of density fit. More notably, the torsion strain analysis reveals that 65.00% (n=13) of structures have a lower qFit-ligand strain compared to the modified true positive, and 35.00% (n=7) of structures have both a higher qFit-ligand RSCC and a lower qFit-ligand model strain. While the strain difference was not large, a mean strain difference of −0.64 kcal/mol and a median difference of −0.71 kcal/mol (improving in the qFit models), it indicates that we can improve the fit to density without straining the molecule (Figure 6B, 6C).

There are a number of structures for which we calculate an RMSD > 0.5 Å and also an improved qFit-ligand RSCC. In many of these cases, the RSCC improvement is generally very small. In others, we believe they represent situations where multiple combinations of conformations can accurately represent the underlying data. For instance, the qFit-ligand model might generate a flipped Thiophene compared to the deposited model, resulting in a relatively high RMSD to the deposited ‘B’ while still providing an equally good fit to the electron density.

There are only 2 cases where the qFit-ligand model has both a decreased RSCC and increased strain (Supplementary Figure 10). In the first case, we generated a spurious conformer that reduced the model’s agreement with the density map, despite also correctly identifying a conformer close to the deposited ‘B.’ While the algorithm ultimately does not identify the correct conformations, because we are producing a multiconformer model, the user has the ability to delete the extraneous conformation in a molecular modeling software, such as in COOT [29], while keeping the two well modeled conformations. In the second case, we fail to sample the dramatically different binding pose for conformer “B” due to our algorithm’s bias towards the input structure, as discussed above.

In this use case, qFit-ligand models alternative conformations into an event map, which represents only partial occupancy of the unit cell. Therefore, we scale the output ligand conformer occupancies to estimated occupancy from the background density correction prior to merging into the full system. Following this scaling, we perform standard refinement and note that the sum of occupancy across ligand conformations is a refined variable that can be <1.

## DISCUSSION

Although ligands can retain conformational flexibility when bound to receptors, alternative conformations are rarely modeled in deposited structures, potentially leading to misinterpretations of protein-ligand interactions [47,48]. However, accurate modeling of ligands, which must account for significant compositional and conformational heterogeneity, is extremely challenging. Here, we demonstrate that qFit-ligand helps address this challenge by automatically fitting alternative ligand conformations in high-resolution X-ray crystallography maps with clear unmodeled features, improving the model fit to map and reducing ligand torsional strain.

The major improvements presented here stem from incorporating an improved, torsionally aware sampling strategy, with optimization algorithms allowed for the simultaneous improvement of model fit to map and reduction of ligand torsional strain. High ligand strain is energetically unfavorable and can reduce binding affinity; therefore most observed protein-ligand complexes in the PDB are likely to represent relatively unstrained ligands [49,50]. Our work uncovers examples of data that is better fit by multiple conformations, individually with low strain, instead of the deposited highly strained single conformation.

Our improved sampling strategies also allowed us to expand to modeling conformational heterogeneity in macrocycles. Macrocycles offer significant potential for targeting ‘undruggable’ proteins because their exceptional conformational flexibility allows them to interact effectively with relatively flat protein surfaces [35]. This high amount of conformational flexibility must be captured in structural models to ensure correct interpretations. Previous efforts to model macrocycle heterogeneity with XGen used ensemble representations [37]. While xGen reduces strain of ensemble macrocycle models compared to deposited models, ensemble models are complex to analyze, impossible to manipulate in model building software [29], and require specific refinement protocols. The multiconformer models created by qFit-ligand can parsimoniously capture the heterogeneity present in bound macrocycles, often improving RSCC and lowering strain compared to the deposited single-conformer structures.

X-ray crystallography-based fragment screening has also exploded in popularity since qFit-ligand was developed. This increase is largely due to the ability to detect fragments in event maps that enhance signal from low-occupancy binding events [18]. Fragments can potentially bind in multiple conformations due to the small size and promiscuous and weak interactions. We expanded qFit-ligand to automatically model multiple conformations of ligands into fragment event maps, making qFit-ligand a powerful and complementary tool that can seamlessly integrate into fragment identification and modeling workflow. Because of the weak signal in event maps, we emphasize the importance of manual scrutiny of the output conformations for fragments to an even greater extent than for larger, fully occupied ligands.

Despite these advancements, qFit-ligand has room for further improvement. The major successes of qFit-ligand are when there is unmodeled density consistent with a conformation that differs around a torsion or by a small translation. We currently have difficulty identifying larger translations and “ligand flips” that can occur in some binding sites. This limitation is particularly noticeable in the PanDDA dataset, where the deposited multiconformer fragments often exhibit vastly different binding modes. We have added an experimental flag that samples 180 degree flips of ligands; however, this approach should only be used as an exploratory tool where there is a strong visual prior. Our current pipeline handles ligand geometry differently at distinct stages, relying primarily on knowledge-based restraints calculated by different groups for sampling and validation [15,16,28]. However, our minimization and phenix refinement restraints take advantage of force field calculations [24,51,52]. Improving the consistency and accuracy of ligand geometry across these stages would also yield performance improvements [53], especially for macrocycles [37].

Finally, we ultimately strive for modeling the conformational heterogeneity across the entire system including ligands, proteins, nucleic acids, and water molecules. Currently, qFit algorithms allow for modeling either the protein or the ligand separately, focusing on the conformational possibilities of one while treating the other as static [6]. Joint modeling across all system components would generate conformational ensembles that enhance our understanding of how the conformational heterogeneity of each component impacts the other. Beyond the computational modeling advancements, without machine-readable and human-interpretable encoding, we will remain limited in understanding the natural heterogeneity that impacts molecular recognition and drug design [54]. Overall, qFit-ligand provides structural biologists with an efficient tool for modeling parsimonious multiconformer ligand models that fit optimally into electron density maps, reducing the need for manual intervention, aiding in understanding how conformational heterogeneity impacts ligand binding.

## METHODS

### Pre-qFit refinement protocol

Before running qFit-ligand, all input models are stripped of their alt confs, resulting in a set of single conformer coordinate files with ‘A’ ligand occupancies set to 1.0. We use phenix.ready_set (or phenix.elbow if phenix.ready_set fails) to generate cif files for ligand restraint during refinement. All pre-qFit refinement uses the following parameters.


refinement.refine.strategy=individual_sites+individual_adp+occupancies
refinement.input.monomers.file_name=ligand.cif
refinement.main.number_of_macro_cycles=5
refinement.main.nqh_flips=True
refinement.output.write_maps=False
refinement.hydrogens.refine=riding
refinement.main.ordered_solvent=True
refinement.target_weights.optimize_xyz_weight=True
refinement.target_weights.optimize_adp_weight=True
refinement.input.xray_data.r_free_flags.generate=True


After refinement, we generate a composite omit map from the refined model to use as qFit-ligand input.


phenix.composite_omit_map refined_model.pdb data.mtz omit-type=refine nproc=8 r_free_flags.generate=True exclude_bulk_solvent=True


Setting *exclude_bulk_solvent=True* prevents the bulk solvent model from being applied, which typically accounts for disordered solvent by filling low-density areas in the map. When bulk solvent correction is included, it adjusts the electron density by assuming the presence of uniform solvent in regions of low density, such as areas surrounding the ligand. This can reduce the contrast between weak ligand density and the surrounding solvent, potentially smearing or flattening the electron density around flexible or poorly ordered regions like alternative ligand conformations. By excluding bulk solvent correction, you retain the raw electron density in those regions, ensuring the density is not artificially raised or smoothed. This allows clearer visualization of weak or partial densities that might indicate alternative conformers.

### Running qFit-ligand

SMILES strings used as input for qFit-ligand are fetched from the PDB, given the three letter ligand identifier.

To run qFit-ligand on regular small molecules and macrocycles, we used the following command:


qfit_ligand composite_omit_map.mtz refined_pdb.pdb -nc 10000 -sm <smiles string> −l 2FOFCWT,PH2FOFCWT <chain,res_num>


To run qFit-ligand when using an event map, we used the following command:


qfit_ligand event_map.ccp4 input_model.pdb -nc 10000 -sm <smiles string> -l FWT,PHWT -r <resolution> <chain,res_num>


Code for running qFit-ligand is available in our Github repository (https://github.com/ExcitedStates/qfit-3.0) under version 2024.3 and SBGrid (https://sbgrid.org/)

### Post-qFit refinement protocol

After qFit-ligand is run, and before the final refinement, if there are any conformers <0.1 occupancy, they are culled from the output multiconformer model. Again, we use phenix.ready_set (or phenix.elbow if phenix.ready_set fails) to generate cif files for ligand restraint during refinement. All structures are subsequently refined with the following parameters.


refinement.refine.strategy=individual_sites+individual_adp+occupancies
refinement.input.monomers.file_name=ligand.cif
refinement.main.number_of_macro_cycles=5
refinement.main.nqh_flips=True
refinement.refine.adp.individual.isotropic=all
refinement.output.write_maps=False
refinement.hydrogens.refine=riding
refinement.main.ordered_solvent=True
refinement.target_weights.optimize_xyz_weight=True
refinement.target_weights.optimize_adp_weight=True


We then remove and redistribute the occupancy of any conformers with less than 10% occupancy.

If running qFit-ligand on an event map, the refinement process involves an additional step. When using the optional --BDC flag, the script scales the occupancies of the qFit-ligand generated conformers by a factor of (1 - BDC), and produces a new protein-ligand PDB file with the adjusted occupancies. The new PDB file is then processed through the standard refinement protocol, as described above.

### Ligand Geometry Validation of Macrocycles

To validate the geometry of the macrocyclic ligands, we employed a quick check to ensure that the chemical structure had not been altered during refinement. Specifically, we checked that the chemical connectivity of the ligand remained unchanged, even if the conformation varied.

1. Load the PDB file of the protein-ligand complex along with the SMILES string of the bound ligand. The SMILES string represents the correct chemical connectivity of the ligand as it should appear post-refinement.
2. Use RDKit to interpret the SMILES string and attempt to assign bond orders to the ligand in the PDB file. This step compares the intended chemical structure (from the SMILES) with the actual structure after refinement. The bond order assignment is used as a proxy to check if the refinement process altered the ligand’s chemical connectivity.
3. If RDKit successfully assigns bond orders, it suggests that the chemical connectivity has been preserved, and that the refinement process did not improperly modify the ligand’s geometry. However, if RDKit encounters difficulties assigning bond orders, this signals that the refinement may have detrimentally altered the ligand’s structure.

This method serves as a fast, automated “sanity check” to flag potential problems, helping to avoid the need for manual inspection of each PDB file.

### Scoring

QP solvers handle Quadratic Programming problems [55,56]. These problems involve an objective function that is quadratic (a polynomial of degree two) and is subject to linear constraints. The primary goal in the QP framework is to find the combination of conformer occupancies, stored in vector $\omega \;=\;<\omega_{0},\ldots ,\omega_{n}>$, that minimize the difference between the observed electron density and the electron density calculated from the model. Mathematically, this minimizes a residual sum-of-squares function, rss(ω):

$$min_{\text{ω}}\left(rss\left(\text{ω}\right)\right)=min_{\text{ω}}({\left\|{\text{ρ}}^{c}\text{ω}-{\text{ρ}}^{o}\right\|}^{2})$$

$\rho^{o}$ is the observed electron density from the user provided map (target)

$\rho^{c}$ is the weighted calculated electron density from conformers

These occupancies are meaningful parameters, so we require that their sum is within the unit interval, ensuring the total model density does not surpass 100% occupancy.


$$\Sigma \omega_{i}\leq 1$$

Each individual occupancy must be a positive fractional number, meaning each conformer’s contribution is between none and full.


$$0\leq \omega_{i}\leq 1$$

MIQP solvers extend the capabilities of QP solvers by incorporating integer constraints into the optimization problem.

Again, we set up the minimization problem:

$$min_{\text{ω}}\left(rss\left(\text{ω}\right)\right)=min_{\text{ω}}({\left\|{\text{ρ}}^{c}\text{ω}-{\text{ρ}}^{o}\right\|}^{2})\;\Sigma {\text{ω}}_{i}\leq 1$$

Here, we select up to a predetermined number of conformers (cardinality) that meets a minimum occupancy threshold, with all others set to zero. This selection is achieved through mixed-integer linear constraints:

$$z_{i}{\;t}_{min}\leq \omega_{i}\leq z_{i}$$

Where

$$z_{i}\in \{0,\;1\}$$

$t_{min}$ is the minimum-allowable occupancy value for $\omega_{i}$. If $\omega_{i}$ in nonzero, it must be at least $t_{min}$.

The integer constraint limits the number of conformers explicitly. Cardinality is set to three and the minimum occupancy $t_{min}$ set to 0.20, so only up to three conformers can have non-zero weights (of at least $t_{min}$) in the final multiconformer model.

### RSCC

The Real Space Correlation Coefficient (RSCC) is a metric used to assess how well a modeled structure fits into the observed electron density in a crystallographic experiment. It compares the observed electron density values with the electron density values calculated from the model. RSCC values range from 0 to 1, with values above 0.80 generally indicating a good fit. RSCC is calculated using a linear sample correlation coefficient formula.


$$RSCC=coor\left({\text{ρ}}_{obs},\;{\text{ρ}}_{calc}\right)=\frac{cov\left({\text{ρ}}_{obs},\;{\text{ρ}}_{calc}\right)}{\sqrt{var\left({\text{ρ}}_{obs}\right)var\left({\text{ρ}}_{calc}\right)}}=\frac{\sum \;|\;{\text{ρ}}_{obs}\;-\;<{\text{ρ}}_{obs}>\;|\;\sum \;|\;{\text{ρ}}_{calc}\;-\;<{\text{ρ}}_{calc}>\;|\;}{\sqrt{\sum \;{|\;{\text{ρ}}_{obs}\;-\;<{\text{ρ}}_{obs}>\;|}^{2}\;\sum \;{\left|\;{\text{ρ}}_{calc}\;-\;<{\text{ρ}}_{calc}>\;\right|}^{2}}}$$

Where $\rho_{obs}$ is the observed electron density at grid points covering the residue of interest (the input density map), and $\rho_{calc}$ is the density map calculated from the model [57].

For our purposes, RSCC is calculated on the density map’s voxel grid points that correspond to the ligand being modeled. A mask is created around the ligand’s atomic coordinates, and only the density values under this mask’s footprint are extracted. Since the correlation calculation is limited to the observed electron density around the ligand, we effectively answer the question of how well the map fits the model.

Code for calculating RSCC is available on our GitHub repository.

### RMSD

Root Mean Squared Deviation (RMSD) is a widely used metric in structural biology for comparing molecular conformations. It measures the average distance between corresponding atoms of two superimposed structures, and is valuable for assessing differences in conformers, protein structures, and ligand poses.

The RMSD between two sets of atomic coordinates is calculated using the formula:

$$RMSD=\sqrt{\frac{1}{N}\sum_{i=1}^{N}[\left(x_{i}^{\left(1\right)}-x_{i}^{\left(2\right)})+(y_{i}^{\left(1\right)}-y_{i}^{\left(2\right)})+(z_{i}^{\left(1\right)}-z_{i}^{\left(2\right)}\right)]}$$

Where N is the number of atoms, and $\left(x_{i}^{\left(1\right)},y_{i}^{\left(1\right)},z_{i}^{\left(1\right)}\right)$ and $\left(x_{i}^{\left(2\right)},y_{i}^{\left(2\right)},z_{i}^{\left(2\right)}\right)$ are the coordinates of the i-th atom in the two

Code for calculating the RMSD between two conformers of a ligand is available on our GitHub repository.

### Torsion Strain

To calculate molecular strain, we take advantage of software available at http://tldr.docking.org [28].

The TLDR software employs a statistical method based on torsion patterns observed in crystal structures. It identifies all torsions in an input molecule, where each pattern consists of a sequence of four atoms forming a dihedral angle. These patterns are compared against a pre-compiled library of torsion energies sourced from the Cambridge Structural Database (CSD) and Protein Data Bank (PDB).

For each torsion pattern, the software retrieves a histogram of observed dihedral angles and their associated energies. The dihedral angle of the molecule’s conformation is matched to this histogram, and the corresponding energy is determined. This process is repeated for all torsion patterns in the molecule, and the total strain energy is calculated by summing the individual torsion energies.

### Generating a Synthetic Dataset

To create our synthetic dataset, we constructed four multiconformer ligands using COOT [29]. We generated five new PDB files for each ligand, varying the occupancy between the two conformers in the ratios: 0.50/0.50, 0.40/0.60, 0.30/0.70, 0.20/0.80, and 0.10/0.90. These files represent different relative populations of the conformers. For each of these ligand models, we produced a series of electron density maps covering resolutions from 0.8 Å to 2.5 Å, with increments of 0.1 Å using *phenix.fmodel.* This process involves the following steps.

For each given ligand input coordinate file, the script adjusts the B-factors, or temperature factors, of ligand atoms based on the specified resolution. As the resolution degrades from 0.8 Å to 2.5 Å, the B-factors incrementally increase. This adjustment models the increased positional uncertainty of atoms that typically occurs at lower resolutions. The modified ligand structures with these adjusted B-factors at each resolution level are saved as new PDB files. Following this, the script utilizes *phenix.fmodel* to calculate theoretical structure factors from each altered atomic model. These structure factors are then used to compute synthetic electron density maps. The script then processes the .mtz file for each resolution, which contains the calculated structure factors. Random Gaussian noise, scaled proportionally to the resolution, is added to these structure factors. This addition simulates the escalation of experimental noise as resolution deteriorates, a common occurrence in real-life crystallographic data collection.

*phenix.fmodel* is used with the following parameters:


phenix.fmodel input_pdb_file.pdb k_sol=0.4 b_sol=45 high_resolution=<resolution> r_free_flags_fraction=0.05 output.file_name=output_file.mtz


The full script is available at: https://github.com/fraser-lab/qFit_biological_testset

### X-ray Crystallography

Mac1 crystals (P43 construct, residues 3–169) were grown by sitting-drop vapor diffusion in 28% w/v 570 polyethylene glycol (PEG) 3000 and 100 mM N-cyclohexyl-2-aminoethanesulfonic acid (CHES) pH 9.5 as described previously [42,58]. Compounds prepared in DMSO (100 mM) were added to crystal drops using an Echo 650 acoustic dispenser (final concentration of 10 mM) [59]. Crystals were incubated at room temperature for 2–4 hours prior to vitrification in liquid nitrogen without additional cryoprotection. X-ray diffraction data were collected at the Advanced Light Source (ALS beamline 8.3.1) or the Stanford Synchrotron Light Source (SSRL beamline 9–2). Data were indexed, integrated and scaled with XDS [60] and merged with Aimless [61]. The P43 Mac1 crystals contain two copies of the protein in the asymmetric unit (chains A and B). The active site of chain A is open, however chain B is blocked by a crystal contact. We previously observed that potent Mac1 inhibitors dissolve crystals, likely through the displacement of the B chain crystal contact [42]. In addition, crystal packing in the chain A active site restricts movement of the Ala129-Gly134 loop, leading to decreased occupancy for compounds with substituents on the pyrrolidinone. To aid modeling the resulting conformational and compositional disorder, we used the PanDDA method [18] to model ligands where the occupancy was low (<25%) or where there was substantial disorder. After modeling ligands, structures were refined using phenix.refine [62] as described previously [42]. Data collection settings and statistics are reported in Supplementary Table 4.

### Chemical Synthesis

Unless otherwise noted all chemical reagents and solvents used are commercially available. Air and/or moisture sensitive reactions were carried out under an argon atmosphere in oven-dried glassware using anhydrous solvents from commercial suppliers. Air and/or moisture sensitive reagents were transferred via syringe or cannula and were introduced into reaction vessels through rubber septa. Solvent removal was accomplished with a rotary evaporator at ca. 10–50 Torr. Microwave reactions were carried out in a CEM Discover microwave reactor. Chromatography was carried out using Isolera Four flash chromatography system with Silia*Sep* silica gel cartridges from Silicycle.

Reverse phase chromatography was carried out on

- Waters 2535 Separation module with Waters 2998 Photodiode Array Detector. Separations were carried out on XBridge Preparative C18, 19 × 50 mm column at ambient temperature
- Gilson GX-281 instrument (column: Xtimate Prep C18, 21.2*250mm, 150Å, 10μm particle size.

LC/MS data were acquired on

- Waters Acquity UPLC QDa mass spectrometer equipped with Quaternary Solvent Manager, Photodiode Array Detector and Evaporative Light Scattering Detector. Separations were carried out with Acquity UPLCÒ BEH C18 1.7 mm, 2.1 × 50 mm column at 25 °C, using a mobile phase of water-acetonitrile containing a constant 0.1 % formic acid.
- Agilent 1200 Infinity LC with an Agilent 1956 single quadrupole MS using electrospray ionization: Column: SunFire C18 (4.6× 50 mm, 3.5um), Mobile phase: H2O (10 mmol NH4HCO3) (A) / ACN(B), Elution program: Gradient from 10 to 95% of B in 1.5min at 1.8ml/min, Temperature: 50 °CDetection: UV (214, 254 nm) and MS (ESI, POS mode ,103 to 100 amu)

Chemical shifts are reported in d units (ppm). NMR spectra were referenced relative to residual NMR solvent peaks. Coupling constants (*J*) are reported in hertz (Hz). NMR spectra were recorded on Bruker Avance III HD 400 MHz spectrometer or Bruker 500 MHz spectrometer.

#### 4-Chloro-9H-pyrimido[4,5-b]indol-8-amine

A solution 3-fluoro-2-nitroaniline (11 g, 70.51 mmol) in acetic anhydride (20 mL) was stirred at room temperature for 16 hours. The reaction mixture was filtered and the solids were washed with petroleum ether (100 ml) and dried to obtain 10.7 g (77%) of N-(3-fluoro-2-nitrophenyl)acetamide as a brown solid. LCMS (ESI): m/z= 199.3 (M+H)^+^

To a solution of N-(3-fluoro-2-nitrophenyl)acetamide (10.7 g, 54.04 mmol) in DMF (100 mL) was added methyl 2-isocyanoacetate (8.02 g, 81.06 mmol) and potassium carbonate (14.92 g, 108.08 mmol). After stirring at 80°C for 2 hours, the reaction mixture was cooled to room temperature, acidified with 2N HCl (ca. 2000 mL), and extracted with ethyl acetate (300 mL *3). The combined organic layers were washed with brine (100 mL), dried over Sodium sulfate and concentrated under reduced pressure. The residue was purified by silica gel chromatography (10: 1 petroleum ether/ethyl acetate) to obtain 11 g (73%) of methyl 2-(3-acetamido-2-nitrophenyl)-2-isocyanoacetate as a yellow solid. LCMS (ESI): m/z= 278.2 (M+H)^+^

To a solution of methyl 2-(3-acetamido-2-nitrophenyl)-2-isocyanoacetate (11 g, 39.71 mmol) in *glacial* acetic acid (100 ml), was added slowly zinc dust (25.81 g, 397.10 mmol) in two portions. After stirring at 60°C for 2 h, the reaction mixture was cooled to room temperature, filtered and washed with THF. The filtrate was concentrated under reduced pressure and purified by silica gel chromatography (10:1 dichloromethane/methanol) to obtain 6.2 g (63%) of methyl 7-acetamido-2-amino-1H-indole-3-carboxylate as a yellow solid. LCMS (ESI): m/z=248.3 (M+H)^+^

A solution of methyl 7-acetamido-2-amino-1H-indole-3-carboxylate (6.2 g, 25.10 mmol) in formamide (450 mL) was stirred at 220°C for 2 hours. The reaction mixture was then cooled to room temperature and poured in 100 ml of water. The resulting mixture was allowed to stand for 15 min before the solids were collected by filtration, washed with water, and dried to obtain 4.1 g of a 1:2 mixture of N-(4-hydroxy-9H-pyrimido[4,5-b]indol-8-yl)acetamide and N-(4-hydroxy-9H-pyrimido[4,5-b]indol-8-yl)formamide. This mixture was taken in methanol (25 mL) and aqueous 12 N NaOH (25 ml). After stirring at 60°C for 16 h, the reaction mixture was then cooled to room temperature, concentrated under reduced pressure to remove methanol and the residue was poured into 100 mL of water. The resulting mixture was allowed to stand for 15 min before the solids were collected by filtration, washed with water, and dried to obtain 3.5 g (70%) of 8-amino-9H-pyrimido[4,5-b]indol-4-ol as a brown solid. LCMS (ESI): m/z=201.2 (M+H)^+^

A solution of 8-amino-9H-pyrimido[4,5-b]indol-4-ol (3.5g, 17.5mmol) in formamide (30 mL) was stirred at 150°C. After 6 h, the reaction mixture was cooled to room temperature and poured into water (200 ml). The resulting mixture was allowed to stand for 15 min before the solids were collected by filtration, washed with water, and dried to obtain 3.5 g (88%) of N-(4-hydroxy-9H-pyrimido[4,5-b]indol-8-yl)formamide as a brown solid. LCMS (ESI): m/z=229.2 (M+H)^+^

To a solution of N-(4-hydroxy-9H-pyrimido[4,5-b]indol-8-yl)formamide (3.5 g, 15.35 mmol) in phosphorous oxychloride (30 mL) was added N,N-diiisopropylethylamine (5.94 g, 46.05 mmol), After refluxing for 16 hours, the reaction mixture was cooled to room temperature, concentrated and poured into water (20 mL). The resulting solid was filtered to obtain 500 mg of a mixture of N-(4-chloro-9H-pyrimido[4,5-b]indol-8-yl)formamide and 4-chloro-9H-pyrimido[4,5-b]indol-8-amine as a black solid. This mixture was taken in 4 N HCl in dioxane (15 mL). After stirring at room temperature for 4 h, reaction mixture was concentrated under reduced pressure, the residue was adjusted to pH7 with aq.Na_2_CO_3_, and extracted with EA (3 × 30 mL). The organic layers was dried over Sodium sulfate, concentrated under reduced pressure and the residue was purified by reverse phase chromatography (water/acetonitrile /0.1% ammonium bicarbonate) to obtain 320 mg (10%) of 4-chloro-9H-pyrimido[4,5-b]indol-8-amine as a white solid. ^1^H NMR (500 MHz, DMSO) δ 12.42 (s, 1H), 8.74 (s, 1H), 7.58 (d, *J* = 7.8 Hz, 1H), 7.25 – 7.08 (m, 1H), 6.93 (d, *J* = 7.7 Hz, 1H), 5.76 (s, 2H). LCMS (ESI): m/z=219.2 (M+H)^+^

#### AVI-4197/RLA-5830

To a solution of of N-(4-chloro-9H-pyrimido[4,5-b]indol-8-yl)formamide and 4-chloro-9H-pyrimido[4,5-b]indol-8-amine (110 mg, 0.447 mmol), (R)-valinol (69.01 mg, 0.67 mmol) in DMSO (2 ml) was added triethylamine (171.6 mg, 1.41 mmol). After stirring at 100°C for 16 hours, the reaction mixture was extracted with ethyl acetate (3 × 20 mL), washed with brine (20 mL). The organic layer was dried over Na_2_SO_4_. The organic extracts were concentrated and the residue was purified by silica gel column chromatography (50% ethyl acetate/petroleum ether) to obtain (R)-N-(4-((1-hydroxy-3-methylbutan-2-yl)amino)-9H-pyrimido[4,5-b]indol-8-yl)formamide as a white solid (45 mg, yield: 15.2%). LCMS (ESI): m/z=314.3 (M+H)^+^；RT=1.30min

A solution of (R)-N-(4-((1-hydroxy-3-methylbutan-2-yl)amino)-9H-pyrimido[4,5-b]indol-8-yl)formamide (40 mg, 0.13 mmol) in HCl-dioxane (15 mL) was stirred at room temperature for 4h. The mixture was adjusted to pH7 with aq.Na_2_CO_3_, and extracted with ethyl acetate (3 × 30 mL). The organic layer was dried over Na_2_SO_4_, the organic was concentrated and the residue was purified by reverse phase chromatography (0.1%NH_4_HCO_3_ in water, 10%−100% ACN) to obtain (R)-2-((8-amino-9H-pyrimido[4,5-b]indol-4-yl)amino)-3-methylbutan-1-ol (AVI-4197) as a white solid (28.1 mg, yield: 70.52%). 1H NMR (500 MHz, MeOD) δ 8.27 (s, 1H), 7.38 (d, J = 7.8 Hz, 1H), 7.12 (t, J = 7.8 Hz, 1H), 6.82 (d, J = 7.7 Hz, 1H), 4.30 – 4.26 (m, 1H), 3.88 (dd, J = 11.3, 4.8 Hz, 1H), 3.80 (dd, J = 11.3, 4.0 Hz, 1H), 2.17 (d, J = 7.1 Hz, 1H), 1.06 (dd, J = 15.1, 6.8 Hz, 6H). LCMS (ESI): m/z 286.3 (M+H)^+^

#### AVI-3367/RLA-5721

A mixture of 4-chloro-9H-pyrimido[4,5-b]indol-8-amine (28 mg, 0.13 mmol) and 1-aminopyrrolidin-2-one hydrochloride (35 mg, 0.26 mmol) in isopropanol/water (10: 1, 1.1 mL) were heated to 100 °C for 18 h. The reaction mixture was filtered, the residue was washed with ethyl acetate and dried to obtain 28 mg (77%) of 1-((8-amino-9H-pyrimido[4,5-b]indol-4-yl)amino)pyrrolidin-2-one as brown solid. ^1^H NMR (DMSO-d_6_, 400 MHz) δ 12.99 (br s, 1H), 8.62 (s, 1H), 7.92 (br d, 1H, *J*=7.5 Hz), 7.27 (t, 1H, *J*=7.9 Hz), 7.05 (br d, 1H, *J*=7.5 Hz), 3.70 (br t, 2H, *J*=6.9 Hz), 2.44–2.53 (m, 2H), 2.20 (br t, 2H, *J*=7.4 Hz). ^13^C NMR (METHANOL-d_4_, 100 MHz) δ 175.9, 155.9, 154.3, 153.2, 132.5, 125.7, 121.9, 119.4, 111.3, 111.1, 97.0, 48.6, 47.9, 28.5, 15.9. LCMS (ESI): m/z= 283 (M+H)^+^

To a solution of 1-((8-amino-9H-pyrimido[4,5-b]indol-4-yl)amino)pyrrolidin-2-one (15 mg, 0.053 mmol) and triethylamine (0.015 mL, 0.11 mmol) in THF (1 mL), was added ethyl chloroformate (0.005 mL, 0.056 mmol). After stirring at 65 °C for 18 h, the reaction mixture was purified by reverse phase chromatography (water/acetonitrile/0.1% formic acid) to obtain 2.7 mg (13%) of ethyl (4-((2-oxopyrrolidin-1-yl)amino)-9H-pyrimido[4,5-b]indol-8-yl)carbamate formic acid salt (AVI-3367) as tan solid. ^1^H NMR (METHANOL-d_4_, 400 MHz) δ 8.42 (s, 1H), 7.94 (d, 1H, *J*=7.8 Hz), 7.59 (br s, 1H), 7.28 (t, 1H, *J*=7.9 Hz), 4.1–4.26–4.30 (m, 2H), 3.84 (t, 2H, *J*=7.1 Hz), 2.60 (t, 2H, *J*=8.0 Hz), 2.30–2.33 (m, 2H), 1.36–1.39 (m, 3H). LCMS (ESI): m/z= 355 (M+H)^+^

#### (R)-2-((6-Bromo-7*H*-pyrrolo[2,3-*d*]pyrimidin-4-yl)amino)-3-methylbutan-1-ol

To a solution of 6-bromo-4-chloro-7*H*-pyrrolo[2,3-*d*]pyrimidine (900 mg, 3.9 mmol) in dry DMSO (10 mL) was added (*R*)-2-amino-3-methylbutan-1-ol (602 mg, 5.8 mmol) and TEA (787 mg, 7.8 mmol), the mixture was stirred at 110 °C for 16 hours. LC-MS analysis showed the complete consumption of compound 6-bromo-4-chloro-7*H*-pyrrolo[2,3-*d*]pyrimidine. The mixture was diluted with ethyl acetate (40.0 mL) and washed with water (5.0 mL), and brine (5.0 mL). The organic layer was dried over Na_2_SO_4_ and concentrated under reduced pressure. The residue was purified by prep-HPLC (0.1% NH_4_HCO_3_ in water, 10%−100% ACN) to give (R)-2-((6-bromo-7*H*-pyrrolo[2,3-*d*]pyrimidin-4-yl)amino)-3-methylbutan-1-ol as a white solid (522 mg, yield: 45%). 1H NMR (500 MHz, DMSO-*d*6) δ 12.22 (s, 1H), 8.03 (s, 1H), 7.00 (d, 1H, *J* = 8.8 Hz), 6.79 (s, 1H), 4.62 (t, 1H, *J* = 5.2 Hz), 4.13 (s, 1H), 3.52 (dd, 2H, *J* = 9.4, 4.0 Hz), 1.98 (dt, 1H, *J* = 13.6, 6.8 Hz), 0.91 (dd, 6H, *J* = 8.6, 6.9 Hz). LCMS (ESI): m/z=299.2 (M+H)+.

#### AVI-4099 (RLA-5789)

A mixture of (R)-2-((6-bromo-7*H*-pyrrolo[2,3-*d*]pyrimidin-4-yl)amino)-3-methylbutan-1-ol (10.0 mg, 33.4 μmol), 5-(4,4,5,5-tetramethyl-1,3,2-dioxaborolan-2-yl)-1*H*-pyrazole (13.0 mg, 66.9 μmol), Pd(dppf)Cl_2_ (4.9 mg, 6.7 μmol) and CsOH (12.5 mg, 83.6 μmol) in 0.25 mL of mixed solvent (^*n*^BuOH/H_2_O = 4/1) was stirred at 130 °C for 20 minutes with microwave. The residue was purified by prep-HPLC (water, 0%−30% ACN with 0.1% formic acid) to give (R)-2-((6-(1H-pyrazol-5-yl)-7H-pyrrolo[2,3-d]pyrimidin-4-yl)amino)-3-methylbutan-1-ol, formic acid salt (AVI-4099) as a white solid (3.7 mg, yield: 39%). 1H NMR (400 MHz, MeOD) (mixture of rotamers was observed) δ 8.42 (brs, 1H), 8.12 (brs, 1H), 7.73 (d, 1H, *J* = 2.3 Hz), 6.97 (s, 1H), 6.72 (d, 1H, *J* = 2.3 Hz), 4.16–4.11 (m, 1H), 3.84–3.75 (m, 2H), 2.16–2.06 (m, 1H), 1.09–1.02 (m, 6H). LCMS (ESI): m/z=287 (M+H)^+^

#### AVI-4211 (RLA-5849)

A mixture of (R)-2-((6-bromo-7*H*-pyrrolo[2,3-*d*]pyrimidin-4-yl)amino)-3-methylbutan-1-ol (15.0 mg, 50.1 μmol), phenylboronic acid (12.2 mg, 100.0 μmol), Pd(dppf)Cl_2_ (3.7 mg, 5.01 μmol) and Cs_2_CO_3_ (40.8 mg, 125 μmol) in 0.22 mL of mixed solvent (dioxane/H_2_O = 10/1) was stirred at 110 °C for 17 hours. The residue was purified by prep-HPLC (water, 0%−70% ACN with 0.1% formic acid) to give (R)-3-methyl-2-((6-phenyl-7H-pyrrolo[2,3-d]pyrimidin-4-yl)amino)butan-1-ol, formic acid salt (AVI-4211) as a white solid (9.7 mg, yield: 57%). 1H NMR (400 MHz, MeOD) (mixture of rotamers was observed) δ 8.41 (brs, 1H), 8.11 (brs, 1H), 7.79 (brd, 1H, *J* = 8.0 Hz), 7.45 (brdd, 2H, *J* = 8.0, 7.5 Hz), 7.33 (brt, 1H, *J* = 7.5 Hz), 7.03 (brs, 1H), 4.16–4.12 (m, 1H), 3.85–3.75 (m, 2H), 2.15–2.08 (m, 1H), 1.08–1.04 (m, 6H). LCMS (ESI): m/z=297 (M+H)^+^

#### AVI-372/RLA-5628

To a solution of 4-chloro-5-iodopyrimidine (400 mg, 1.66 mmol) in acetonitrile (5 mL) was added 1-aminopyrrolidin-2-one hydrochloride (250 mg, 1.84 mmol) and potassium carbonate (460 mg, 3.33 mmol). The reaction mixture was stirred at 80°C for 1 hour. The mixture was added water (15.0ml) and extracted with ethyl acetate (30 mL *3). The combined organics were washed with brine (10 mL). The organic layer was dried over Sodium sulfate and concentrated under reduced pressure. The residue was purified by silica gel column chromatography (10:1 dichloromethane/methanol) to afford 384 mg (76%) of 1-((5-iodopyrimidin-4-yl)amino)pyrrolidin-2-one. LCMS (ESI): m/z= 305

To a solution of 1-((5-iodopyrimidin-4-yl)amino)pyrrolidin-2-one (20 mg, 0.066 mmol) in 1,4-dioxane (1 mL) was added 2-fluoro-6-(tributylstannyl)pyridine (26 mg, 0.066 mmol), copper (I) iodide (1.3 mg, 0.0066 mmol), triethylamine (0.028 mL, 0.2 mmol) and Pd(PPh_3_)_4_(7.6 mg, 0.0066 mmol). After stirring at 110°C for 18h, reaction mixture was filtered through a celite pad and purified by reverse phase chromatography (water/acetonitrile/0.1% formic acid) to obtain 8 mg (40%) of 1-((5-(6-fluoropyridin-2-yl)pyrimidin-4-yl)amino)pyrrolidin-2-one formic acid salt (AVI-372) as a pale yellow oil. ^1^H NMR (METHANOL-d_4_, 400 MHz) δ 8.71 (br s, 1H), 8.65 (br s, 1H), 8.56 (br s, 1H), 8.34 (br s, 1H), 7.66 (t, 1H, *J*=5.6 Hz), 3.68 (t, 2H, *J*=7.2 Hz), 2.47 (br t, 2H, *J*=8.0 Hz), 2.16–2.20 (m, 2H). LCMS (ESI): m/z=274 (M+H)^+^

#### AVI-411/RLA-5549

A mixture of 4,6-dichloropyrimidine (100 mg, 0.671 mmol, 1.0 equiv), tert-Butyl 5-amino-1H-indazole-1-carboxylate (157 mg, 0.671 mmol, 1.0 equiv) and NEt_3_ (196 uL, 1.41 mmol, 2.1 equiv) in i-PrOH (3mL) was stirred in the microwave at 100 °C for 20 min. The reaction mixture was cooled and evaporated under reduced pressure. The residue was diluted with saturated NaHCO_3_ solution (20 mL) and extracted with EtOAc (3 × 20 mL). The combined organic extracts were washed with water (2 × 20 mL), brine (1 × 40 mL), dried (MgSO4), filtered and purified by silical gel chromatography (0–5% MeOH/DCM) to obtain 30.8 mg (19%) of N-(6-chloropyrimidin-4-yl)-1H-indazol-5-amine as a light yellow solid.

A mixture of N-(6-chloropyrimidin-4-yl)-1H-indazol-5-amine (30 mg, 0.12 mmol, 1.0 equiv) and 1-aminopyrrolidin-2-one hydrochloride (17 mg, 0.12 mmol, 1.0 equiv) in i-PrOH (0.4 mL) was stirred in the microwave at 100 °C for 20 min. The reaction mixture was cooled and evaporated under reduced pressure. The residue was diluted with saturated NaHCO_3_ solution (20 mL) and extracted with EtOAc (3 × 20 mL). The combined organic extracts were washed with water (2 × 20 mL), brine (1 × 40 mL), dried (MgSO4), filtered and purified by reverse phase chromatography (water/MeCN/0.1% formic acid) to obtain 8.1 mg (21%) of 1-((6-((1H-indazol-5-yl)amino)pyrimidin-4-yl)amino)pyrrolidin-2-one as a colorless oil. ^1^H NMR (METHANOL-d_4_, 400 MHz) δ 8.14 (s, 1H), 8.03 (s, 1H), 7.84 (d, 1H, *J*=1.7 Hz), 7.56 (d, 1H, *J*=8.8 Hz), 7.39 (dd, 1H, *J*=1.8, 8.9 Hz), 5.84 (s, 1H), 3.63 (t, 2H, *J*=7.1 Hz), 2.43–2.48 (m, 2H), 2.15 (t, 2H, *J*=7.7 Hz). LCMS (ESI): m/z=283 (M+H)^+^

#### AVI-1495 (RLA-5688)

A mixture of 5-bromo-4-chloro-7*H*-pyrrolo[2,3-*d*]pyrimidine (15.0 mg, 64.5 μmol), 1-(aminomethyl)cyclopropan-1-ol (13.3 mg, 129.0 μmol) in 0.22 mL of mixed solvent IPA/H_2_O (10:1) was stirred at 100 °C for 16 hours. The residue was purified by prep-HPLC (water, 0%−40% ACN) to give 1-(((5-bromo-7H-pyrrolo[2,3-d]pyrimidin-4-yl)amino)methyl)cyclopropan-1-ol (AVI-1495), as a brown solid (6.3 mg, yield: 34%). 1H NMR (400 MHz, MeOD) δ 8.13 (s, 1H), 7.18 (s, 1H), 3.74 (s, 2H), 0.82–0.78 (m, 2H), 0.74–0.71 (m, 2H). LCMS (ESI): m/z=310 (M+H)^+^

#### AVI-3571 (RLA-5703)

A mixture of 4-chloro-5-methyl-7*H*-pyrrolo[2,3-*d*]pyrimidine (15.0 mg, 89.5 μmol), 1-(aminomethyl)cyclobutan-1-ol (18.1 mg, 179.0 μmol) in 0.22 mL of mixed solvent IPA/H_2_O (10:1) was stirred at 100 °C for 4 days. The residue was purified by prep-HPLC (water, 0%−5% ACN with 0.1% formic acid) to give 1-(((5-methyl-7H-pyrrolo[2,3-d]pyrimidin-4-yl)amino)methyl)cyclobutan-1-ol (AVI-3571), formic acid salt as a white solid (7.7 mg, yield: 31%). 1H NMR (400 MHz, MeOD) δ 8.08 (s, 1H), 6.87 (s, 1H), 3.74 (s, 2H), 2.46 (s, 3H), 2.19–2.07 (m, 4H), 1.83–1.75 (m, 1H), 1.70–1.63 (m, 1H). LCMS (ESI): m/z=233 (M+H)^+^

#### AVI-1507 (RLA-5699)

To a solution of 4-chloro-7H-pyrrolo[2,3-d]pyrimidine (70 mg,0.45 mmol) in dry DMSO (5 mL) was added (R)-pyrrolidin-2-ylmethanol (51 mg, 0.50 mmol) and TEA (227 mg, 2.25 mmol), the mixture was stirred at 110°C for 16 hours. The mixture was diluted with ethyl acetate (50.0 mL) and washed with water (10.0 mL), brine (10.0 mL). The organic layer was dried over Na_2_SO_4_ and concentrated under reduced pressure. The residue was purified by prep-HPLC (0.1% NH_4_HCO_3_ in water, 5%−45% ACN) to give (R)-(1-(7H-pyrrolo[2,3-d]pyrimidin-4-yl)pyrrolidin-2-yl)methanol (AVI-1507) as a white solid (35 mg, yield: 35%). 1H NMR (500 MHz, MeOD) δ 8.07 (d, J = 5.4 Hz, 1H), 7.08 (d, J = 3.6 Hz, 1H), 6.66 (d, J = 3.6 Hz, 1H), 4.66 – 4.44 (m, 1H), 3.93 (d, J = 8.8 Hz, 1H), 3.87 – 3.71 (m, 2H), 3.63 (dd, J = 10.9, 6.5 Hz, 1H), 2.21 – 1.99 (m, 4H). LCMS (ESI): m/z= 219.1 (M+H)^+^

## Supplementary Material

Supplement 1

Supplement 2

Supplement 3

Supplement 4

Supplement 5
