# Supplementary material for: Expanding Automated Multiconformer Ligand Modeling to Macrocycles and Fragments
Source: bioRxiv. 2025 Apr 30:2024.09.20.613996. Originally published 2024 Sep 23. Preprint. [Version 2] doi: 10.1101/2024.09.20.613996 (PMC11463535; doi:10.1101/2024.09.20.613996)
Supplement: Supplement 1 [file NIHPP2024.09.20.613996v2-supplement-1.pdf]

bioRxiv preprint doi: https://doi.org/10.1101/2024.09.20.613996; this version posted April 30, 2025. The copyright holder for this preprint (which was not certified by peer review) is the author/funder, who has granted bioRxiv a license to display the preprint in perpetuity. It is made available under aCC-BY 4.0 International license.

Supplementary Figures

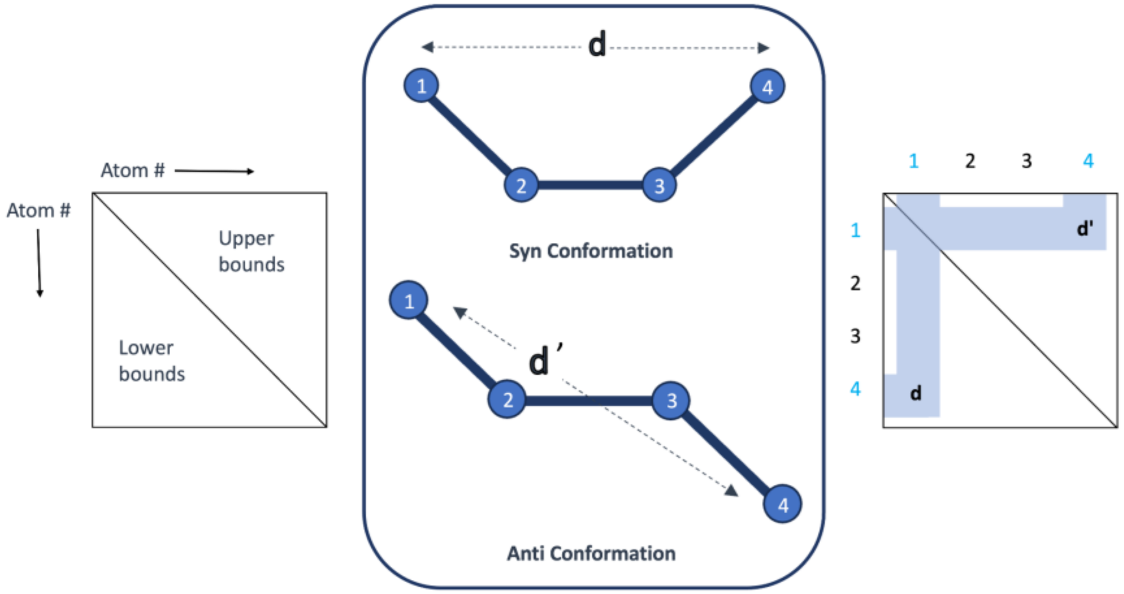

Figure 1—figure supplement 1. RDKit determines a distance bounds matrix for a molecule by establishing upper and lower bounds for interatomic distances. These bounds are informed by experimental data and chemical knowledge of bond length, angle, and dihedral angle preferences obtained from the Cambridge Structural Database. Within a torsion angle formed by four atoms, the minimum distance between atoms 1 and 4 corresponds to the syn conformation, and the maximum distance corresponds to the anti conformation. These specific distances, d for syn and d’ for anti, are recorded in the bounds matrix as the lower and upper bounds, respectively. This is performed for every distance between each atom in the molecule. Randomly sampling these bounds with RDKit’s implementation of ETKDG gives rise to different conformations of the torsion angle.

bioRxiv preprint doi: https://doi.org/10.1101/2024.09.20.613996; this version posted April 30, 2025. The copyright holder for this preprint (which was not certified by peer review) is the author/funder, who has granted bioRxiv a license to display the preprint in perpetuity. It is made available under aCC-BY 4.0 International license.

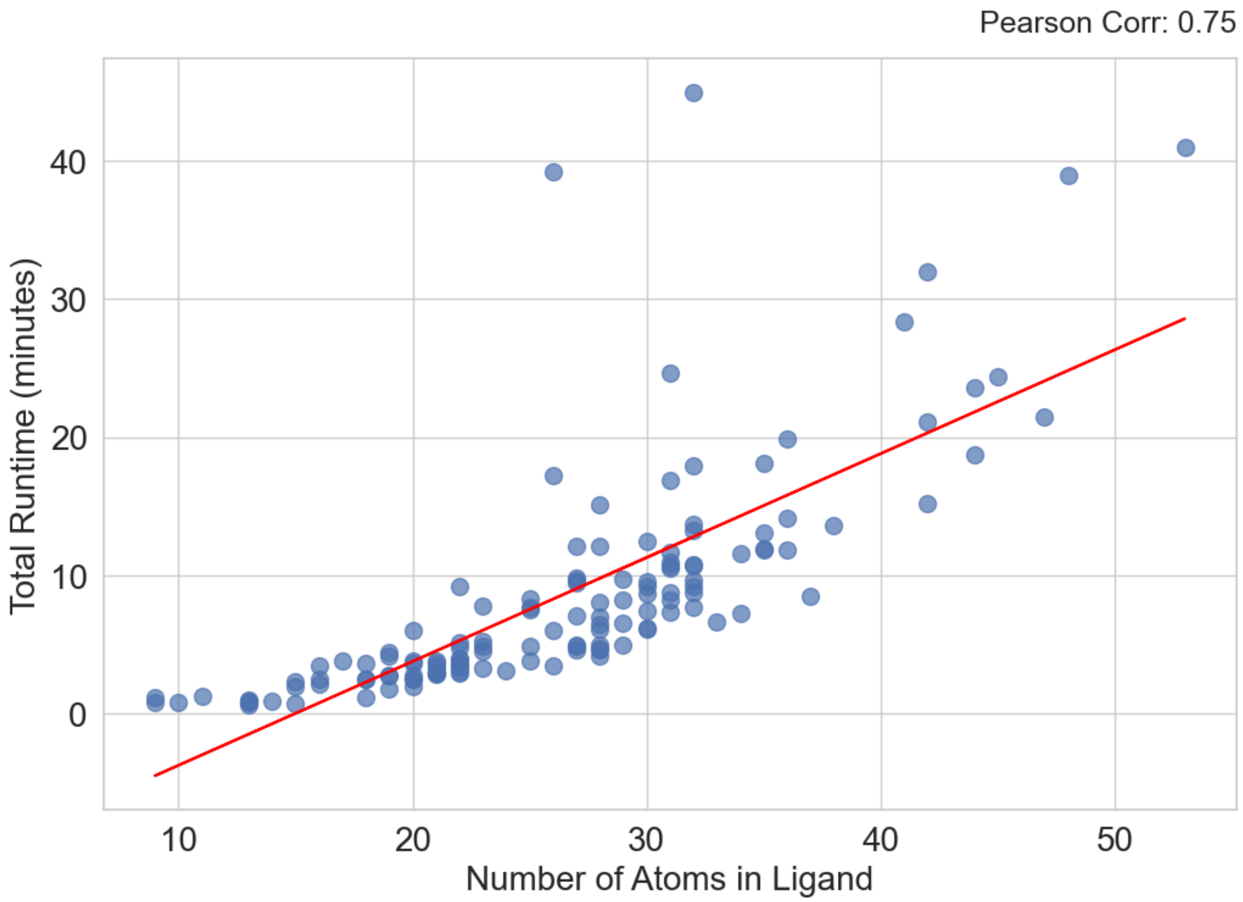

Figure 1—figure supplement 2. Correlation between the number of atoms in the input ligand and total qFit-ligand runtime. A strong Pearson correlation of 0.75 indicates that as you increase the size of your input molecule, qFit-ligand will take longer to run.

bioRxiv preprint doi: https://doi.org/10.1101/2024.09.20.613996; this version posted April 30, 2025. The copyright holder for this preprint (which was not certified by peer review) is the author/funder, who has granted bioRxiv a license to display the preprint in perpetuity. It is made available under aCC-BY 4.0 International license.

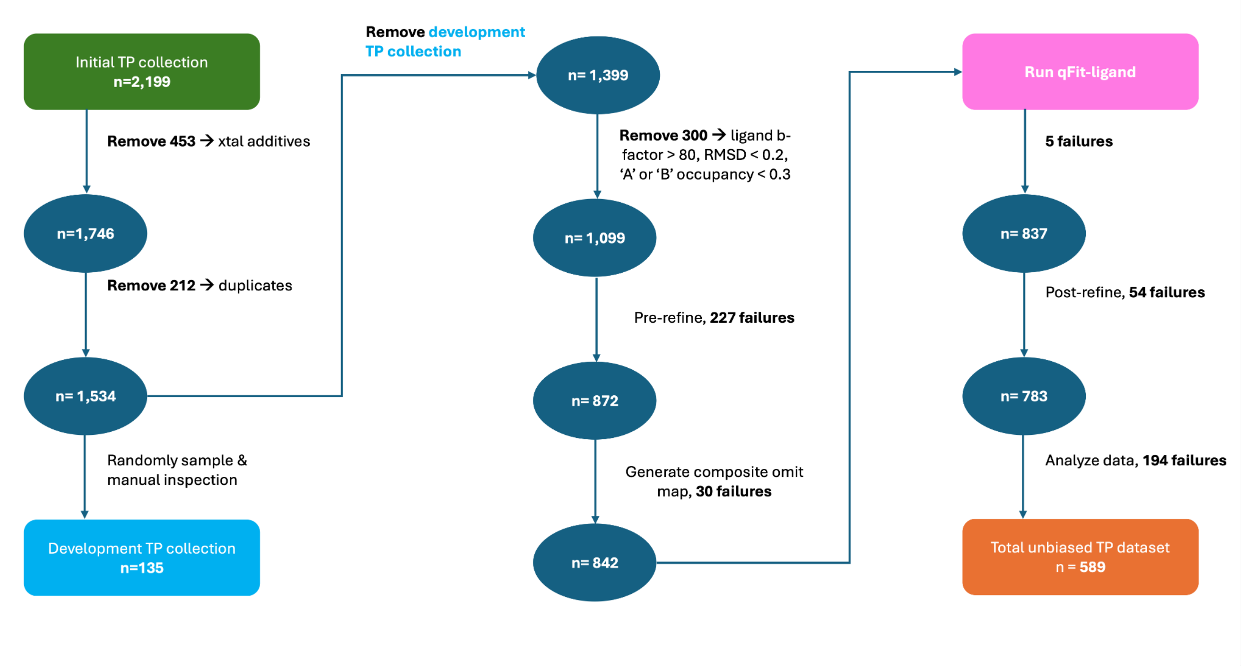

Figure 2—figure supplement 1. Construction of the development true positive dataset and the unbiased true positive dataset.

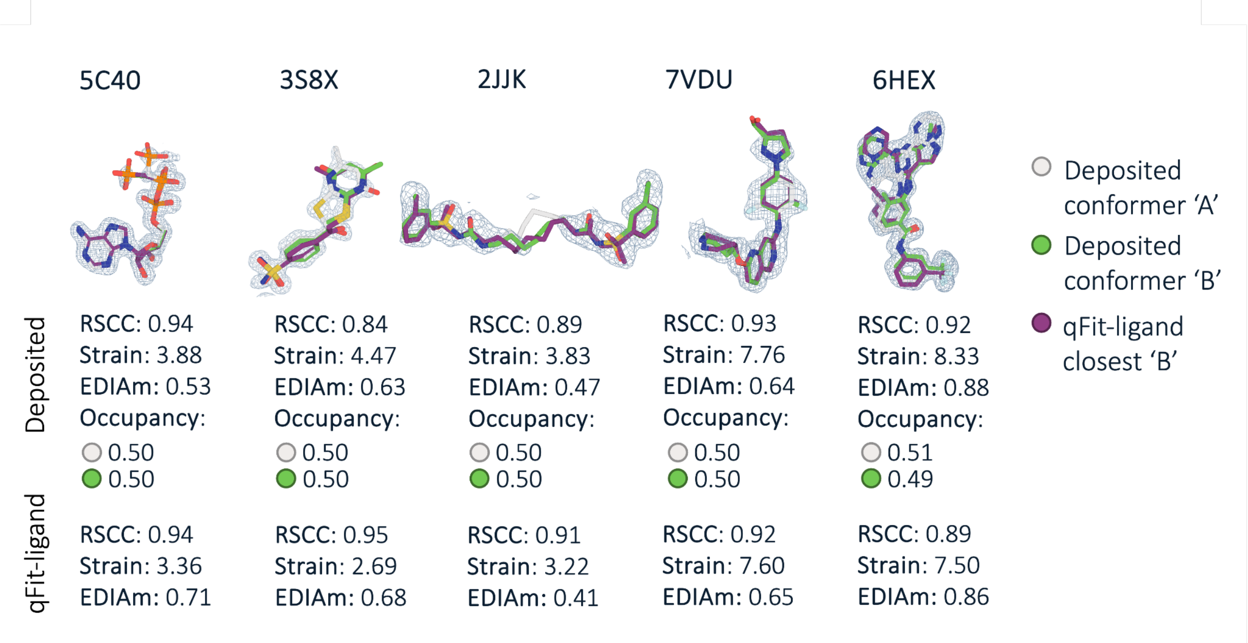

Figure 2—figure supplement 2. Original (unmodified) multiconformer true positives compared to qFit-ligand conformers. The deposited 'A' conformer is shown in gray and the deposited 'B' conformer in green. The qFit-ligand conformer closest to the deposited 'B' is shown in purple. This demonstrates qFit-ligand's ability to accurately recapitulate the original deposited multiconformer model. The composite omit density map is contoured at 1σ for every structure.

bioRxiv preprint doi: https://doi.org/10.1101/2024.09.20.613996; this version posted April 30, 2025. The copyright holder for this preprint (which was not certified by peer review) is the author/funder, who has granted bioRxiv a license to display the preprint in perpetuity. It is made available under aCC-BY 4.0 International license.

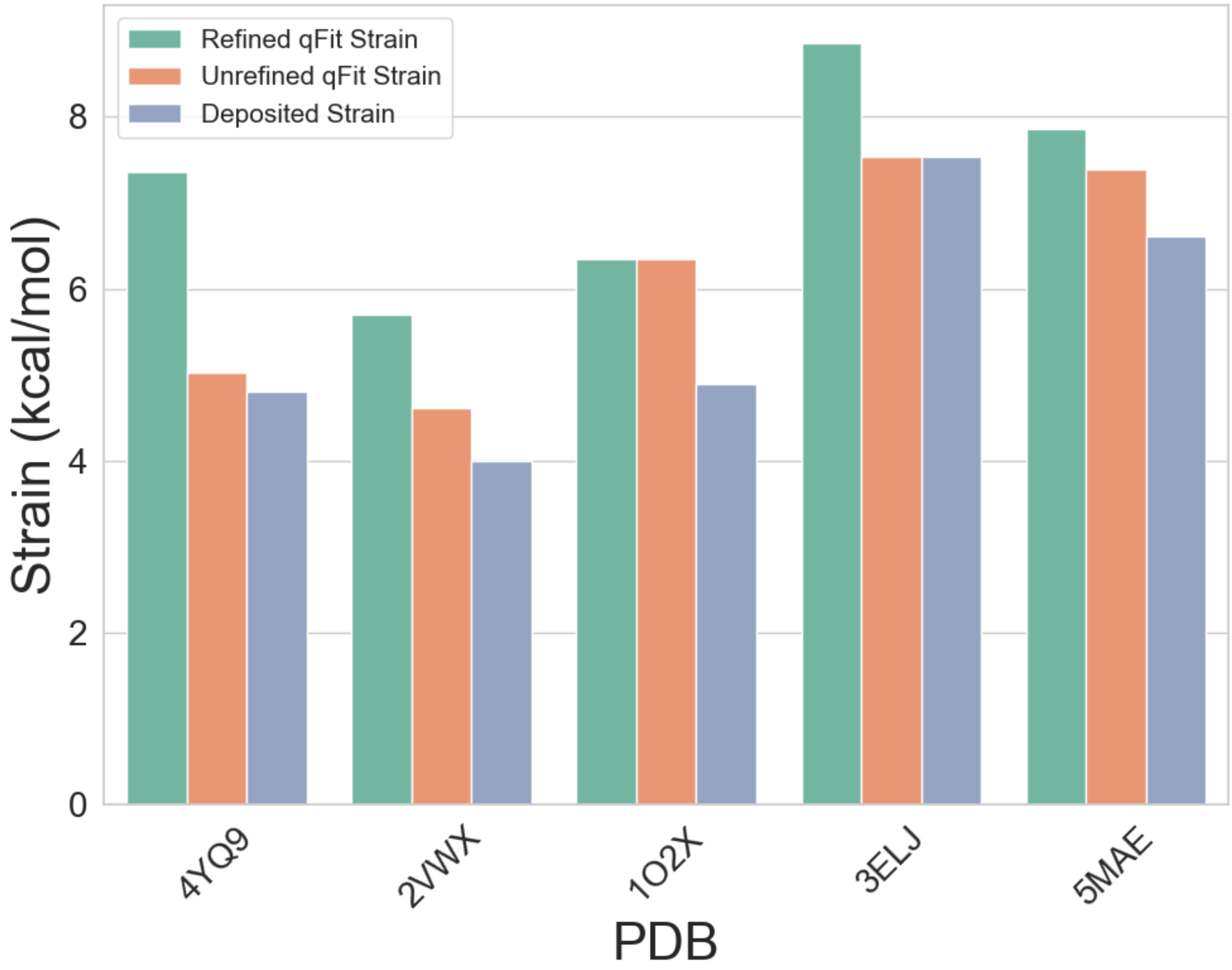

Figure 2—figure supplement 3. Comparison of torsion strain between qFit-ligand models before and after refinement, as well as the deposited structures. The five structures for which the refined qFit-ligand model stain were most significantly increased from the deposited model strain are highlighted.

bioRxiv preprint doi: https://doi.org/10.1101/2024.09.20.613996; this version posted April 30, 2025. The copyright holder for this preprint (which was not certified by peer review) is the author/funder, who has granted bioRxiv a license to display the preprint in perpetuity. It is made available under aCC-BY 4.0 International license.

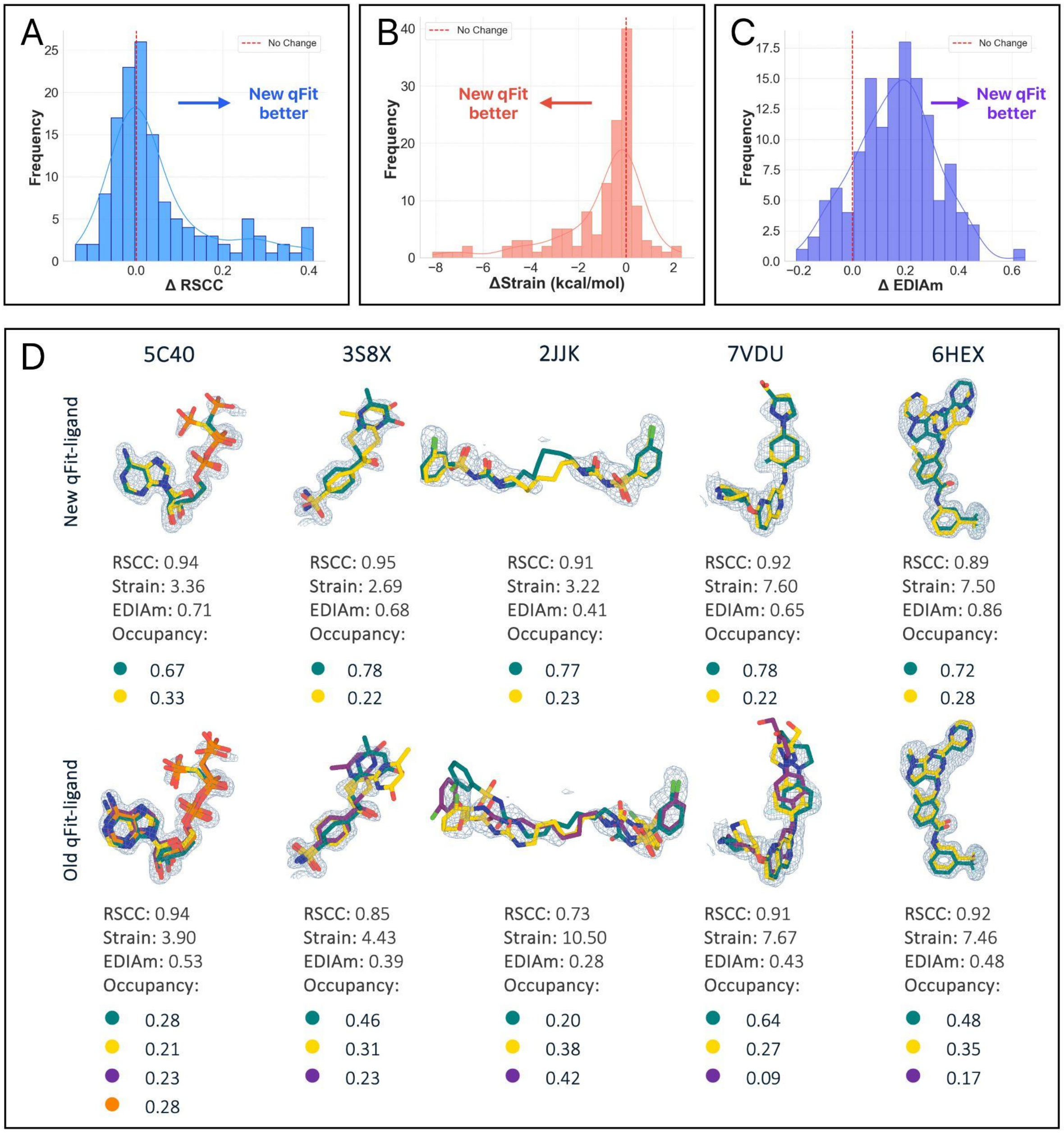

Figure 2—figure supplement 4. Performance comparison of new and prior qFit-ligand algorithms. (A) RSCC of new versus prior qFit-ligand predicted conformations across the true positive dataset. Bars to the right of the vertical line are for structures where the new qFit-ligand model has a higher (better) RSCC. (B) Torsion strain of new versus prior qFit-ligand predicted conformations across the true positive dataset. Bars to the left of the vertical line are for structures where the new qFit-ligand model has improved (lower) internal strain. (C) EDIAm of new versus prior qFit-ligand predicted conformations. Bars to the right of the vertical line are for structures where the new qFit-ligand model has a higher (better) EDIAm. (D) Gallery of examples for which the new

bioRxiv preprint doi: https://doi.org/10.1101/2024.09.20.613996; this version posted April 30, 2025. The copyright holder for this preprint (which was not certified by peer review) is the author/funder, who has granted bioRxiv a license to display the preprint in perpetuity. It is made available under aCC-BY 4.0 International license.

qFit-ligand models are both higher in RSCC/EDIAm and lower in strain compared to the prior qFit-ligand models. The composite omit density map is contoured at 1σ for every structure.

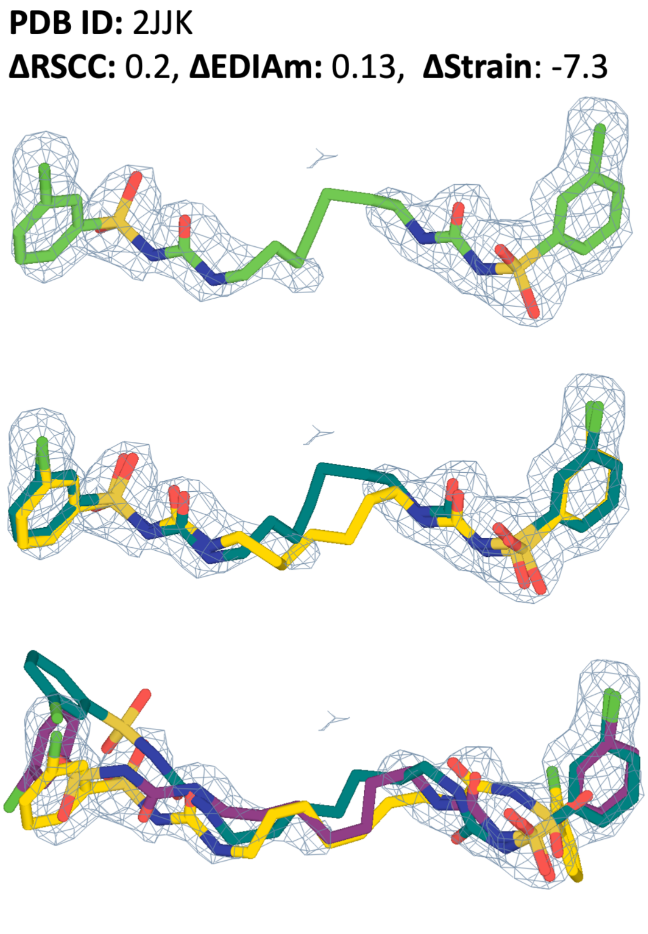

Figure 2—figure supplement 5. Modified true positive dataset comparison of new versus prior qFit-ligand outlier cases. Modified true positive model (input for qFit-ligand), new qFit-ligand model, and prior qFit-ligand model for PDB 2JJK, showing their respective RSCC, strain, EDIAm, and conformer occupancies. Compared to the prior qFit-ligand model, the new model increased RSCC by 0.2, increased EDIAm by 0.1, and decreased strain by 7.3 kcal/mol. The composite omit density map is contoured at 1σ.

bioRxiv preprint doi: https://doi.org/10.1101/2024.09.20.613996; this version posted April 30, 2025. The copyright holder for this preprint (which was not certified by peer review) is the author/funder, who has granted bioRxiv a license to display the preprint in perpetuity. It is made available under aCC-BY 4.0 International license.

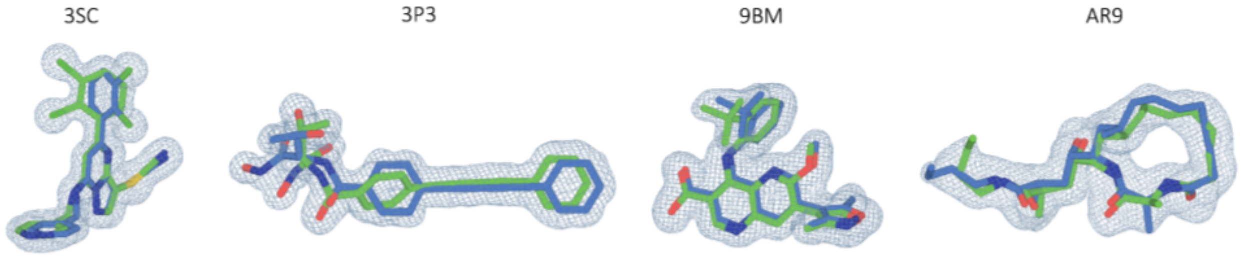

Figure 3—figure supplement 1. The four ligand multiconformer models from which our synthetic dataset was built. Here, they are shown at a map resolution of 0.8 Å at 1σ.

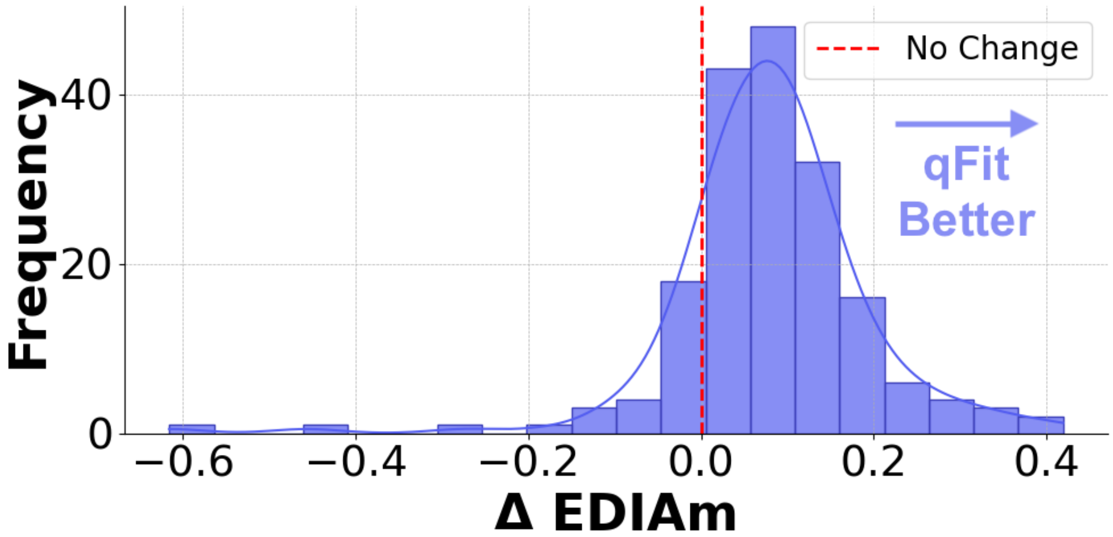

Figure 5—figure supplement 1. Differences in EDIAm between the qFit-ligand models and the refined deposited models. Positive delta values, all bars to the right of the vertical axis, represent structures for which the qFit-ligand model has a higher EDIAm.

bioRxiv preprint doi: https://doi.org/10.1101/2024.09.20.613996; this version posted April 30, 2025. The copyright holder for this preprint (which was not certified by peer review) is the author/funder, who has granted bioRxiv a license to display the preprint in perpetuity. It is made available under aCC-BY 4.0 International license.

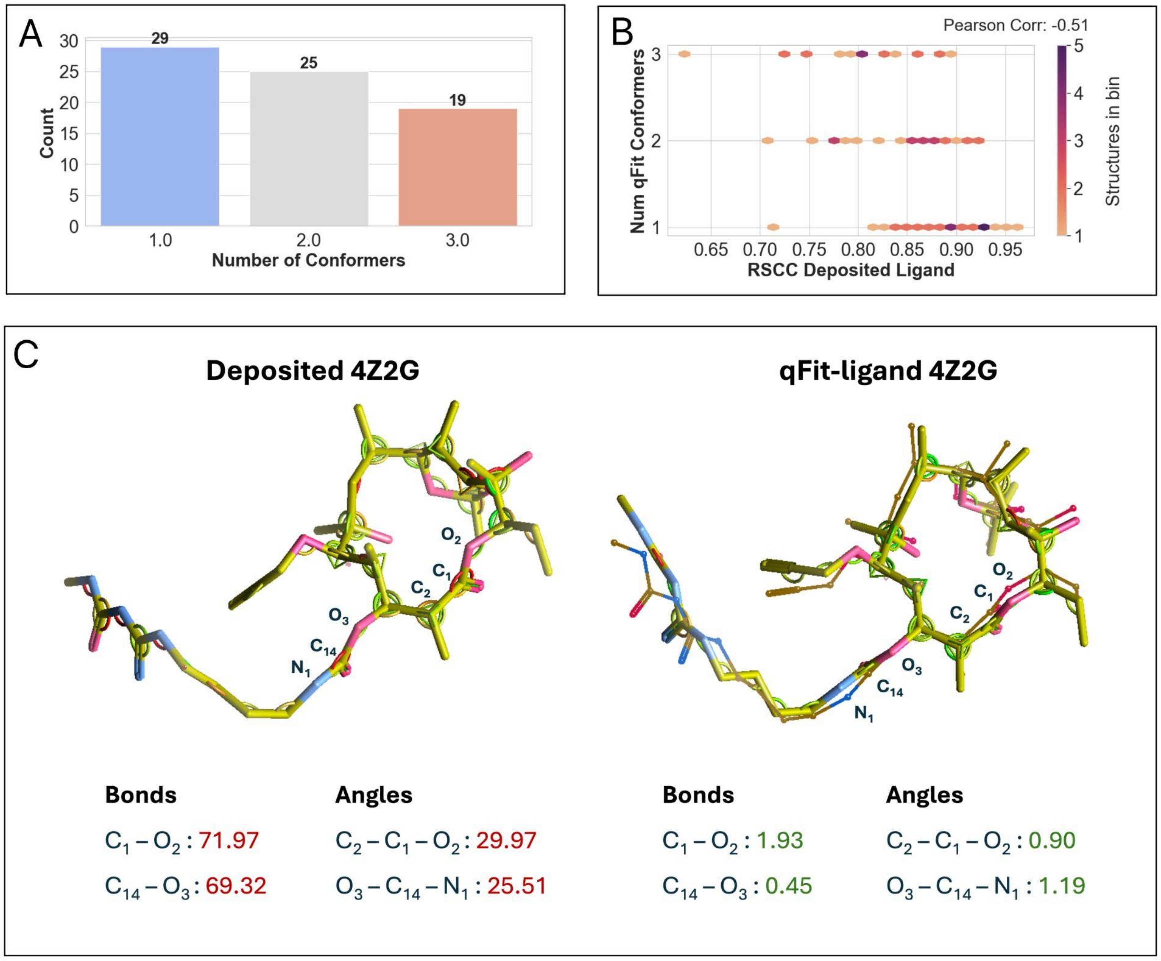

Figure 6—figure supplement 1. (A) Distribution of the number of conformers in qFit-ligand output models, showing varied conformer presence with a median of two conformers per structure. (B) Correlation between the number of conformers output by qFit-ligand and the RSCC of the input model. Higher input RSCC tends to yield a lower number of qFit-ligand conformers. (C) Comparison of strain between the single conformer deposited macrocycle and the qFit-ligand 'B' conformer for PDB 4Z2G using the COOT ligand distortion tool. The penalty scores for the two most distorted bonds and angles in the deposited model (left), compared to the same bonds and angles in the qFit-ligand 'B' conformer (right), demonstrating reduced strain in the alternate conformation. The deposited conformer is highly strained, with the highest bond penalty scores of 71.97 (C_1-O_2) and 69.32 (C_{14}-O_3), and the highest angle penalty scores of 29.97 (C_2-C_1-O_2) and 25.5 (O_3-C_{14}-N_1). The qFit-ligand 'B' conformer is significantly less distorted at these locations. For the same bonds and angles, it produces a penalty score of 1.93 (C_1-O_2), 0.45 (C_{14}-O_3), 0.90 (C_2-C_1-O_2), and 1.19 (O_3-C_{14}-N_1).

bioRxiv preprint doi: https://doi.org/10.1101/2024.09.20.613996; this version posted April 30, 2025. The copyright holder for this preprint (which was not certified by peer review) is the author/funder, who has granted bioRxiv a license to display the preprint in perpetuity. It is made available under aCC-BY 4.0 International license.

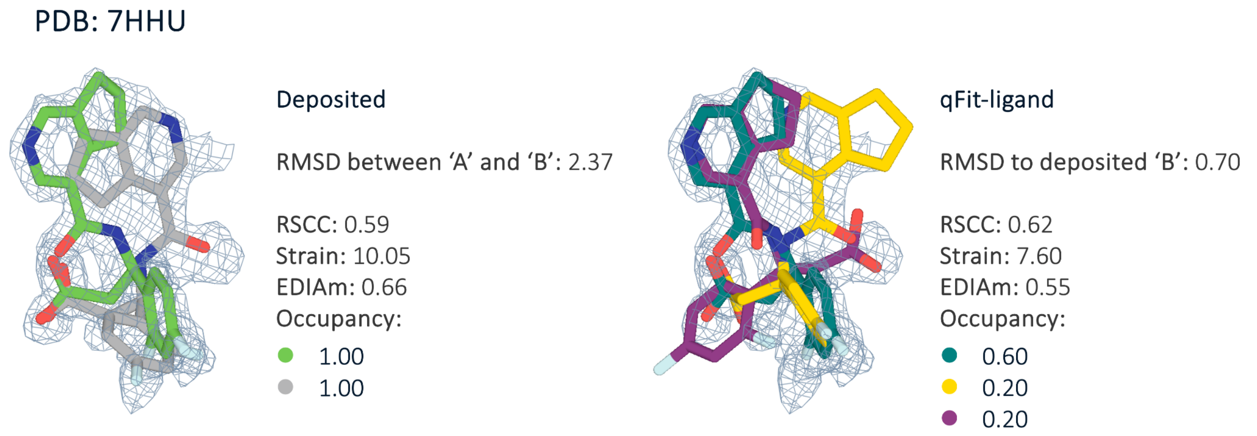

Figure 7—figure supplement 1. PDB 7HHU represents the structure with the highest RMSD between its deposited 'A' (green) and 'B' (gray) conformers. Due to qFit-ligand's sampling bias towards the input structure, we largely fail to recover the known heterogeneity of this sample, with an RMSD of 0.70 Å between the qFit model and the deposited 'B'. qFit RSCC and strain show improvements over the single conformer modified true positive, but EDIAm worsens. The event density map is contoured at 1σ for both structures.

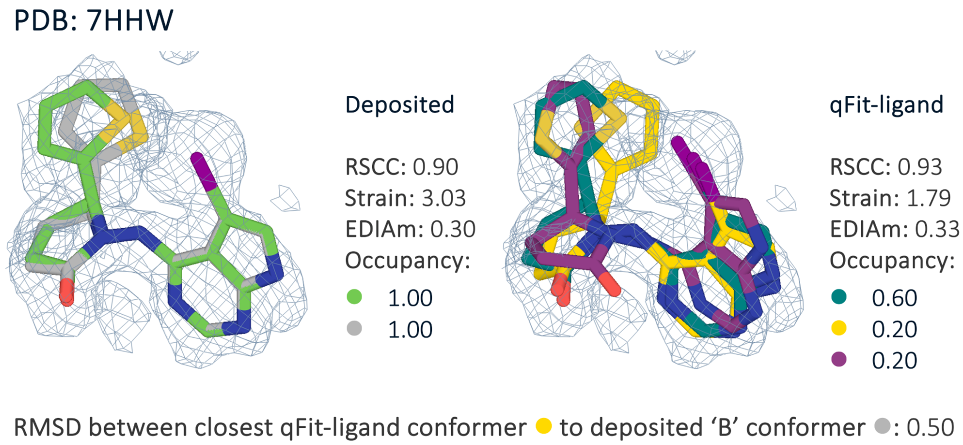

Figure 7—figure supplement 2. Comparison of deposited conformers 'A' (green), 'B' (gray) and qFit-ligand conformers for PDB 7HHW. qFit-ligand generates a multiconformer model with a relatively high RMSD (0.5 Å) to the deposited 'B' conformer. The closest qFit-ligand conformer (yellow) shares an extremely similar atomic space with the deposited 'B', differing primarily due to a flipped Thiophene, resulting in the high RMSD. Despite this, the qFit-ligand model has an improved fit to the electron density, indicating that multiple distinct conformations can accurately explain the experimental data.

3SC

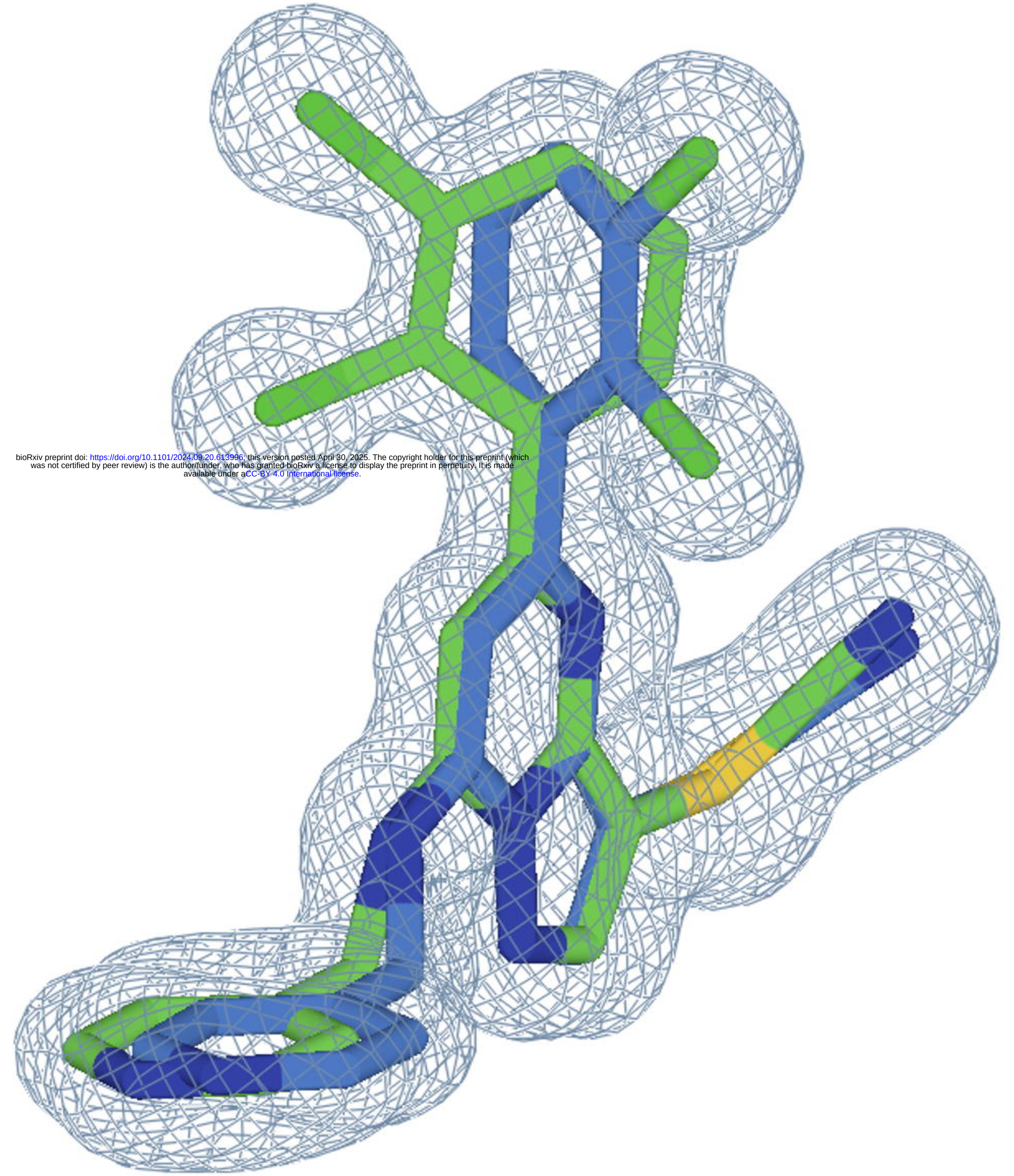

3P3

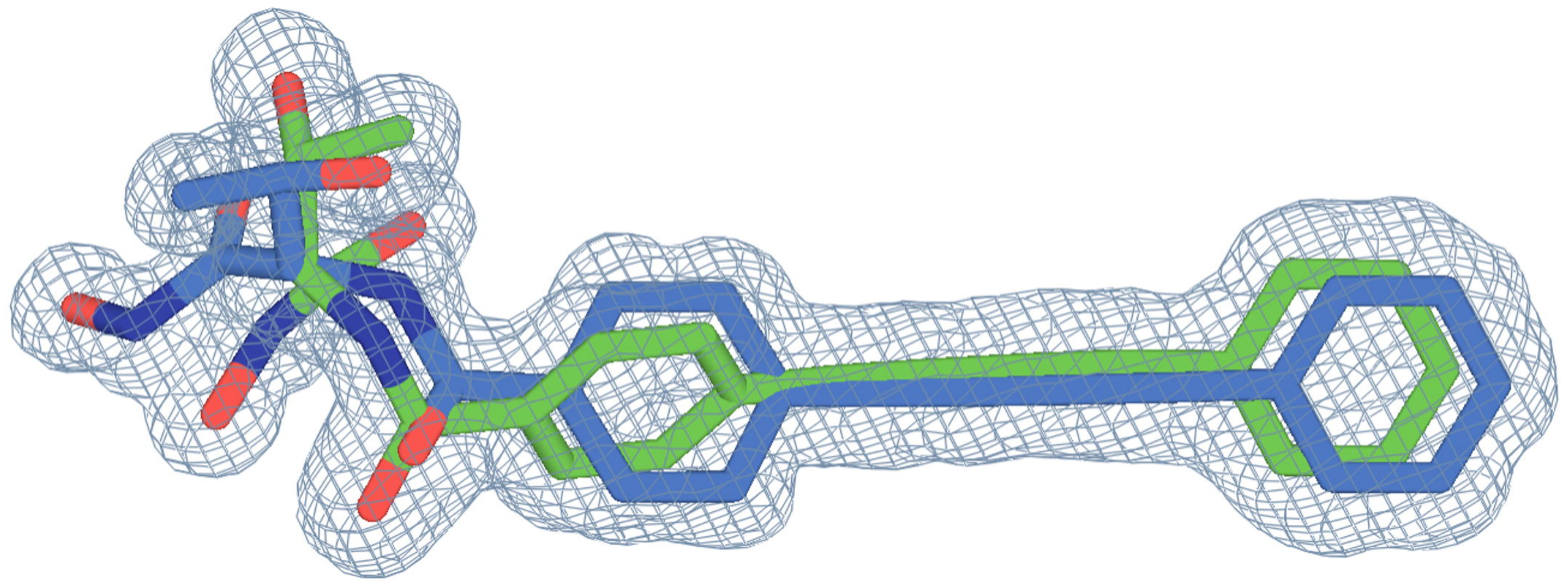

9BM

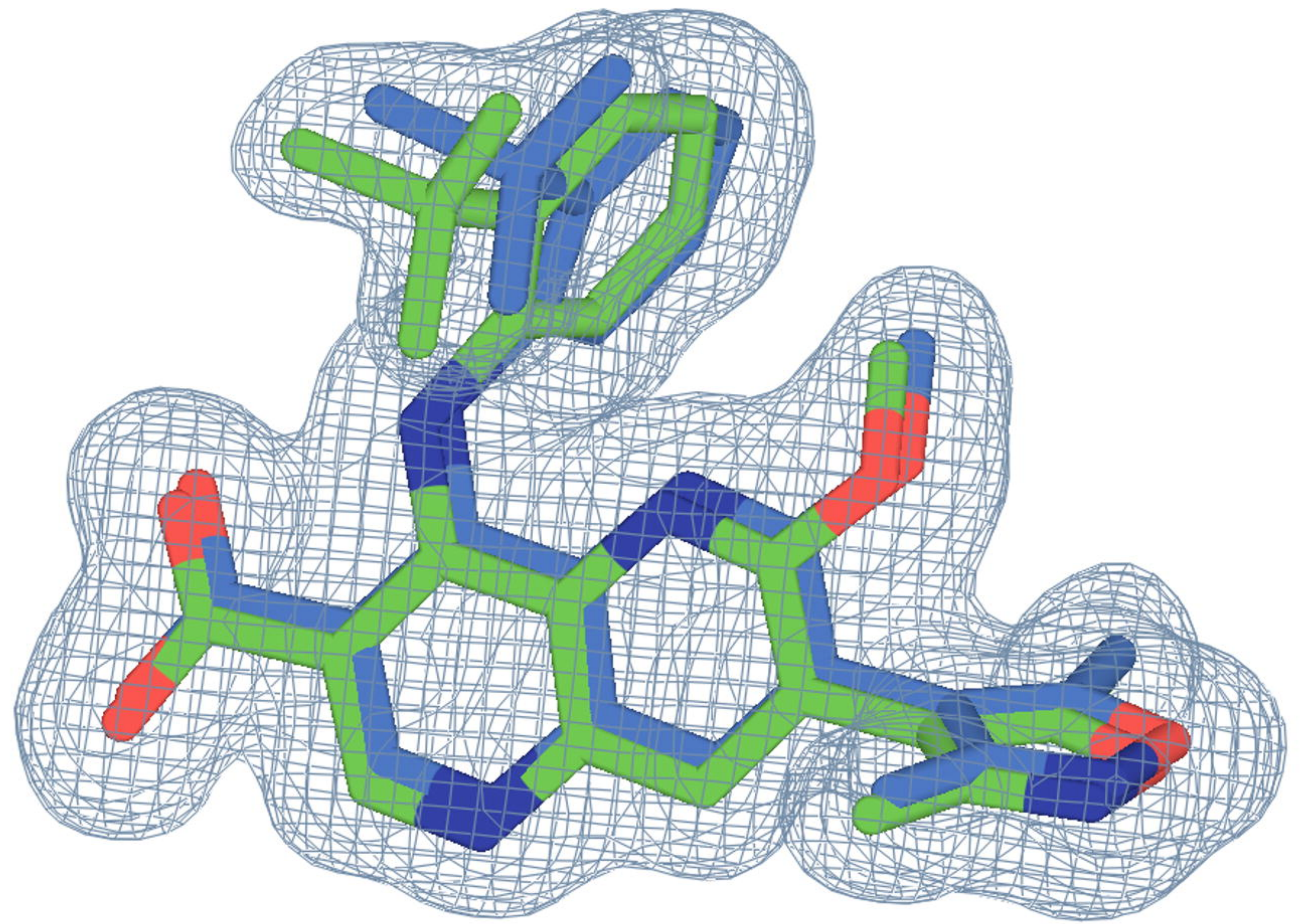

AR9

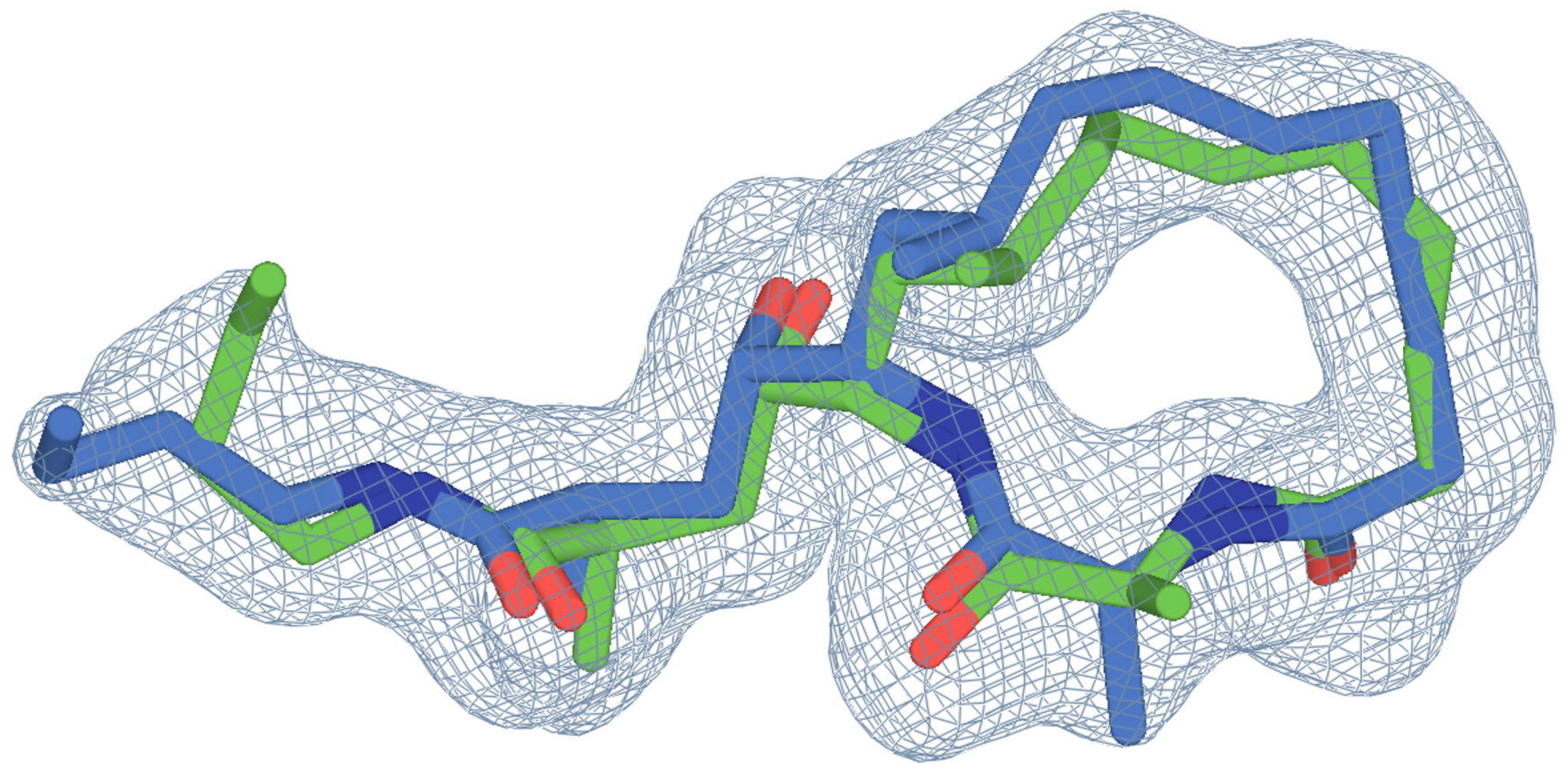

bioRxiv preprint doi: https://doi.org/10.1101/2024.09.20.613996; this version posted April 30, 2025. The copyright holder for this preprint (which was not certified by peer review) is the author/funder, who has granted bioRxiv a license to display the preprint in perpetuity. It is made available under aCC-BY 4.0 International license.

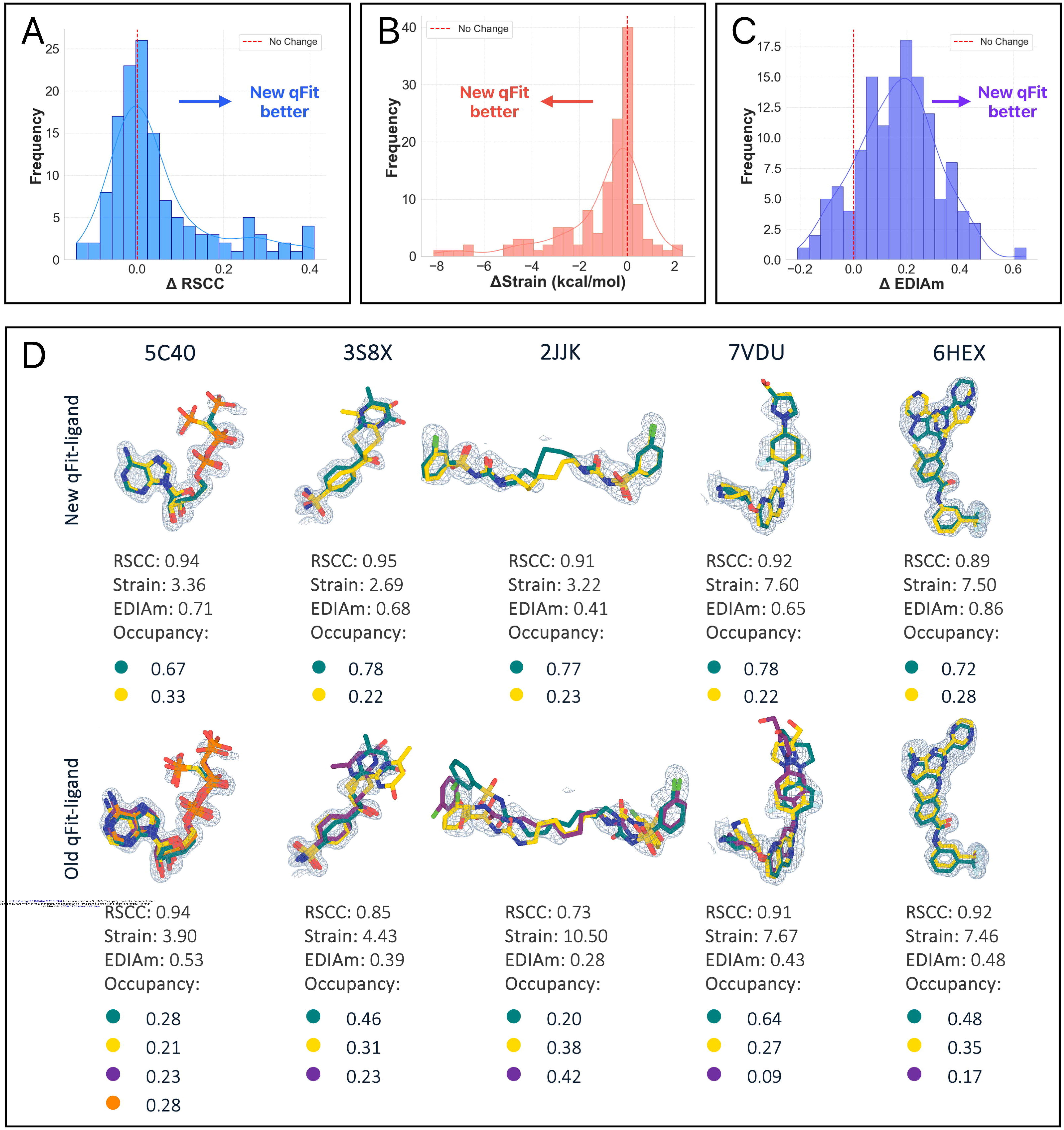

bioRxiv preprint doi: https://doi.org/10.1101/2024.09.20.613996; this version posted April 30, 2025. The copyright holder for this preprint (which was not certified by peer review) is the author/funder, who has granted bioRxiv a license to display the preprint in perpetuity. It is made available under aCC-BY 4.0 International license.

PDB: 7HHU

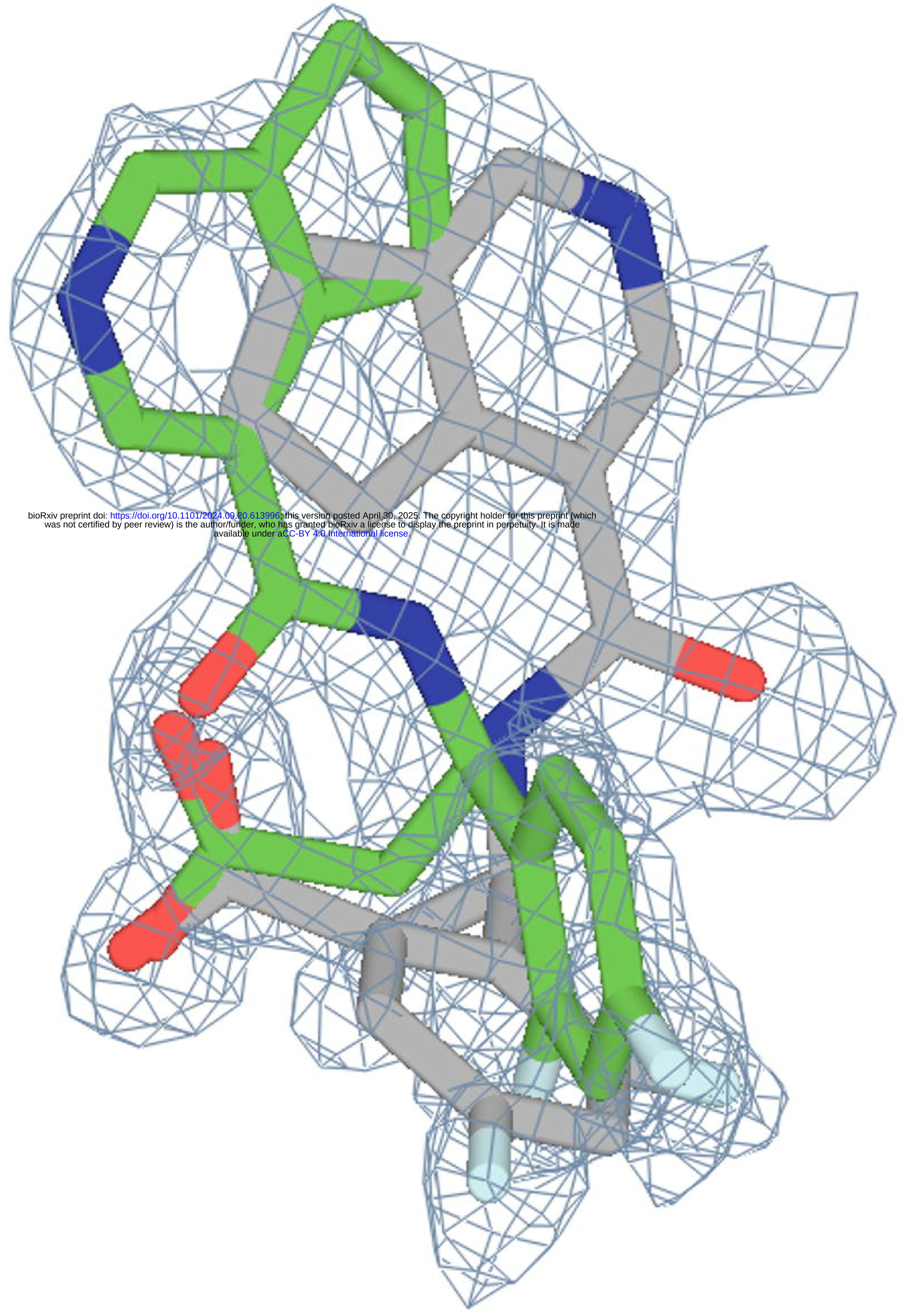

bioRxiv preprint doi: https://doi.org/10.1101/2024.09.20.613996; this version posted April 30, 2025. The copyright holder for this preprint (which was not certified by peer review) is the author/funder, who has granted bioRxiv a license to display the preprint in perpetuity. It is made available under aCC-BY 4.0 International license.

Deposited

RMSD between ‘A’ and ‘B’: 2.37

RSCC: 0.59
Strain: 10.05
EDIAm: 0.66
Occupancy:

- ● 1.00
- ● 1.00

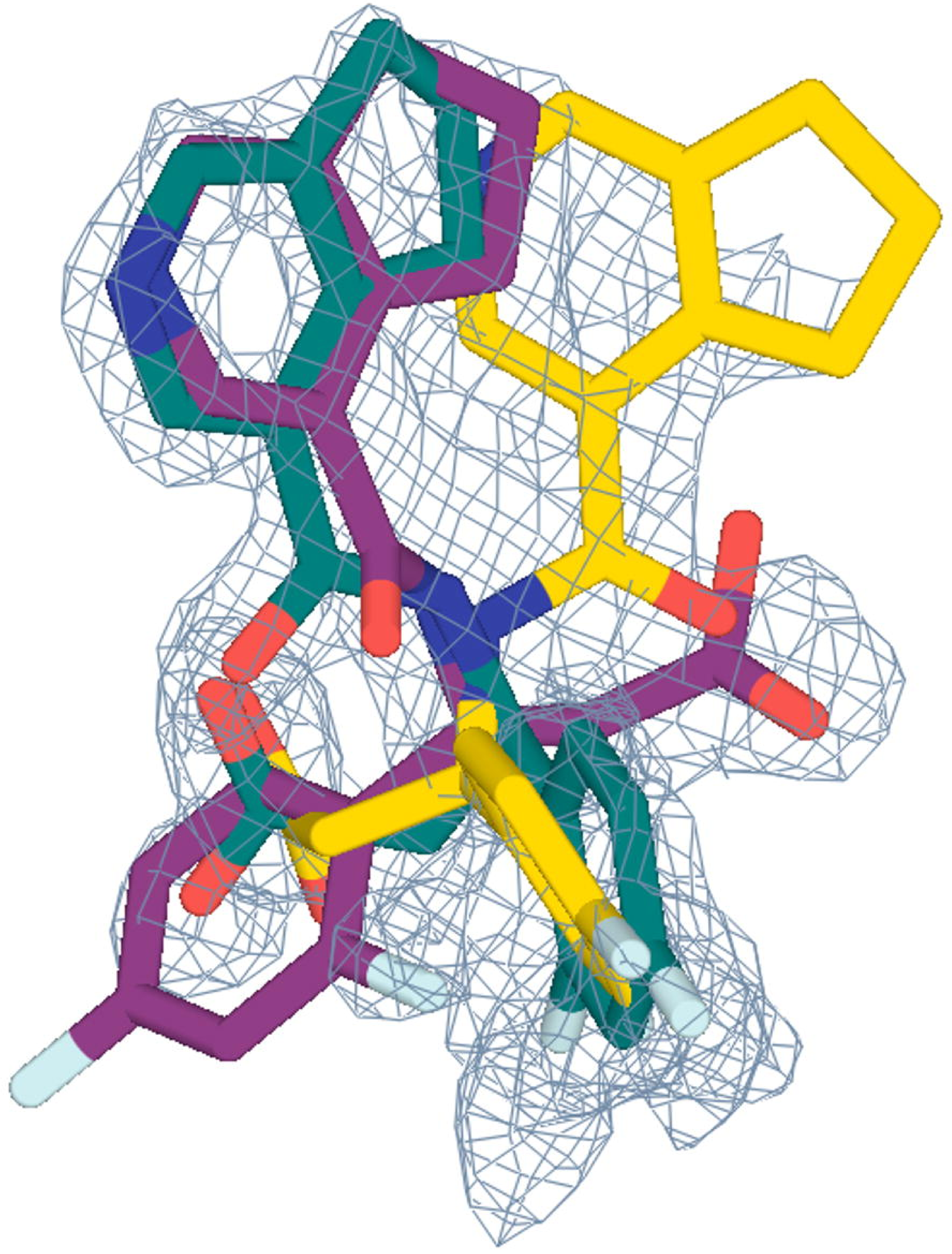

qFit-ligand

RMSD to deposited ‘B’: 0.70

RSCC: 0.62
Strain: 7.60
EDIAm: 0.55
Occupancy:

- ● 0.60
- ● 0.20
- ● 0.20

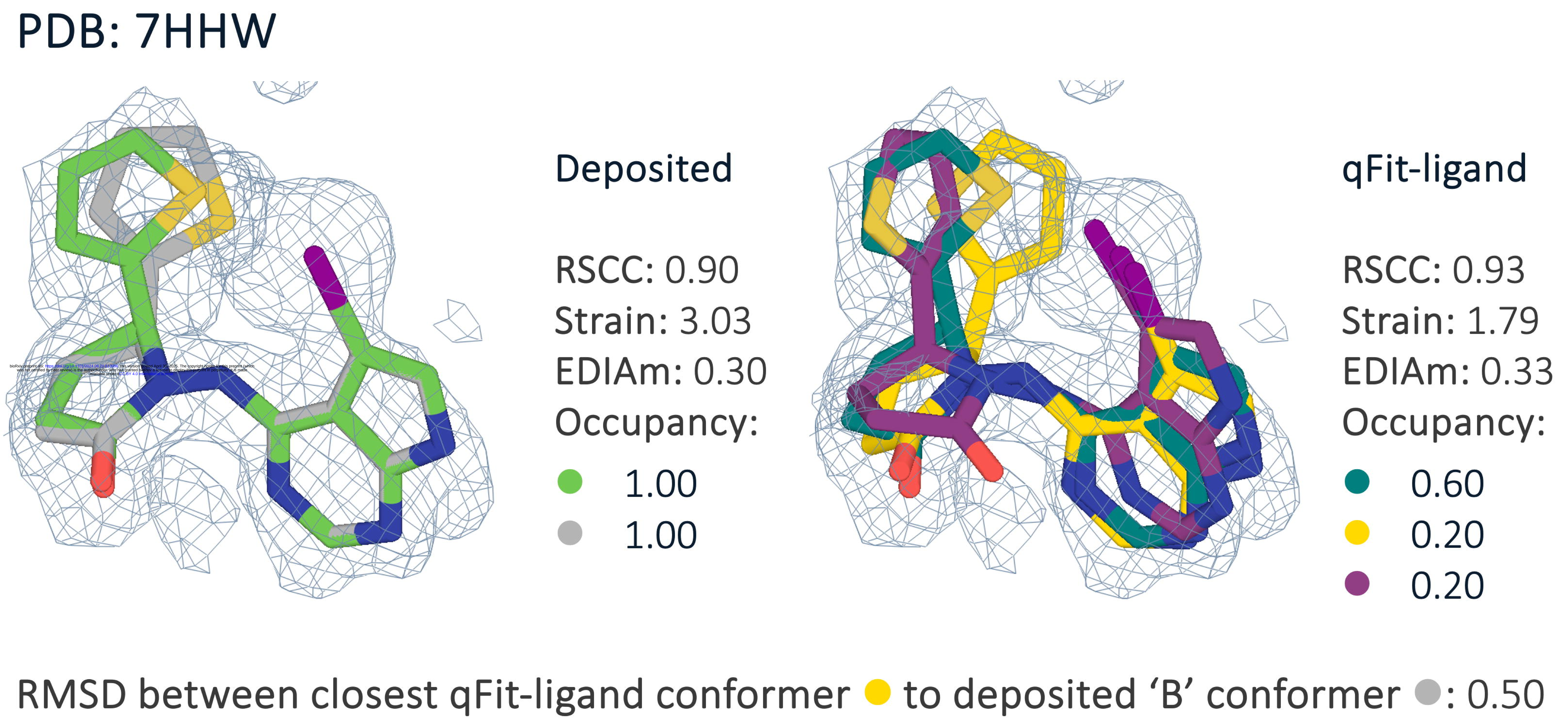

bioRxiv preprint doi: https://doi.org/10.1101/2024.09.20.613996; this version posted April 30, 2025. The copyright holder for this preprint (which was not certified by peer review) is the author/funder, who has granted bioRxiv a license to display the preprint in perpetuity. It is made available under aCC-BY 4.0 International license.

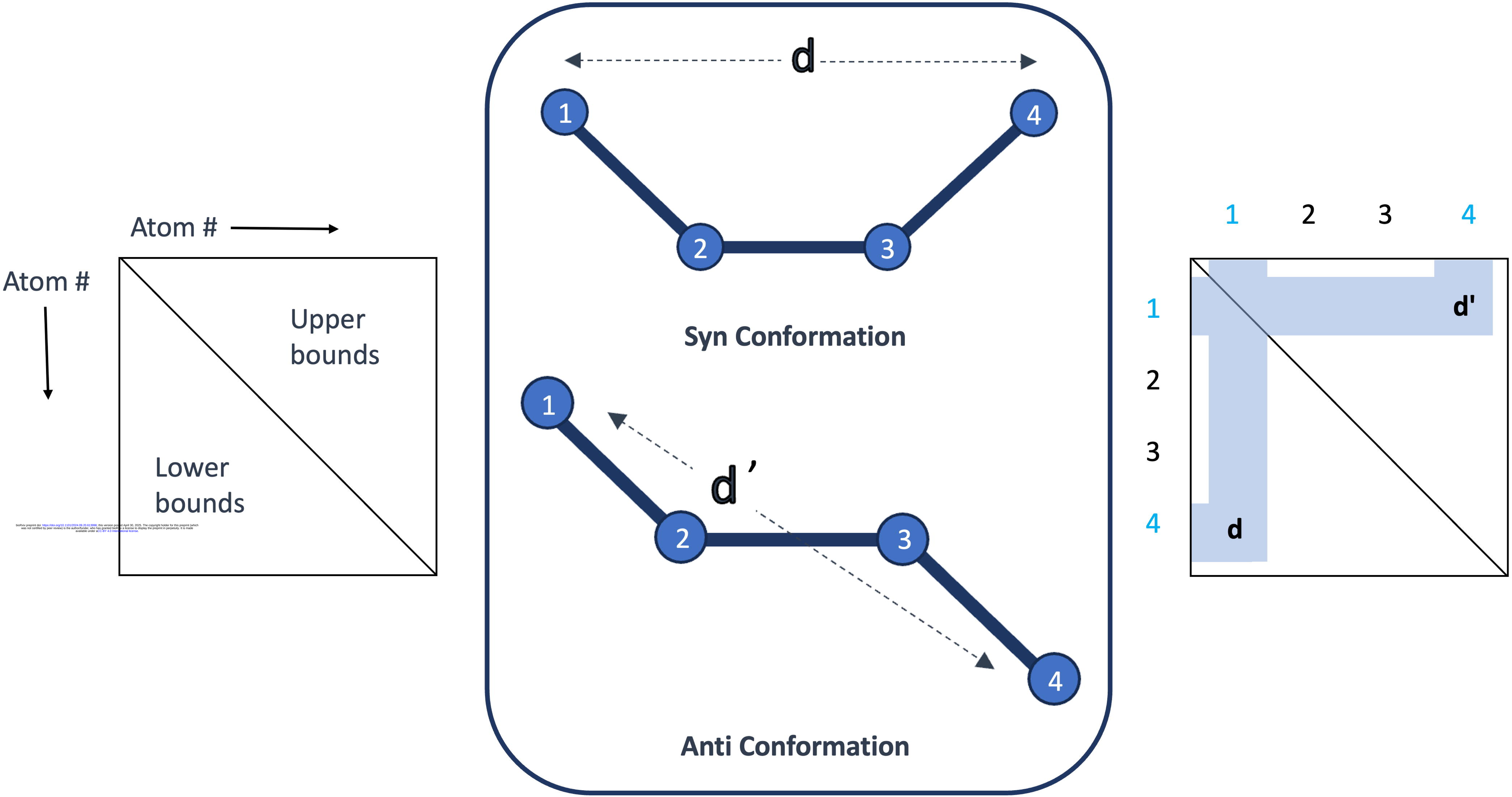

bioRxiv preprint doi: https://doi.org/10.1101/2024.09.20.613996; this version posted April 30, 2025. The copyright holder for this preprint (which was not certified by peer review) is the author/funder, who has granted bioRxiv a license to display the preprint in perpetuity. It is made available under aCC-BY 4.0 International license.

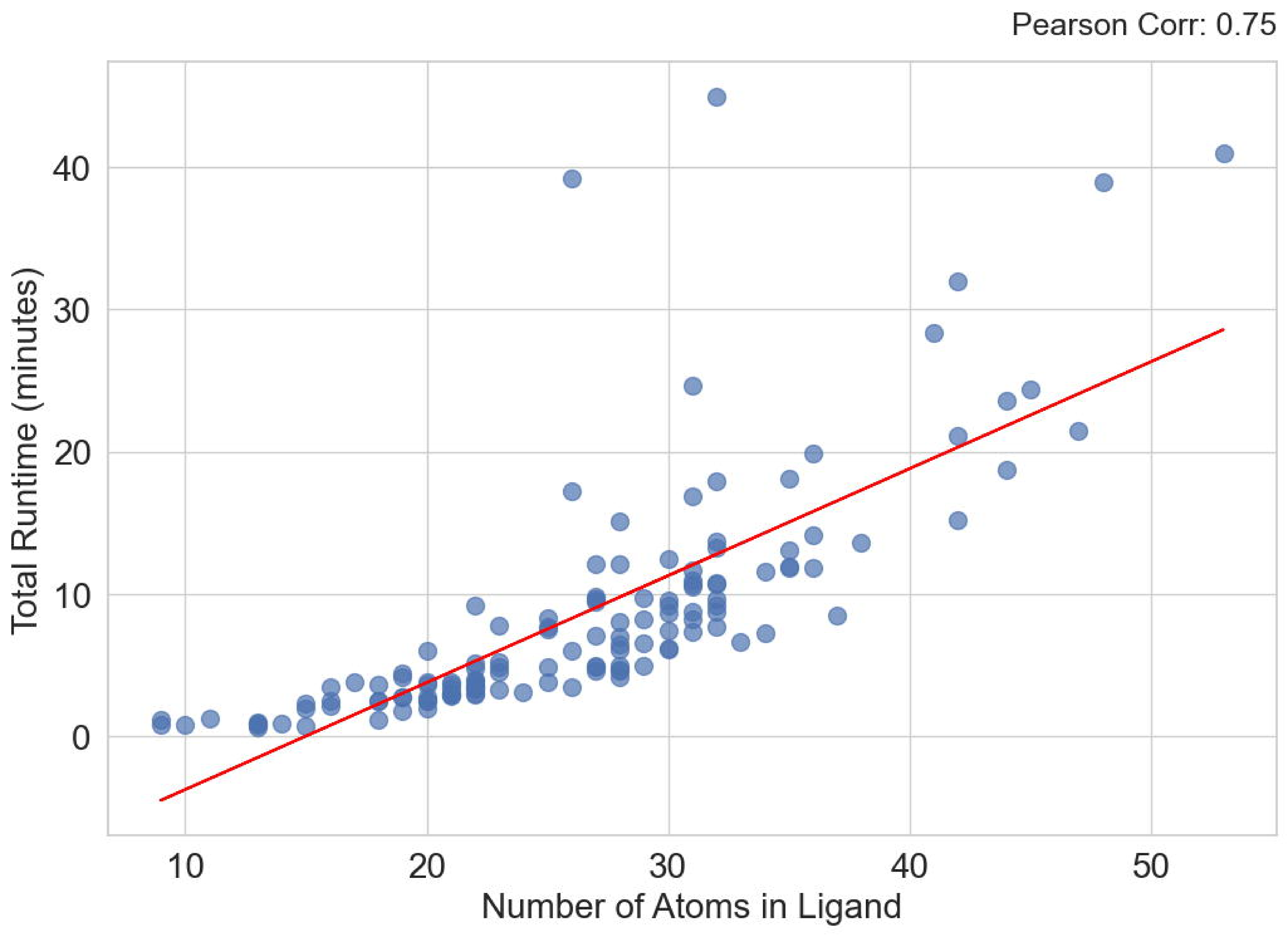
Pearson Corr: 0.75
Total Runtime (minutes)
0
10
20
30
40
10
20
30
40
50
Number of Atoms in Ligand

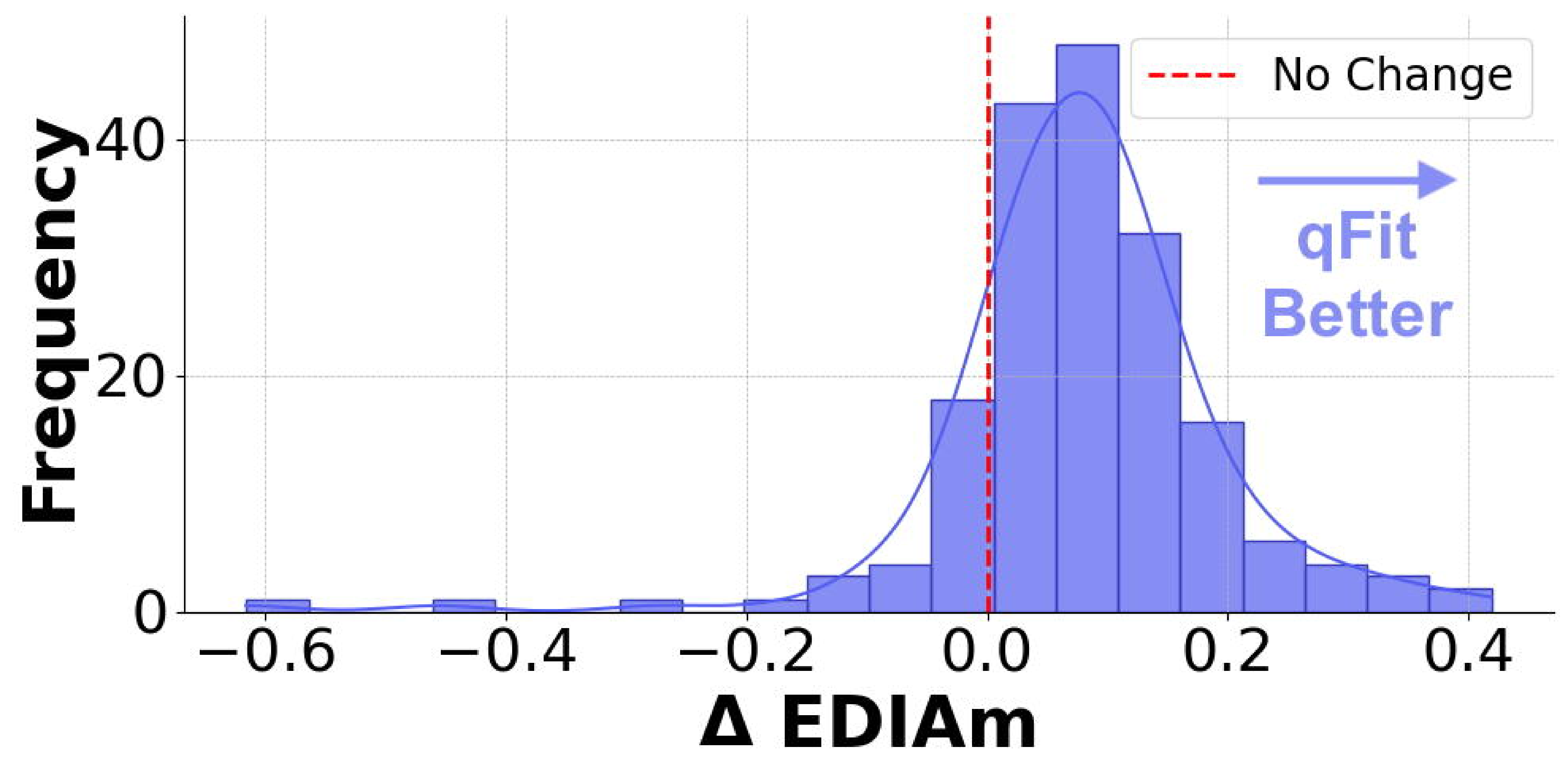
No Change
qFit
Better
Frequency
0
20
40
−0.6
−0.4
−0.2
0.0
0.2
0.4
Δ EDIAm

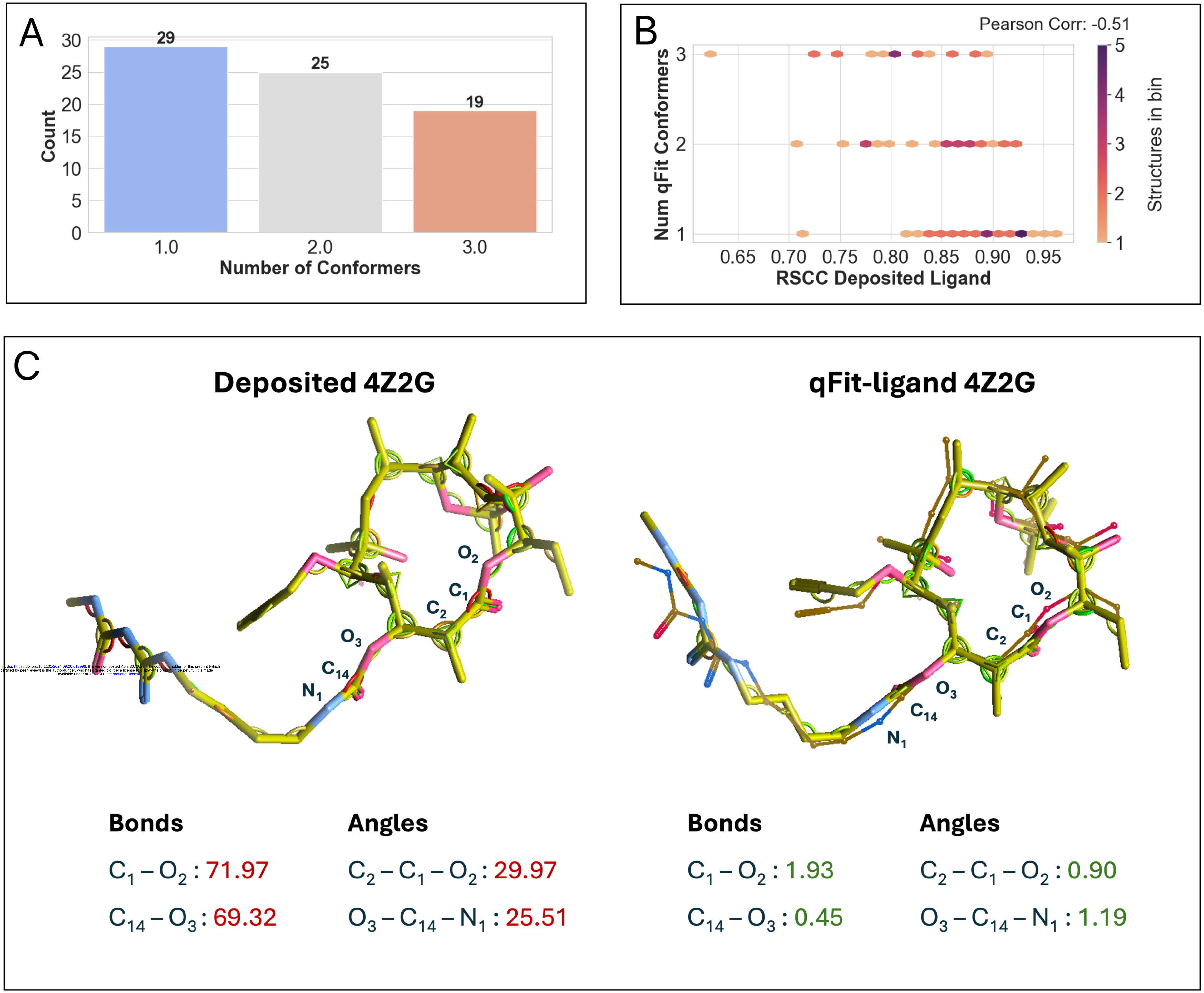

bioRxiv preprint doi: https://doi.org/10.1101/2024.09.20.613996; this version posted April 30, 2025. The copyright holder for this preprint (which was not certified by peer review) is the author/funder, who has granted bioRxiv a license to display the preprint in perpetuity. It is made available under aCC-BY 4.0 International license.

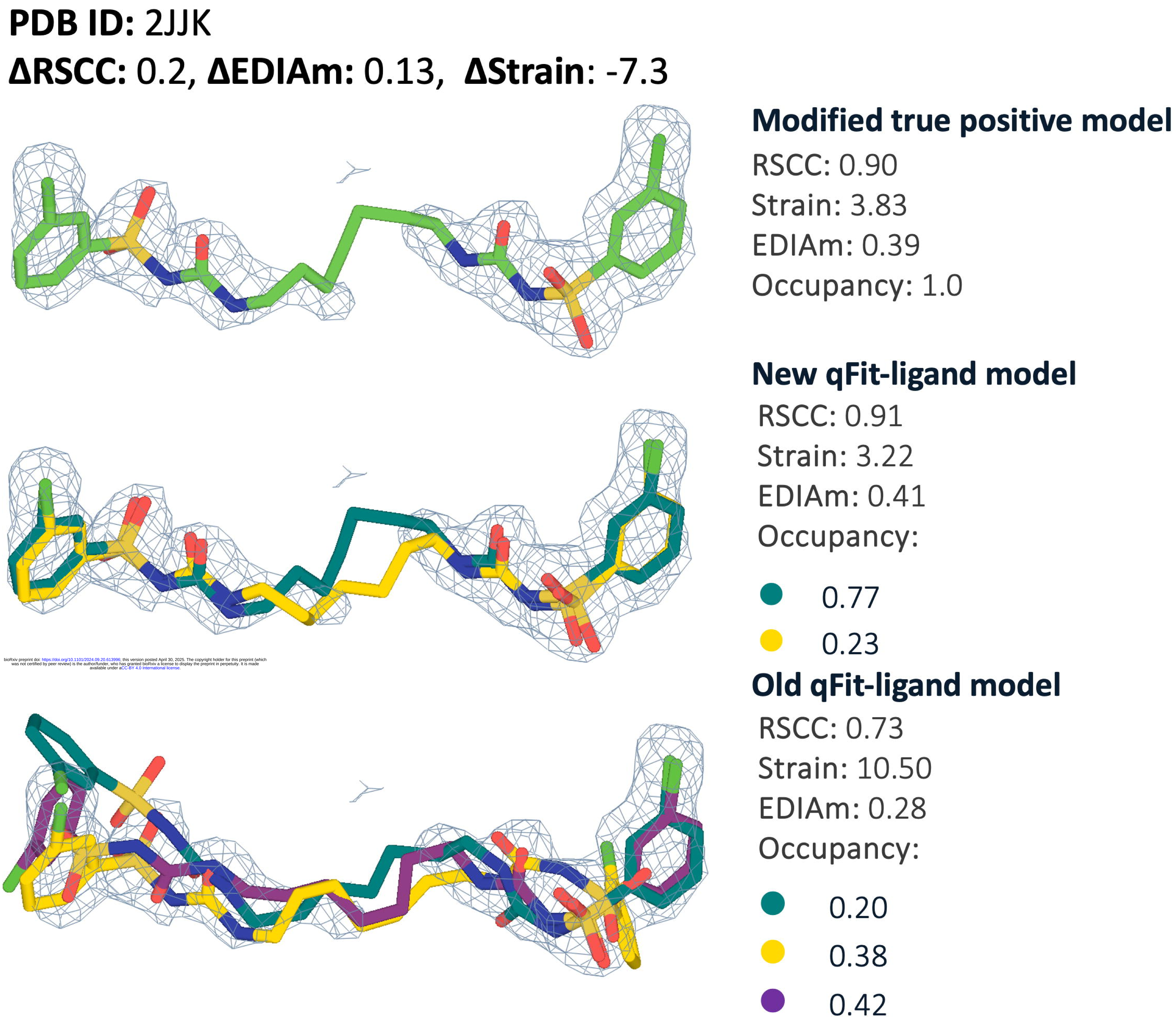

bioRxiv preprint doi: https://doi.org/10.1101/2024.09.20.613996; this version posted April 30, 2025. The copyright holder for this preprint (which was not certified by peer review) is the author/funder, who has granted bioRxiv a license to display the preprint in perpetuity. It is made available under aCC-BY 4.0 International license.

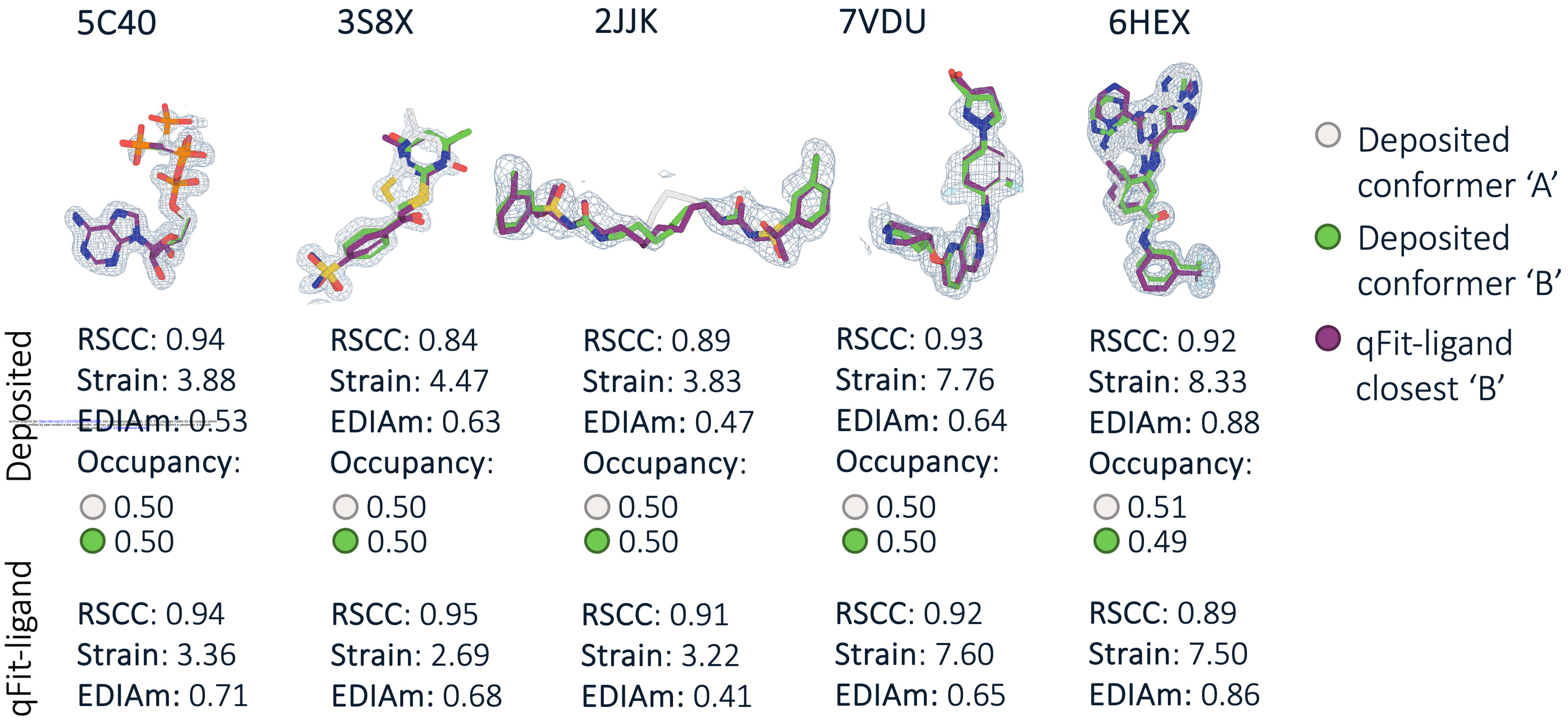

bioRxiv preprint doi: https://doi.org/10.1101/2024.09.20.613996; this version posted April 30, 2025. The copyright holder for this preprint (which was not certified by peer review) is the author/funder, who has granted bioRxiv a license to display the preprint in perpetuity. It is made available under aCC-BY 4.0 International license.

bioRxiv preprint doi: https://doi.org/10.1101/2024.09.20.613996; this version posted April 30, 2025. The copyright holder for this preprint (which was not certified by peer review) is the author/funder, who has granted bioRxiv a license to display the preprint in perpetuity. It is made available under aCC-BY 4.0 International license.

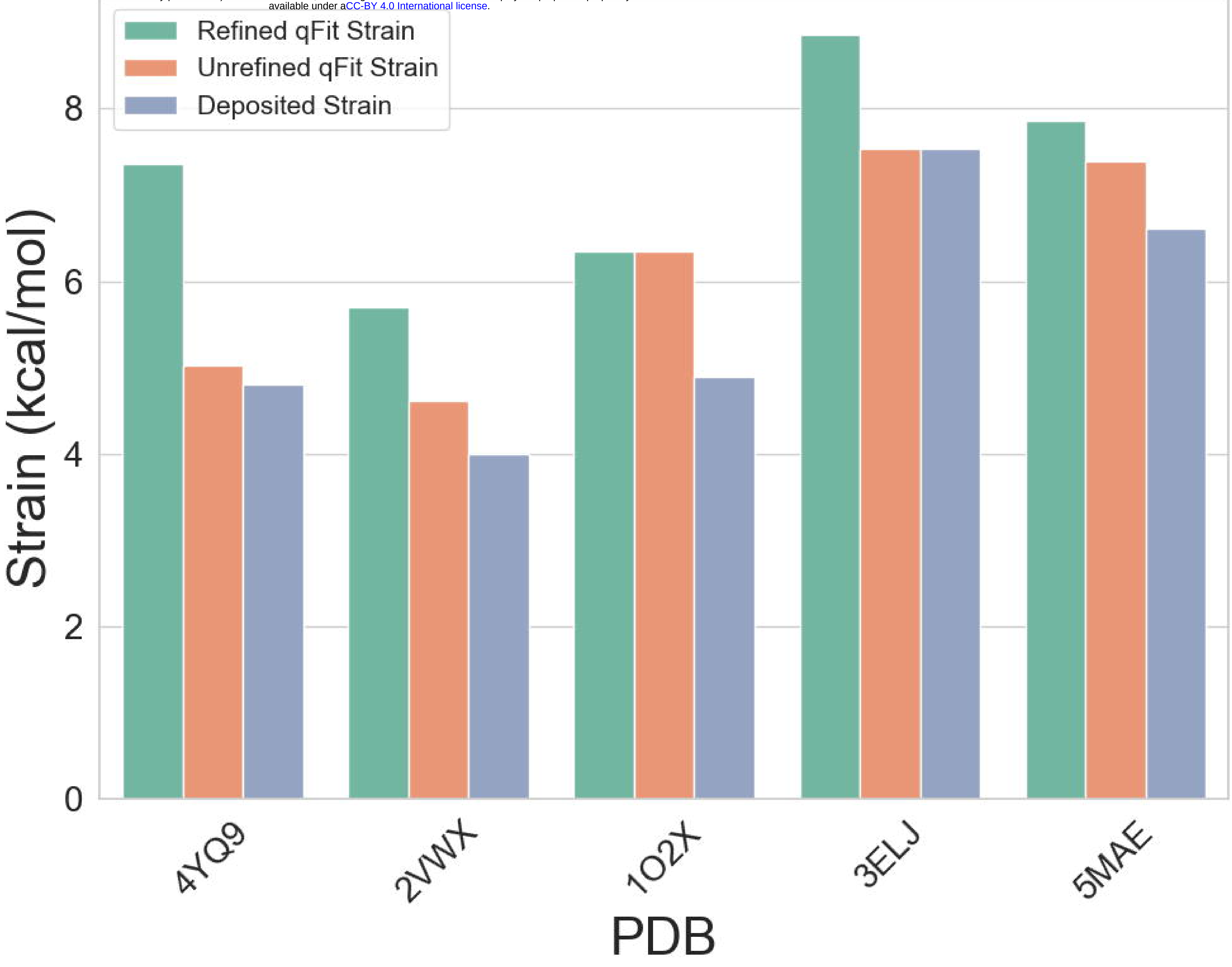

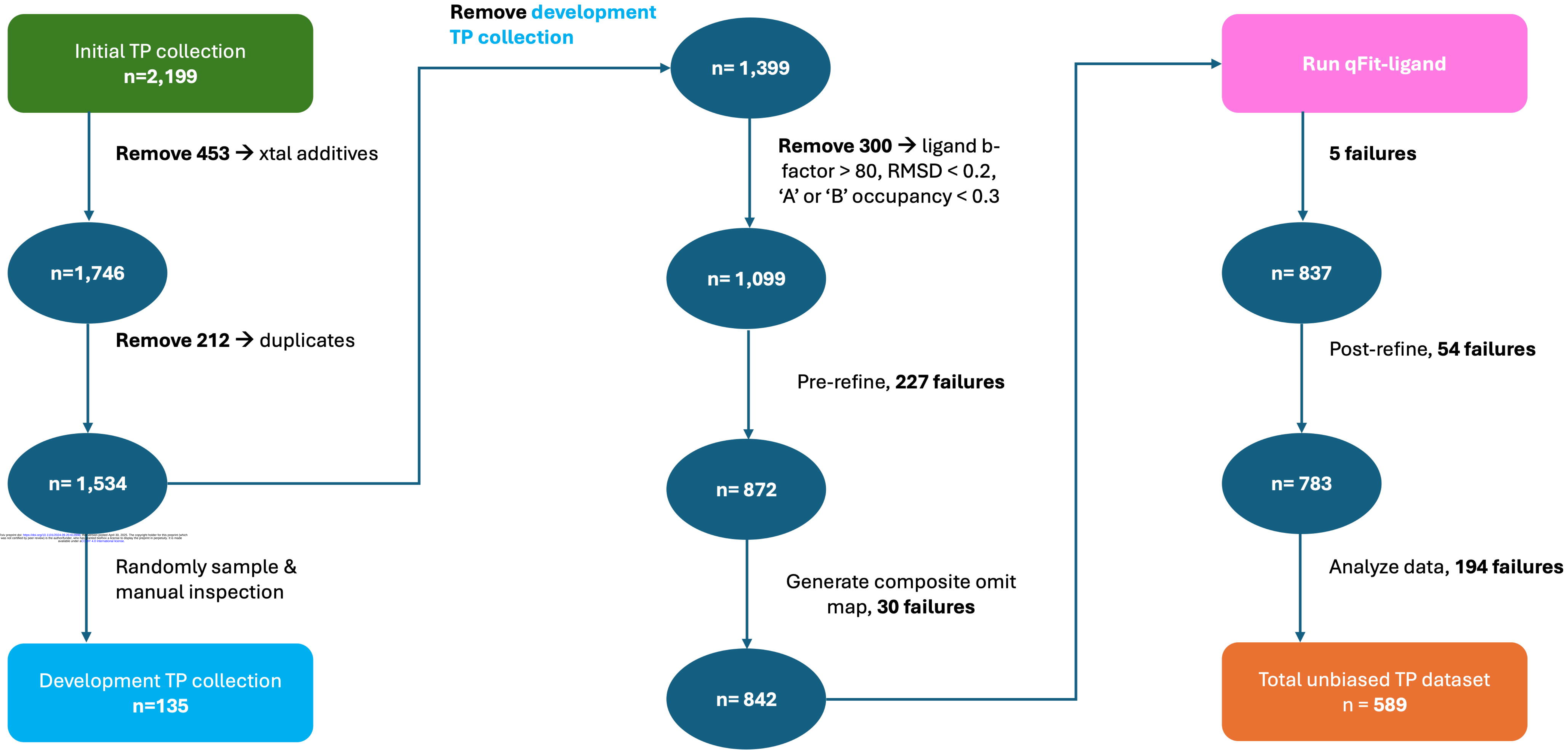

bioRxiv preprint doi: https://doi.org/10.1101/2024.09.20.613996; this version posted April 30, 2025. The copyright holder for this preprint (which was not certified by peer review) is the author/funder, who has granted bioRxiv a license to display the preprint in perpetuity. It is made available under aCC-BY 4.0 International license.
