# Supplementary material for: Expanding Automated Multiconformer Ligand Modeling to Macrocycles and Fragments
Source: bioRxiv. 2024 Sep 23:2024.09.20.613996. Preprint. [Version 1] doi: 10.1101/2024.09.20.613996 (PMC11463535; doi:10.1101/2024.09.20.613996)
Supplement: Supplementary file 1 [file NIHPP2024.09.20.613996v1-supplement-5.pdf]

bioRxiv preprint doi: https://doi.org/10.1101/2024.09.20.613996; this version posted September 23, 2024. The copyright holder for this preprint (which was not certified by peer review) is the author/funder, who has granted bioRxiv a license to display the preprint in perpetuity. It is made available under aCC-BY 4.0 International license.

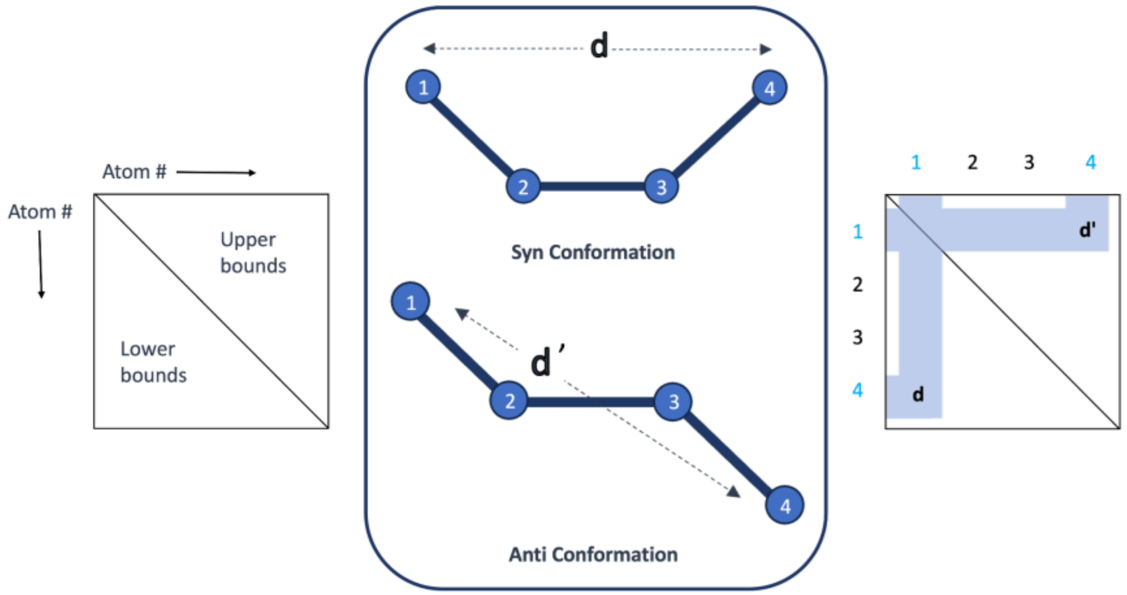

Figure S1. RDKit determines a distance bounds matrix for a molecule by establishing upper and lower bounds for interatomic distances. These bounds are informed by experimental data and chemical knowledge of bond length, angle, and dihedral angle preferences obtained from the Cambridge Structural Database. Within a torsion angle formed by four atoms, the minimum distance between atoms 1 and 4 corresponds to the syn conformation, and the maximum distance corresponds to the anti conformation. These specific distances, d for syn and d' for anti, are recorded in the bounds matrix as the lower and upper bounds, respectively. This is performed for every distance between each atom in the molecule. Randomly sampling these bounds with RDKit's implementation of ETKDG gives rise to different conformations of the torsion angle.

bioRxiv preprint doi: https://doi.org/10.1101/2024.09.20.613996; this version posted September 23, 2024. The copyright holder for this preprint (which was not certified by peer review) is the author/funder, who has granted bioRxiv a license to display the preprint in perpetuity. It is made available under aCC-BY 4.0 International license.

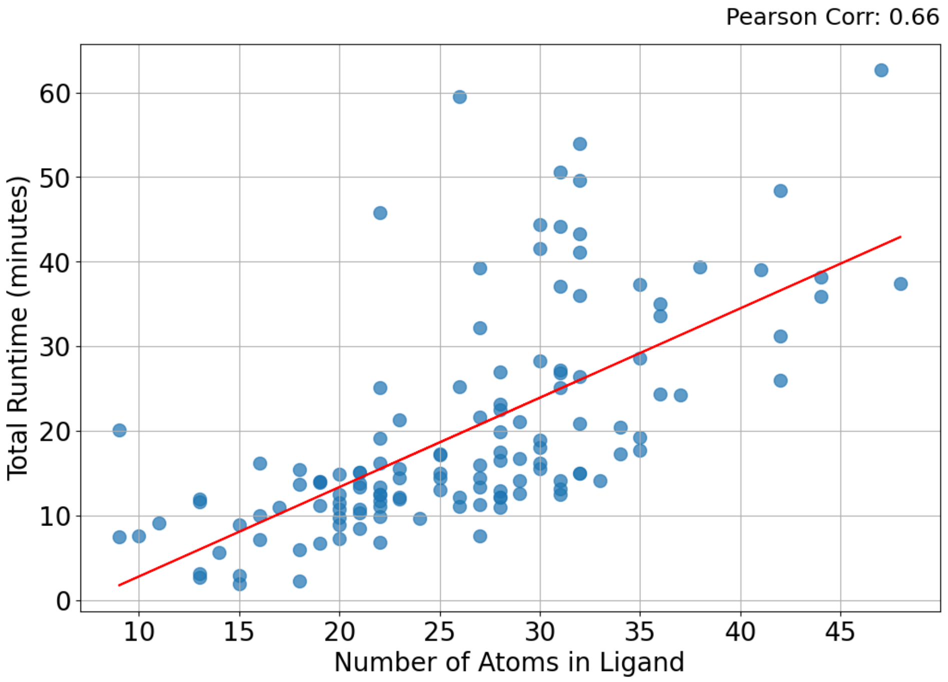

Figure S2. Correlation between the number of atoms in the input ligand and total qFit-ligand runtime. A strong Pearson correlation of 0.66 indicates that as you increase the size of your input molecule, qFit-ligand will take longer to run.

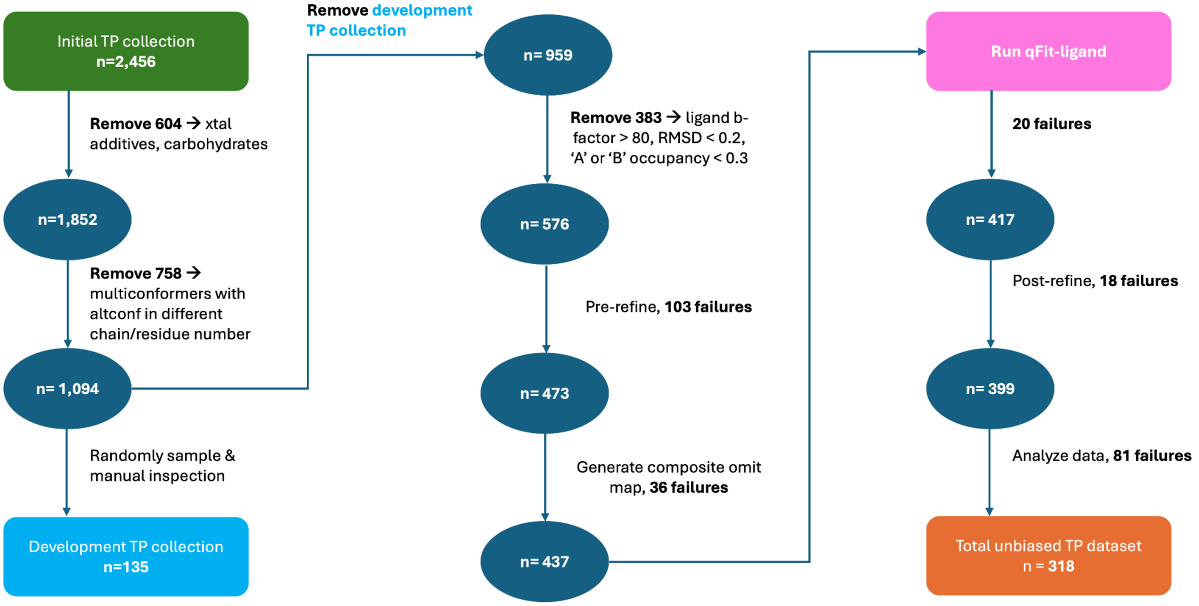

Figure S3. Construction of the development true positive dataset and the unbiased true positive dataset.

bioRxiv preprint doi: https://doi.org/10.1101/2024.09.20.613996; this version posted September 23, 2024. The copyright holder for this preprint (which was not certified by peer review) is the author/funder, who has granted bioRxiv a license to display the preprint in perpetuity. It is made available under aCC-BY 4.0 International license.

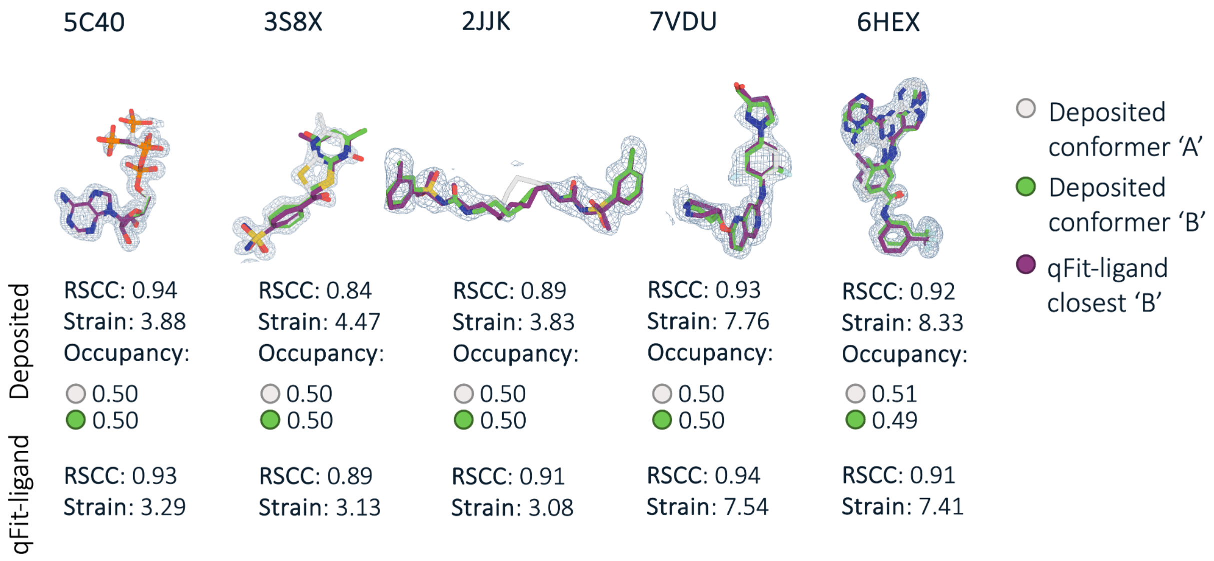

Figure S4. Original (unmodified) multiconformer true positives compared to qFit-ligand conformers. The deposited 'A' conformer is shown in gray and the deposited 'B' conformer in green. The qFit-ligand conformer closest to the deposited 'B' is shown in purple. This demonstrates qFit-ligand's ability to accurately recapitulate the original deposited multiconformer model. The composite omit density map is contoured at 1σ for every structure.

bioRxiv preprint doi: https://doi.org/10.1101/2024.09.20.613996; this version posted September 23, 2024. The copyright holder for this preprint (which was not certified by peer review) is the author/funder, who has granted bioRxiv a license to display the preprint in perpetuity. It is made available under aCC-BY 4.0 International license.

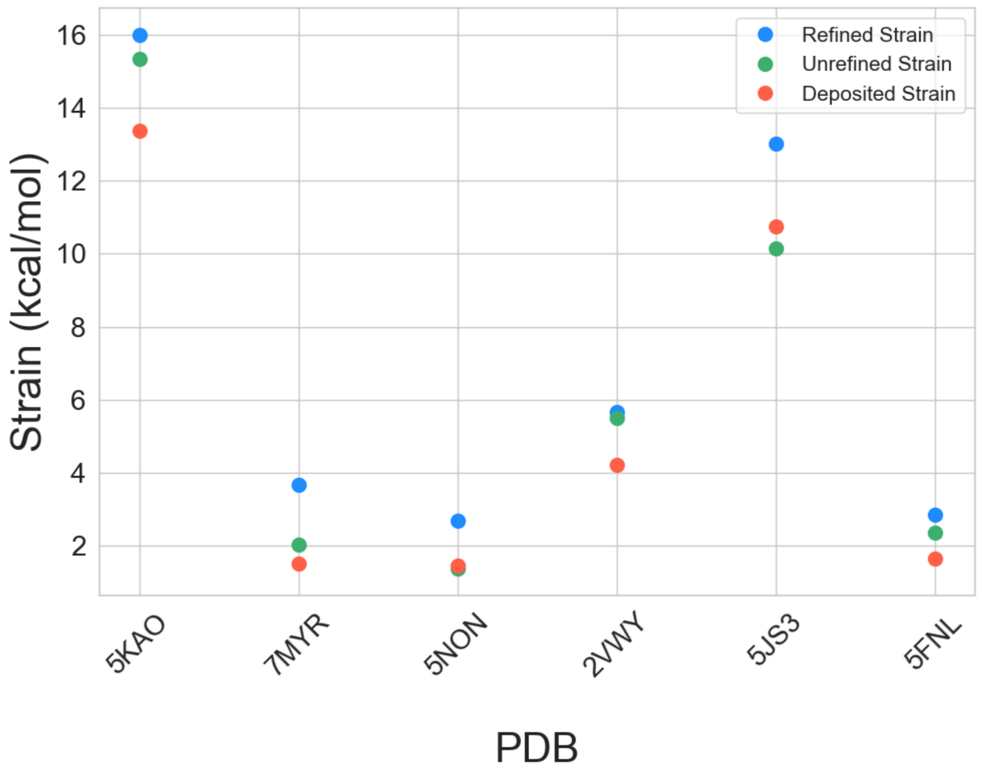

Figure S5. Comparison of torsion strain between qFit-ligand models before and after refinement, as well as the deposited structures. The five outlier structures where the refined qFit-ligand model strain exceeded the deposited model strain by more than 1 kcal/mol are highlighted.

bioRxiv preprint doi: https://doi.org/10.1101/2024.09.20.613996; this version posted September 23, 2024. The copyright holder for this preprint (which was not certified by peer review) is the author/funder, who has granted bioRxiv a license to display the preprint in perpetuity. It is made available under aCC-BY 4.0 International license.

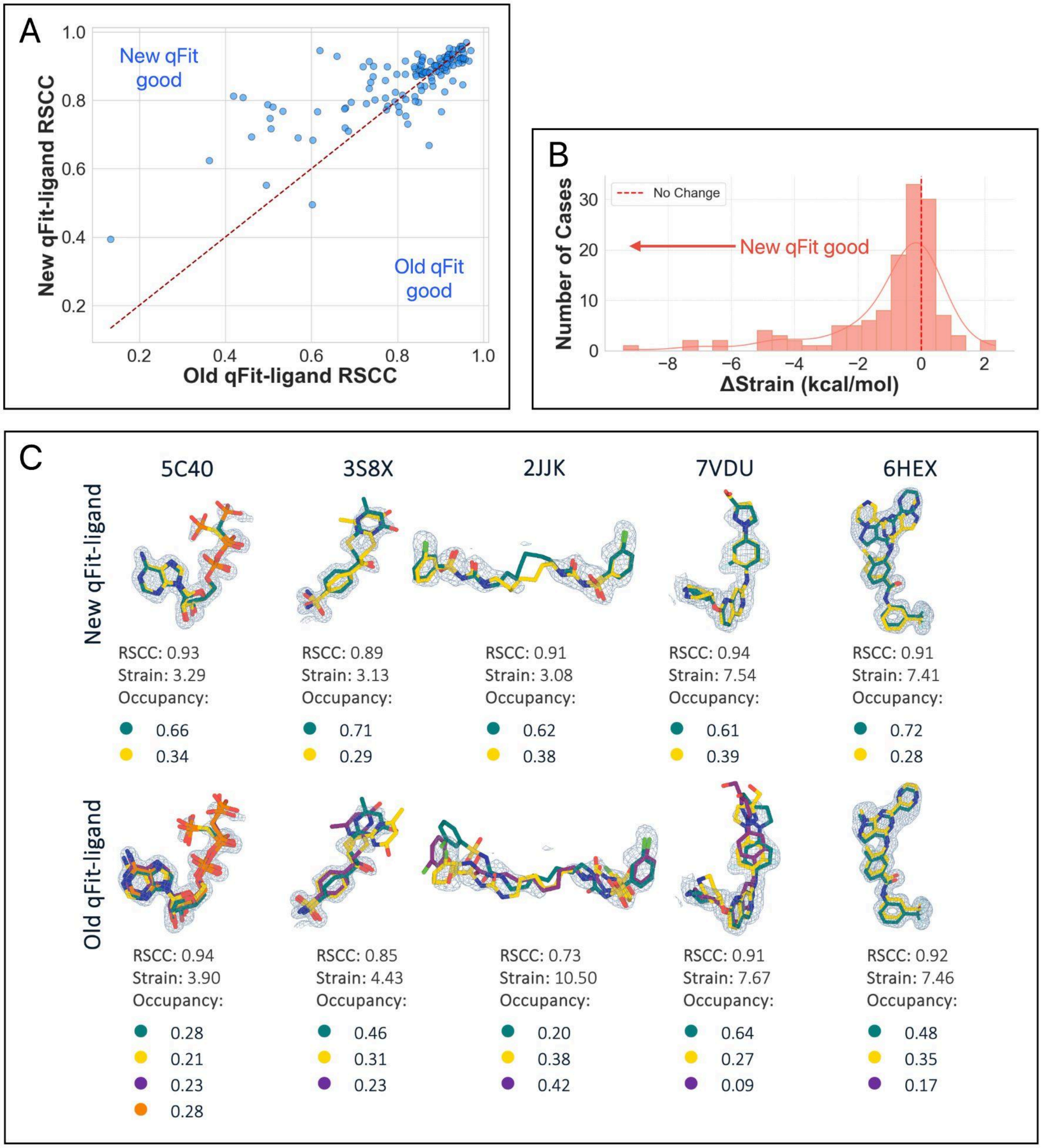

Figure S6. Performance comparison of new and old qFit-ligand algorithms. (A) RSCC of new versus old qFit-ligand predicted conformations across the true positive dataset. Points above the diagonal line are for structures where the new qFit-ligand model has a higher (better) RSCC. (B) Torsion strain of new versus old qFit-ligand predicted conformations across the true positive dataset. Bars to the left of the vertical line are for structures where the new qFit-ligand model has improved (lower) internal strain. (C) Gallery of examples for which the updated qFit-ligand models are both higher in RSCC

bioRxiv preprint doi: https://doi.org/10.1101/2024.09.20.613996; this version posted September 23, 2024. The copyright holder for this preprint (which was not certified by peer review) is the author/funder, who has granted bioRxiv a license to display the preprint in perpetuity. It is made available under aCC-BY 4.0 International license.

and lower in strain compared to the old qFit-ligand models. The composite omit density map is contoured at 1σ for every structure.

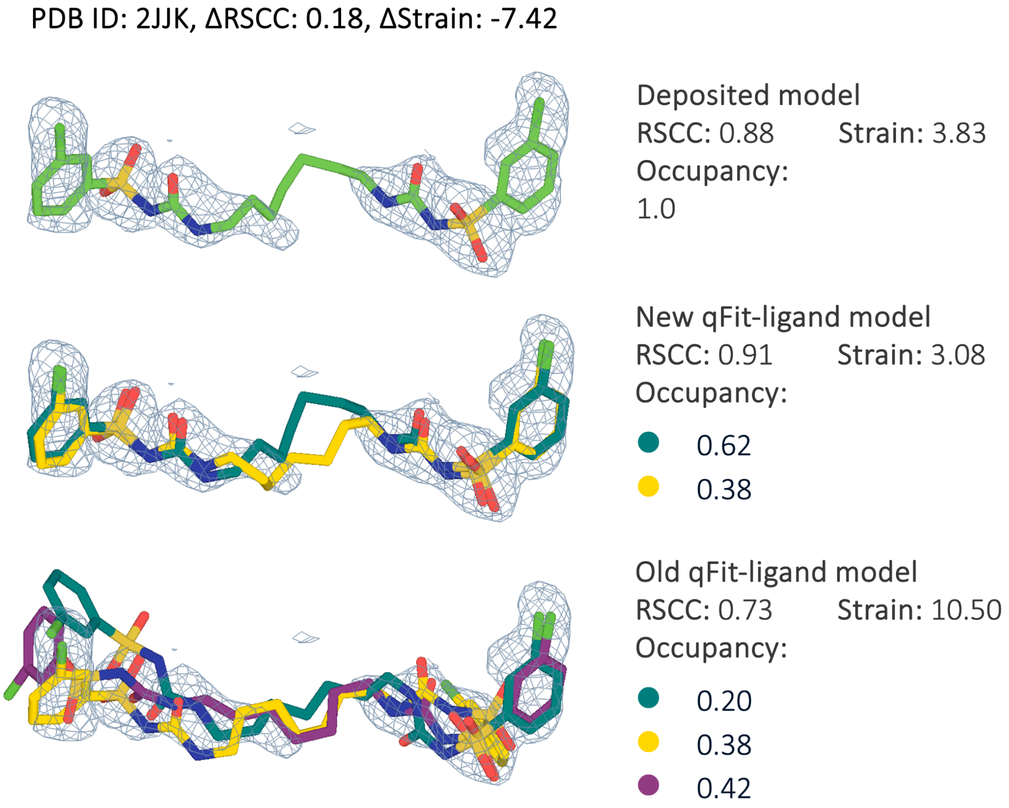

Figure S7. Modified true positive dataset comparison of new versus old qFit-ligand outlier cases. Modified true positive model (input for qFit-ligand), new qFit-ligand model, and old qFit-ligand model for PDB 2JJK, showing their respective RSCCs, strain, and conformer occupancies. This highlights significant improvements in both RSCC and strain. The composite omit density map is contoured at 1σ.

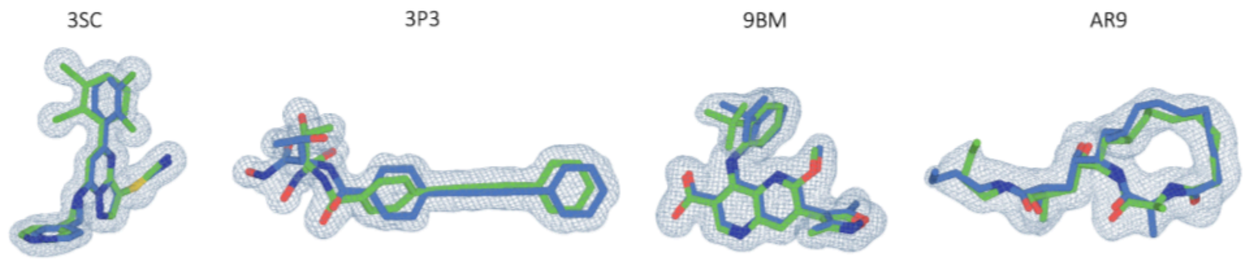

Figure S8. The four ligand multiconformer models from which our synthetic dataset was built. Here, they are shown at a map resolution of 0.8 Å at one sigma.

bioRxiv preprint doi: https://doi.org/10.1101/2024.09.20.613996; this version posted September 23, 2024. The copyright holder for this preprint (which was not certified by peer review) is the author/funder, who has granted bioRxiv a license to display the preprint in perpetuity. It is made available under aCC-BY 4.0 International license.

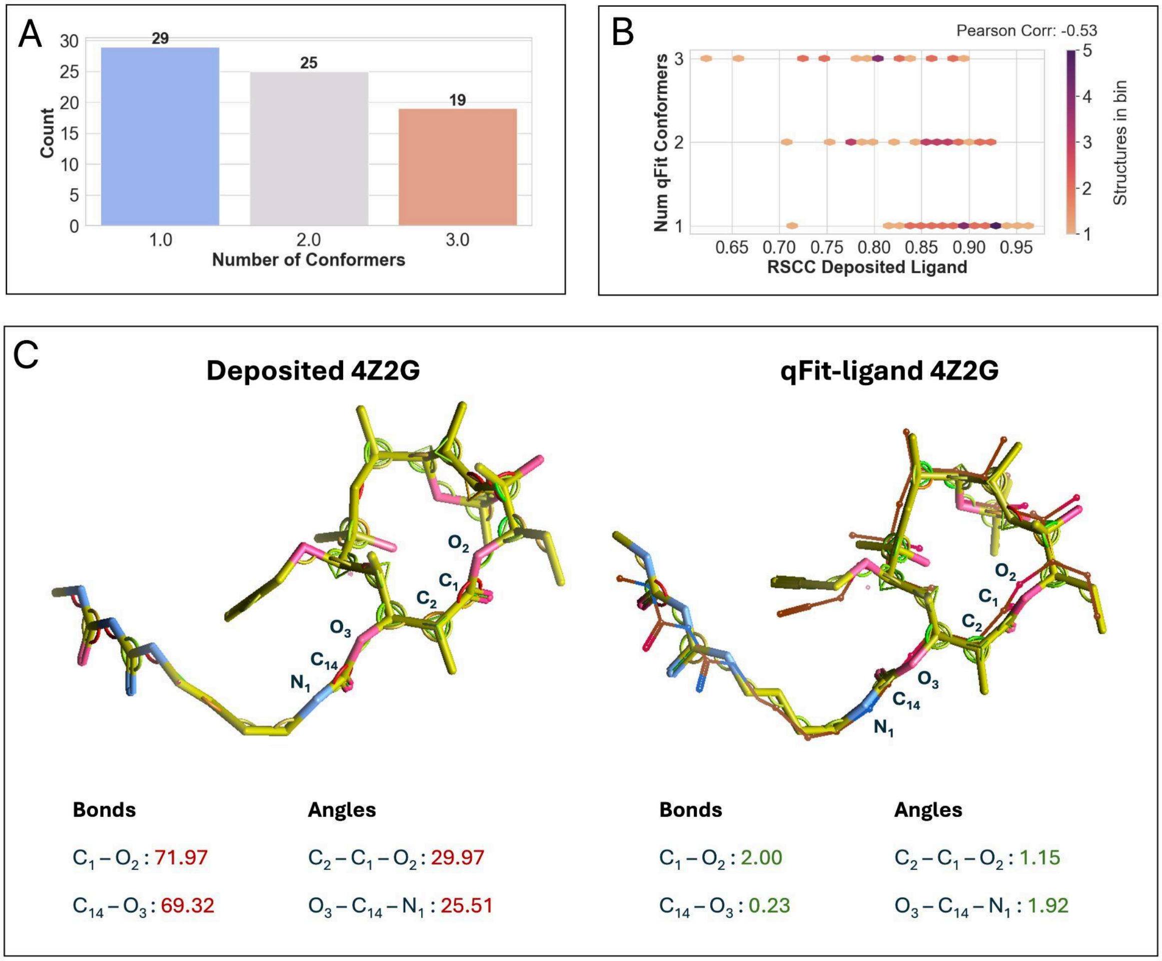

Figure S9. (A) Distribution of the number of conformers in qFit-ligand output models, showing varied conformer presence with a median of two conformers per structure. (B) Correlation between the number of conformers output by qFit-ligand and the RSCC of the input model. Higher input RSCC tends to yield a lower number of qFit-ligand conformers. (C) Comparison of strain between the single conformer deposited macrocycle and the qFit-ligand 'B' conformer for PDB 4Z2G using the COOT ligand distortion tool. The penalty scores for the two most distorted bonds and angles in the deposited model (left), compared to the same bonds and angles in the qFit-ligand 'B' conformer (right), demonstrating reduced strain in the alternate conformation.

bioRxiv preprint doi: https://doi.org/10.1101/2024.09.20.613996; this version posted September 23, 2024. The copyright holder for this preprint (which was not certified by peer review) is the author/funder, who has granted bioRxiv a license to display the preprint in perpetuity. It is made available under aCC-BY 4.0 International license.

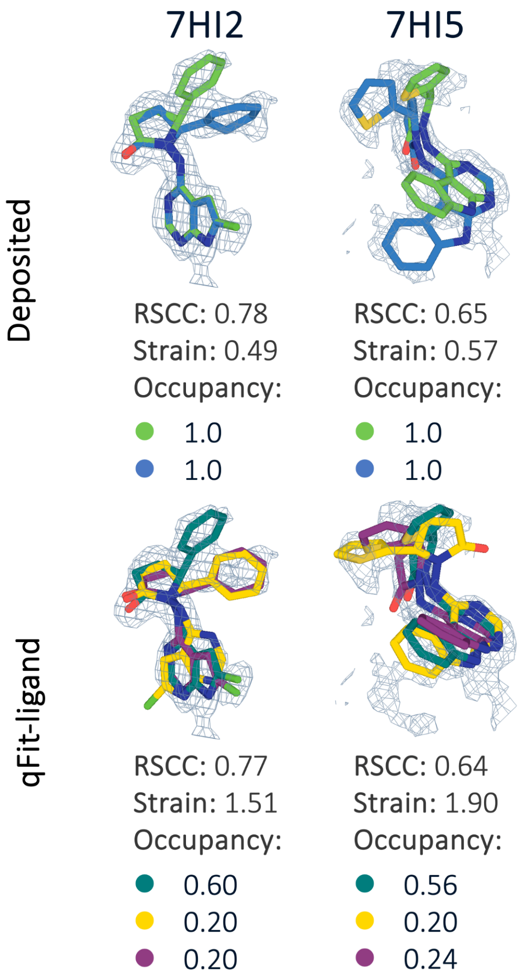

Figure S10. The two structures for which qFit-ligand decreases RSCC and increases torsional strain. The composite omit density map is contoured at 1σ for both structures.
